# Proton-FLASH: effects of ultra-high dose rate irradiation on an in-vivo mouse ear model

**DOI:** 10.1038/s41598-024-51951-6

**Published:** 2024-01-16

**Authors:** Sarah Rudigkeit, Thomas E. Schmid, Annique C. Dombrowsky, Jessica Stolz, Stefan Bartzsch, Ce-Belle Chen, Nicole Matejka, Matthias Sammer, Andreas Bergmaier, Günther Dollinger, Judith Reindl

**Affiliations:** 1 Institute of Applied Physics and Measurement Technologies, Universität der Bundeswehr München, Neubiberg, Germany; 2 Institute of Radiation Medicine (IRM), Helmholtz Zentrum München, Neuherberg, Germany; 3 Department of Radiooncology, School of Medicine, Klinikum Rechts der Isar, Technische Universität München, Munich, Germany; 4 Centre for Ion Beam Applications, Department of Physics, National University of Singapore, Singapore, Singapore; 5 Singapore Synchrotron Light Source, National University of Singapore, Singapore, Singapore

**Keywords:** Techniques and instrumentation, Preclinical research

## Abstract

FLASH-radiotherapy may provide significant sparing of healthy tissue through ultra-high dose rates in protons, electrons, and x-rays while maintaining the tumor control. Key factors for the FLASH effect might be oxygen depletion, the immune system, and the irradiated blood volume, but none could be fully confirmed yet. Therefore, further investigations are necessary. We investigated the protective (tissue sparing) effect of FLASH in proton treatment using an in-vivo mouse ear model. The right ears of Balb/c mice were irradiated with 20 MeV protons at the ion microprobe SNAKE in Garching near Munich by using three dose rates (Conv = 0.06 Gy/s, Flash9 = 9.3 Gy/s and Flash930 = 930 Gy/s) at a total dose of 23 Gy or 33 Gy. The ear thickness, desquamation, and erythema combined in an inflammation score were measured for 180 days. The cytokines TGF-β1, TNF-α, IL1α, and IL1β were analyzed in the blood sampled in the first 4 weeks and at termination day. No differences in inflammation reactions were visible in the 23 Gy group for the different dose rates. In the 33 Gy group, the ear swelling and the inflammation score for Flash9 was reduced by (57 ± 12) % and (67 ± 17) % and for Flash930 by (40 ± 13) % and (50 ± 17) % compared to the Conv dose rate. No changes in the cytokines in the blood could be measured. However, an estimation of the irradiated blood volume demonstrates, that 100-times more blood is irradiated when using Conv compared to using Flash9 or Flash930. This indicates that blood might play a role in the underlying mechanisms in the protective effect of FLASH.

## Introduction

Radiotherapy plays the third most important role in the treatment of cancer after surgery and chemotherapy, as during their treatment, about half of cancer patients receive radiotherapy^1^. Here, the goal of radiotherapy is to reach tumor control while minimizing side effects. This goal is often difficult to achieve if, for example, the tumor is hypoxic and therefore less sensitive to radiation. For these cases, higher doses would be necessary to control the tumor. However, increasing the dose leads to more adverse effects. In the past few decades radiotherapy has improved substantially due to to new developments in engineering and computing as well as advances in cancer treatment^2^. Consequently, more cancer patients survive their treatments with long-term side effects of radiation becoming increasingly important^3^. Therefore, we need new solutions to improve the treatment of cancer patients via radiotherapy.

A method to minimize the volume and dose of irradiated healthy tissue and thereby reducing side effects is proton radiotherapy. Unlike the conventionally used photons, protons deposit most of their energy close to the end of their range (known as Bragg peak) in the target volume while depositing only a reduced dose in front of and no dose behind it. This technique improves radiotherapy treatment plans by reducing the dose to organs at risk. Despite this improvement, the skin, which is very sensitive to radiation, is still irradiated with a relevant percentage of the planned tumor dose where the beam enters the body. So, side effects in the skin like fibrosis, permanent hair loss, and depigmentation can be observed in patients leading to tissue stiffness, pain, and discomfort^4,5^.

In the last years, the usage of ultra-high dose rates, designated as FLASH radiotherapy, has been intensively investigated as an approach for decreasing the radiation effects on healthy tissue while maintaining tumor control^6–12^. Most commonly this effect can be seen for dose rates larger than 40 Gy/s. Vozenin et al.^13^ showed that FLASH irradiation of the skin of a mini-pig with 22 Gy to 34 Gy of 6 MeV electrons with an average dose rate of 300 Gy/s led to a significant reduction of acute and late side effects than with conventional irradiation at a dose rate of 0.08 Gy/s. While conventional irradiation led to the permanent destruction of hair follicles and severe late skin fibro-necrosis with 34 Gy, the hair follicles were preserved after FLASH irradiation and histological analysis revealed comparable results to the unirradiated skin. Reduced skin toxicity of FLASH treatment could also be shown using protons. Cunningham et al.^14^ proved that skin toxicity and leg contracture in mice are reduced after FLASH treatment with a dose of 35 Gy and a dose rate of 60 Gy/s and 115 Gy/s compared to a conventional dose rate of 1 Gy/s in an irradiation-induced leg contracture skin model. Although these exemplary results show the great potential of FLASH radiotherapy to decrease side effects in healthy tissue, the underlying mechanisms are still unclear. Among others, the oxygen consumption hypothesis has been suggested as an explanation for the observed tissue-sparing effect^15,16^. During irradiation with ultra-high dose rates, the radiochemical oxygen is depleted. This leads to an acute period of hypoxia in the irradiated tissue and therefore to transient radiation resistance. However, further research has placed this hypothesis under question. For example, Jansen et al. show that it is unlikely to consume enough oxygen by FLASH irradiation to induce significant hypoxia at radiotherapy-relevant doses^17^. Furthermore, it seems that less oxygen is consumed during FLASH irradiation than in conventional irradiation^18^. There are also suggestions that the FLASH effect is ROS-mediated^15,16^, but this has not yet been confirmed. What brings all of these hypotheses together is the dependence of the FLASH effect on oxygen concentration and dose^18,19^. Therefore, oxygen seems to play an important role in tissue sparing during FLASH irradiation. Tissue oxygenation *in-vivo* is mediated by the blood flow, which also transports immune cells and is an important mediator of immune response *in-vivo*. Due to the much shorter irradiation time during FLASH therapy, less amount of blood is irradiated compared to conventional dose rates. Subsequently, fewer circulating immune cells are exposed to radiation^20^. This corresponds well with the finding that FLASH irradiation induced less pro-inflammatory cytokines^10^ and improves T cell infiltration in irradiated tumors^21^, concluding that the immune system is part of the FLASH effect. As a consequence, the irradiated blood volume might be a candidate for playing an important role in the mechanisms of the FLASH effect *in-vivo*.

In this study, we investigated the protective effect of FLASH in a mouse ear model irradiated with 20 MeV protons. The protective effect of FLASH refers, whereby, to the tissue sparing effect of FLASH. The mouse ear model has the advantage that the irradiated blood volume can be easily estimated and compared to visible inflammation reactions, as used in previous studies^22–26^.

## Methods

### Irradiation

Irradiation was performed at the Maier-Leibnitz-Laboratorium in Garching near Munich. The protons were accelerated by a 14 MV tandem accelerator to 20.5 MeV with an LET of 2.6 keV/µm. For irradiation, the SNAKE beamline was used, which is equipped to perform biological experiments with cells, tissues, and animals^26–28^. The proton beam was focused to a homogenous broad beam irradiation field with a field size of 6.5 mm × 6.5 mm. The detailed setup of the beamline is discussed in the results “A proton beamline for FLASH experiments at a tandem accelerator” section.

### Dosimetry

The dose was measured with a specially designed ionization chamber, which is described in the results section in detail. Additionally, for dose verification, the dose was measured with radiochromic films (Ashland Advanced Materials, USA) behind the mouse ears. First, the detector was cross-calibrated with EBT3 Gafchromic films (Ashland Advanced Materials, USA), to the well-established single ion counting system used at SNAKE with a low-dose rate^27^ according to Reinhardt et al.^29^. For dose verification in the FLASH experiment, EBT-XD films need to be used instead of EBT3 films to achieve high accuracy for a wide range of dose rates at doses between 10 and 40 Gy. Therefore, EBT-XD films were calibrated using the calibrated ionization chamber according to^29^ without mice and they were used for dose verification in the experiment. The film calibrations were performed on the same beamline as the experiment and with a dose rate of 9.3 Gy/s. Both film types were scanned with a photo scanner (Perfection V700 Photo, Epson, Japan) 48 h after irradiation. The uncertainty of the dose values as obtained from the EBT-XD film in the range of 20 Gy to 35 Gy is ± 1 Gy and originates from uncertainties of the calibration procedure. The dose error given by the manufacturer is 1%. Together with the uncertainty of the fitting process, this adds up to a dose uncertainty of ± 1 Gy. The doses irradiated to every mouse are listed in Supplementary Table S1.

### Mouse ear model

To investigate the effects of highly different dose rates on normal tissue a tumor-free mouse ear model was chosen. In this study, the right ears of 63 female Balb/c mice were irradiated with 0 Gy, 23 Gy, or 33 Gy at three different dose rates: 0.06 Gy/s, 9.3 Gy/s, and 930 Gy/s. At the time of irradiation, the mice were 12 weeks old. For irradiation, the mice were placed in a specially designed holder that kept the right ear of each mouse in the required position as described in Girst et al.^26^. The central part of the ear was irradiated with the rear of the ear facing the beam.

During irradiation, the mice were kept under general anesthesia, which was induced by intraperitoneal injection of medetomidine (0.5 mg/kg), midazolam (0.5 mg/kg), and fentanyl (0.05 mg/kg). The antagonist atipamezole (2.5 mg/kg), flumazenil (0.5 mg/kg), and naloxone (1.2 mg/kg) were administered subcutaneously after irradiation.

The mice were monitored for 180 days follow-up period. During this period, the monitoring was performed every second to seventh day according to the skin reaction. The monitoring includes a measurement of the ear thickness, scoring of the inflammation reaction, and weekly blood sampling. The number of animals per group is presented in Table 1. In each group, it was aimed to irradiate 8 mice. Due to the two-step dosimetry, it was found after irradiation that some mice had received too little or too much dose and were shifted to another group, resulting in either 7 or 9 animals per group, except for the Flash9 33 Gy group, which contained only 3 mice. Mice in this group did not tolerate the anesthesia and had to be euthanized.

**Table 1:** The number of mice in each group.

Dose [Gy]	Dose rate [Gy/s]	Mice
23	0.06	7
33	0.06	7
23	9.3	9
33	9.3	3
23	930	7
33	930	9
0	Sham	7

#### Ear-thickness measurements

The thickness of both ears of each mouse was measured thrice in the irradiated field using a specially adapted electronic external measuring gauge (C1X079, Kröplin GmbH, Schlüchtern, Germany), with measuring contacts of 6 mm diameter.

#### Skin reaction scoring

Additionally, the inflammation score was measured. This score is composed of the erythema score and the desquamation score. Both are inflammation reactions of the skin after irradiation and can be quantified by visual inspection. The different levels of both reactions and the related scores are listed in Table 2 and were quantified by the four-eyes principle. Both scores were summed up for each scored ear separately resulting in the inflammation score following previous studies^22–26^. Measurements of ear thickness, erythema, and desquamation can be found in Supplementary Table S2. In Fig. S3, photographs of the irradiated mouse ears are presented for one mouse per group at different time points.

**Table 2:** Here, the different gradations of the two inflammation reactions erythema and desquamation are listed with their related scores.

Erythema	Score	Desquamation	Score
No	0	No	0
Mild	0.5	Dry	1
Definite	1.5	Crust formation	2
Severe	3	Moist	3

For the inflammation score, the scores of the erythema and desquamation of each ear were summed.

### Monitoring of inflammation markers in the blood

The blood of every mouse was sampled once a week for the first 4 weeks after irradiation. Before the sampling, the mouse was immobilized in a rack and warmed for 10–15 min with an infrared lamp. The blood was taken from the tail vein with a 25 G needle (BD Microplane™ 3, Nr. 18). On termination day 180 days after irradiation, blood was collected via cardiac puncture while the mice were under deep terminal anesthesia. Afterwards, mice were euthanized by cervical dislocation. From every blood sampling, between 5 to 6 drops of blood were collected into a tube filled with 3.8 ml Heparin. Prior to collection the Heparin (Heparin-Natrium-25000-ratiopharm 5000 IE/ml, ratiopharm GmbH) was diluted to 1000 IE/ml with PBS without Ca^2+^/Mg^2+^ in sterile conditions. The tube with heparin and blood was put on ice until it was centrifuged for 10 min at 2000 rcf (relative centrifugal force) at 4 °C. The supernatant was transferred to an 1.5 ml Eppendorf tube. The cell pellet was resolved in 100 µl NaCl and transferred to an RNAprotect Animal Blood Tube (Qiagen, Germany). The supernatant and the resolved cell pellet were then stored at − 80 °C. RNA was extracted from the mouse blood samples using RNeasy Protect Animal Blood Kit (Qiagen, Germany). The concentration of RNA was measured by a Nanodrop™ spectrophotometer at 260 nm. RNA was reversely transcribed into cDNA using High Capacity cDNA Reverse Transcription Kits (Invitrogen, Darmstadt, Germany). The resulting cDNA was subjected to quantitative RT-PCR using primers directed towards the following genes TGF-β1, TNF-α, IL1α, and IL1β. The genes B2M and GAPDH were used as a house keeping gene. The reaction mix was prepared according to the standard protocol of the kit. RT-PCR was carried out with a StepOnePlus (Applied Biosystems, San Francisco, USA) with a standard thermal profile. Transcriptional changes (Ct) were calculated with ΔΔCt method and normalized with the house keeping gene following the method from Livak and Schmittgen^30^. The ΔΔCt is the difference in Ct values for the gene of interest and the corresponding housekeeping gene for a given sample. Mean values were obtained from triplicate samples. The fold change (FC) is calculated by

1 $$FC= {2}^{-\Delta \Delta Ct}$$

and equals to the normed difference in number of DNA fragments of the desired length produced between an irradiated group and the sham group. For comparison of gene expression, usually the logarithm with a base of two of the FC ($log2 FC$) is given, as this value is less prone to outliers.

2 $$log2 FC= {{\text{log}}}_{2}(FC)$$

The raw data can be found in Supplementary Table S3.

### Ethical approval

The mice were held in a temperature-regulated animal facility with a 12-h light/dark cycle and had ad libitum access to food and water at the animal facility of the Helmholtz Zentrum München. The cages were enriched with wooden sticks for gnawing, paper homes for retreat and paper strands, which serve as hay-substitute for building nests. The experiment was approved by the District Government of Upper Bavaria—Subject area 55.2—Legal issues health, consumer protection and pharmacy (Az. 55.2.1.54-2532-9-2017) and followed the animal welfare and ethical guidelines of our institutions. All methods were performed in accordance with the relevant guidelines and regulations. This study was reported in accordance with ARRIVE guidelines.

## Results

### A proton beamline for FLASH experiments at a tandem accelerator

**Figure 1:**
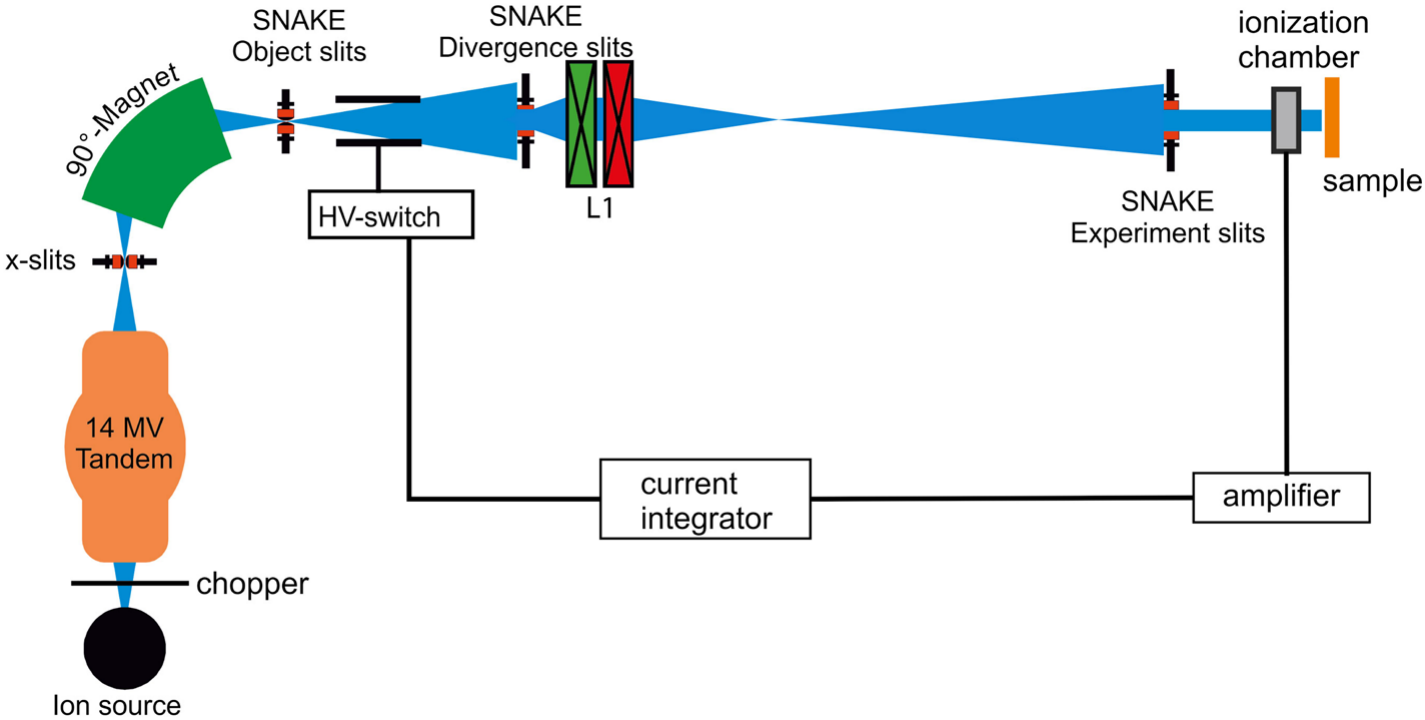
A schematic image of the modified SNAKE beamline used for the FLASH experiments. New in this beamline is the operation of a chopper in front of the accelerator, which makes it possible to switch quickly between different dose rates. Additionally, a set of slits and lenses is used to prepare a homogenous irradiation field of 6.5 mm × 6.5 mm at the sample level.

In this study, the right ears of mice were irradiated with protons using three different mean dose rates: 0.06 Gy/s (Conv), 9.3 Gy/s (Flash9), and 930 Gy/s (Flash930). A dose rate of 0.06 Gy/s was the smallest dose rate that was suitable to form a homogeneous irradiation field and equals to conventionally used dose rates in clinical settings. A dose rate of 930 Gy/s was the highest possible dose rate we could achieve with our setup while being able to create a homogenous irradiation field. A dose rate of 9.3 Gy/s was chosen to test whether a protective effect of FLASH could also be found in a dose rate smaller than the suggested FLASH dose rates (> 40 Gy/s). Doses of either 23 Gy or 33 Gy were applied. Originally, it was aimed for irradiating 25 Gy and 35 Gy. As the dosimetry in our setup was a two-step process, the actual irradiated doses turned out to be on average 23 Gy and 33 Gy. In earlier studies^22,24–26^ on this mouse model, it was found that side effects measurable with the used monitoring methods occur between doses of 20 Gy and 40 Gy. Therefore, we tested two doses at the lower and the upper limit of this dose range. In this study, we present a set-up (cf. the schematic image of the set-up in Fig. 1) with which either conventional dose rates and also FLASH dose rates up to 930 Gy/s can be irradiated. The dose is applied in a continuous beam mode for the highest dose-rate and in a pulsed beam mode for Flash9 and Conv and it was shot through the pinnal tissue. A multicusp ion source is used to yield the required maximum beam current of 15 nA at the sample. A 5 MHz chopper in front of the 14 MV tandem accelerator is used to adjust the dose rates by adjusting the ratio of beam-on and beam-off time. The terminal voltage of the tandem accelerator was set to 10 MV to obtain 20.5 MeV protons for irradiation with an LET of 2.6 keV/µm. The beam is sorted by the 90° magnet to achieve a proton beam with a relative energy spread of smaller $2\cdot {10}^{-4}$. The beam was injected into the 0°-beam line of SNAKE. In the focus level of the 90° magnet, the beam is cut horizontally and vertically by object slits^31^, which define the field size of the object. The next pairs of slits in the beamline are the divergence slits^31^. These restrict the divergence of the beam. For this application, they had an opening of 5 mm in the horizontal direction and 8 mm in the vertical direction to enable a field size of several millimeters. A quadrupole-duplet lens (L1) downstream of the divergence slits focuses the beam to a certain spot in the beam line before the irradiation platform. At the irradiation platform, the beam is then defocused, which results in an irradiation field size of several centimeters. The experiment slits, which are located about 2 m before the irradiation platform, finally restrict the size of the irradiation field to the homogenous core of the beam. The dose homogeneity of the field with a size of 6.5 × 6.5 mm, prepared for this study, was measured with an EBT3 gafchromic film and had a standard deviation of 2% which equals the uncertainty of this film type measured in previous studies^29,32^. To adjust the beam currents and therefore the dose rates, the settings of the low-energy 5 MHz chopper and L1 were adjusted. For Flash930, the low-energy chopper was switched off and the focus of L1 was set to achieve a 15 nA beam current at the target. For Flash9, the 5 MHz chopper was set to pass a pulse width of 128 ns and a frequency partition of 2^6^, resulting in a pulse to pulse distance of 12.8 µs. The focus of L1 was the same as for Flash930. For Conv, the pulse length and the delay of the chopper were the same as in Flash9, but the frequency partition was set to 2^8^, resulting in a pulse to pulse distance of 15.2 µs. The magnetic field in L1 was increased to further decrease the beam current at the target to mean current of ~ 1 pA. The resulting beam-off and beam-on times for every dose rate are listed in Table 3. Configuring the dose rates via the 5 MHz chopper allows a quick change between the mean dose rates within seconds.

**Table 3:** Irradiation parameters in this study. The peak current/peak dose rate is the current or dose rate applied in a pulse for the pulsed beam mode.

Field of irradiation	Peak current	Mean current	Duration for 33 Gy	Peak dose rate	Mean dose rate	Abbreviation
42 mm^2^	15 nA	15 nA	0.035 s	930 Gy/s	930 Gy/s	Flash930
42 mm^2^	15 nA	0.15 nA	3.55 s	930 Gy/s	9.3 Gy/s	Flash9
42 mm^2^	0.4 nA	0,001 nA	9.17 min	24 Gy/s	0.06 Gy/s	Conv

The duration is the time that was needed to irradiate 33 Gy.

### Detector for ultra-high dose rate irradiations

**Figure 2:**
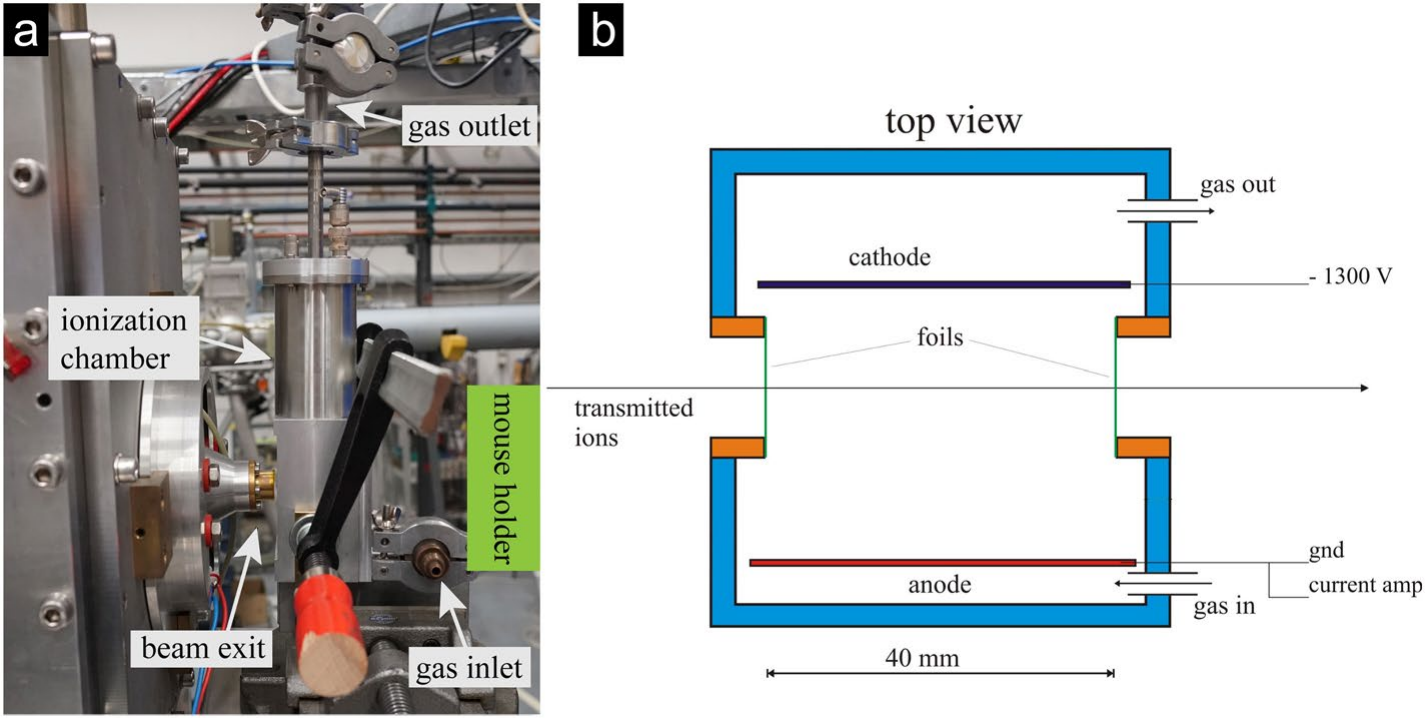
In (**a**) an image of the detector is shown. The detector is located between the beam nozzle and the sample. In (**b**) a schematic image of the detector from the top view is shown. The cathode and anode are aligned parallel to the beam and a voltage of 1300 V is applied. The detector is filled with CF_4_ gas at room pressure and kept under a constant gas flow. Transmitted ions have to pass two 7.5 µm thin Kapton foil windows at the entrance and exit of the detector.

Detecting ultra-high dose rates correctly is crucial for performing experiments on the protective effect of FLASH. This is also a huge problem as conventionally used detector systems integrated into the experimental setups are too slow to detect such high beam currents accurately. For example, the photomultiplier conventionally used at SNAKE^27^ is suited to detect and count single ions but fails for beam currents larger than several Picoampere. In this study, we used beam currents up to 15 nA for irradiation with dose rates up to 930 Gy/s. So, we needed a new and fast detector system. An ionization chamber is a possible candidate for these requirements due to its simple electronics and short reaction time. For this study, we designed and built such a detector adapting a previous design that has been used for ERD (Elastic Recoil Detection) experiments^33^. As shown in Fig. 2a, the detector is placed in front of the beam nozzle. The mice are irradiated behind the detector. The detector itself has two windows (as shown in Fig. 2b as a schematic image) made out of 7.5 µm thin Kapton foil. This foil holds the gas inside the detector and is thin enough to keep the scattering of the proton beam at an acceptable level. The detector has a width of 4 cm between the windows. To ensure the detector can react fast enough, it is filled with CF_4_ gas. The gas is kept under room pressure and a constant gas flow of 10 mbar/min is applied to maintain gas purity and avoid saturation due to damages to the detector gas. Between the cathode and anode, a voltage of -1300 V is applied. The cathode and anode are aligned parallel to the beam such that the electrical field is perpendicular to the beam direction. The proton beam induces ionizations inside the chamber. Free electrons and ions move toward the anode or cathode inducing a current. The current is amplified and integrated. If a certain dose level is reached, which indicates that a certain dose was applied, a high voltage switch (cf. HV-switch in Fig. 1) upstream on the beamline deflects the beam towards the wall and so the irradiation stops immediately. With the presented detector-beamline set-up, the following accuracies in dose were achieved in this study measured using EBT-XD gafchromic films placed behind the mouse ears. For the conventional dose rate, the standard deviation for all irradiations was ± 0.8 Gy for 23 Gy and ± 1.2 Gy for 33 Gy. For the Flash9 dose rate, it was ± 4 Gy for 23 Gy irradiation and ± 2.5 Gy for 33 Gy, and for the Flash930 dose rate, it was ± 5 Gy for 23 Gy and ± 2.7 Gy for 33 Gy. The high standard deviation of the Flash930 dose rate mainly originates from mouse 17 which was irradiated with 16 Gy instead of 23 Gy. Such differences in the dose application occurred due to beam current variations from the accelerator resulting in varying ionization yields from the detector, which could not be fully compensated.

### Measurement of the ear-thickness and skin response scoring

After irradiation, the mice were monitored every second to seventh day depending on the severity of the skin response for 180 days. This monitoring includes on one hand the measurement of the ear thickness with a specially adapted caliper. The ear swelling ${ES}_{i}\left(t\right)$ of mouse $i$ in the irradiated field at the right ear at each time point was determined by the following equation:

3 $${ES}_{i}\left(t\right)= {ET}_{i}\left(t\right)- \overline{{ET }_{sham}\left(t\right)},$$

where ${ET}_{i}\left(t\right)$ is the ear thickness of mouse $i$ at time $t$ and $\overline{{ET }_{sham}(t)}$ the mean ear thickness of all sham mice at this time point. On the other hand, the inflammation reactions of the skin were monitored. Here, the desquamation and the erythema are scored according to Table 2 and both scores were added resulting in the inflammation score. The raw and mouse-specific data can be found in Supplementary Table S2 and are shown in Supplementary Figs. S1 and S2.

**Figure 3:**
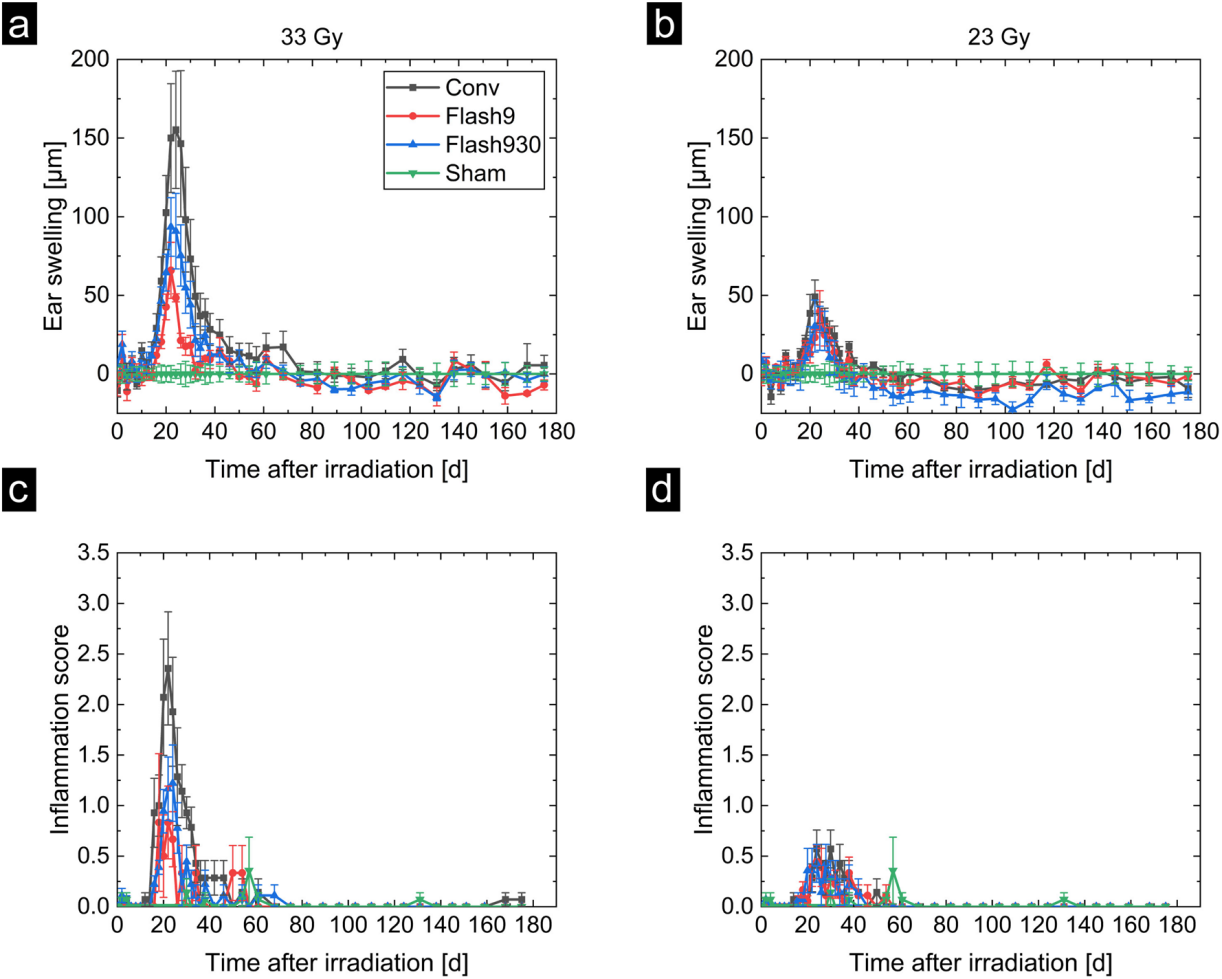
Results for ear swelling and inflammation scores. In (**a**) and (**b**) ear swelling over 180 days is shown. The ear swelling is defined here as the difference between the ear thickness of the right ear of a mouse and the mean ear thickness of the right ears of mice of the sham group. In (**c**) and (**d**) the inflammation score is shown. This score is the sum of the scoring of the erythema and the desquamation reaction of the right ears. (**a**) and (**c**) depict the results of mice irradiated with 33 Gy, and (**b**) and (**d**) of mice irradiated with 23 Gy.

In Fig. 3, the results of the monitoring are shown. Figure 3a and b present the results of the ear swelling, (a) for the group of mice irradiated with a dose of 33 Gy and (b) for the group of mice irradiated with a dose of 23 Gy. For 33 Gy, the ear-swelling after the irradiation starts at day 0 at (0 ± 25) µm (mean ± SEM) and reaches the maximum between day 22 and day 24 after irradiation for all dose rate groups. Maximum swelling with (155 ± 38) µm occurred in the mice irradiated with the Conv dose rate. Ear swelling was reduced by (40 ± 13) % in the Flash930 group and by (57 ± 12) % in the Flash9 group compared with swelling in mice irradiated at the Conv dose rate. These reductions achieved by FLASH dose rates were not statistically significant with p-values of 0.21 (Flash9 compared to Conv, two-sample t-test, Origin 2021b) and 0.16 (Flash930 compared to Conv, two-sample t-test, Origin 2021b), but show a tendency towards a sparing effect. The Flash9 and Flash930 groups provided similar results that overlapped within their uncertainty ranges. However, a trend toward less swelling was observed in the Flash9 group. This group contained only three mice, so no quantitative significant conclusions could be drawn. Ear swelling returns to baseline after 46 days and is maintained until the end of the observation period for all irradiated mice. For 23 Gy irradiation, the maximum reaction also occurred between 22 and 24 days after irradiation. The mice of the Conv group had a mean maximum ear-swelling of (49 ± 11) µm, the Flash9 group of (40 ± 14) µm, and the Flash930 group of (30 ± 17) µm. However, there is no difference observable within the uncertainty range. After 35 days, the ear-swelling of all dose rate groups decreased to the normal level of (0 ± 25) µm and stays at this level until day 180.

In Fig. 3c and d the inflammation scores are shown. These curves show very similar structures to the ear-swelling. In the 33 Gy group (cf. (c)), the inflammation score starts at day 0 with a score of 0.0 ± 0.2 and increases from day 12 to day 22 or 24 to a maximum reaction. The maximum reaction for the Conv group was 2.4 ± 0.6 and it was decreased by (50 ± 17) % in the Flash930 group and by (67 ± 17) % in the Flash9 group compared to the Conv group. The results of Flash9 and Flash930 were different from Conv with respect to the uncertainty ranges but not statistically significantly different with p-values of 0.16 (Flash9 compared to Conv, two-sample t-test, Origin 2021b) and 0.13 (Flash930 compared to Conv, two-sample t-test, Origin 2021b). After 61 days, the score decreases to the starting level and stays there until day 180. In the 23 Gy group in (d), the maximum reaction was visible at 24 days post-irradiation. Similar to the ear swelling at 23 Gy, these scores are not different within their uncertainty ranges. After 46 days the inflammation score was decreased to basal level in all dose rate groups and remained stable until the end of the monitoring period. The peak at 57 days in the sham group was due to mild self-inflicted injury on the ear by mouse 29, probably during extensive self-grooming, which developed a crust (cf. Supplementary Table S2 and Fig. S2).

### No changes visible in blood markers

**Figure 4:**
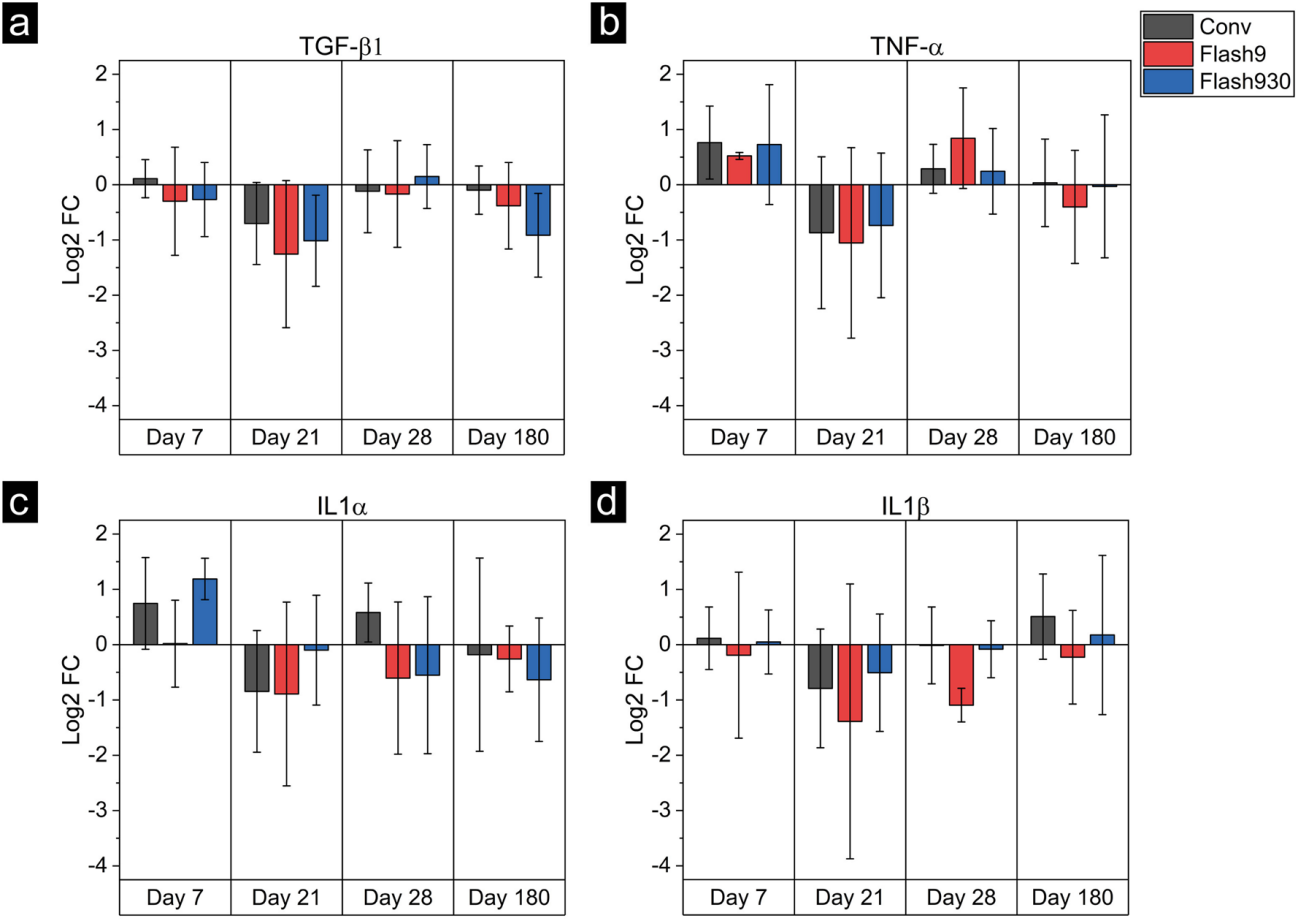
The analysis of four inflammation markers in the blood for the three dose rates. The results of the 2-base logarithm of the fold change (log2 FC) for the Conv irradiation are shown with black bars, for Flash9 with red bars, and for Flash930 with blue bars. The bars show the mean log2 FC and the error bars the standard deviation. The results are shown for days 7, 21, 28, and 180 after irradiation. In (**a**), TGF-β1 is shown, in (**b**) TNF-α, in (**c**) IL1α, and in (**d**) IL1β.

To investigate the effects of different dose rates on the blood, Inflammation markers in the blood, TGF-β1, TNF-α, IL1α, and IL1β were analyzed via qPCR for 33 Gy irradiated mice and sham mice after 7 days, 21 days, 28 days, and 180 days. Figure 4 shows the results of the qPCR of the four markers. Here, the log2 FC (logarithm with base 2 of the fold change) compared to the sham-irradiated mice are depicted. The bars indicate the mean, and the error bars the standard deviation. The fold changes of all four analyzed markers yield the same results for all measured time points and between the applied dose rates. Moreover, the data do not deviate from zero, so no changes due to the inflammatory response induced by irradiation are evident for these cytokines. As no effects of the inflammation reaction due to the irradiation could be found in the blood sampled 7 days, 21 days, 28 days and 180 days after irradiation, the blood sampled 14 days after irradiation was not evaluated.

### Estimation of the irradiated blood volume

**Figure 5:**
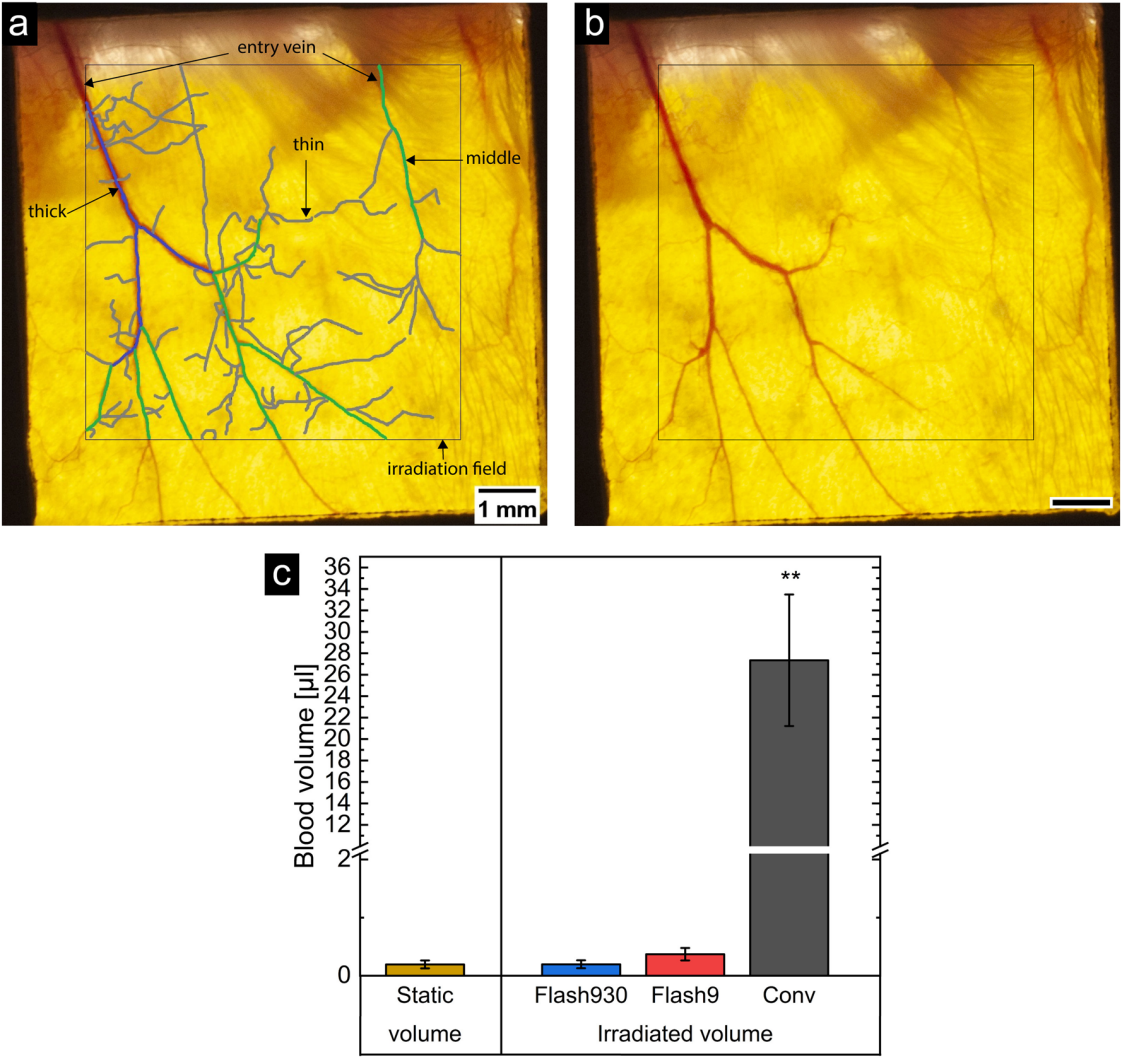
Estimation of irradiated blood volume. In (**a**) and (**b**) a photograph of a mouse ear is shown. The irradiation field is marked as indicated. In (**a**) the blood vessels are marked in three colors according to the evaluation. Blue indicates thick blood vessels, green middle thick blood vessels and gray thin blood vessels. From the length and the diameter, the static volume was determined and by measuring the thickness of the entry veins, the blood flow volume was calculated. In (**b**), the raw image is shown. In (**c**), the results of the estimated irradiated volumes and of the static volume are shown. The irradiated volume was estimated for a dose of 33 Gy for the three irradiation modes (Flash930, Flash9 and Conv) used in this experiment. The error bars show the standard deviation of the mean and two stars indicate p-values < 0.0004 (ANOVA, one-way, Origin 2021b). For a better visualization, the y-axes was interrupted between 2.1 µl and 10 µl with larger spaces between 0 µl and 2.1 µl.

Due to their 2D-like shape, mouse ears are the perfect candidate to estimate the volume of irradiated blood by using trans-illuminated photographs of the ear. The irradiated blood volume can be divided into a static blood volume and a blood flow volume. The static blood volume is defined as the volume of blood that the irradiated area of the ear contains. This volume was estimated by photos of three mouse ears directly after irradiation. To make the blood vessels visible, the ears were illuminated from the other side while the photo was taken. The mice that were used to do blood flow estimation are from a previous experiment, published here^26^, where the mouse strain, the setup, and the handling were exactly the same besides the irradiation. For the approximation on each photo, a 6.5 mm × 6.5 mm field in the middle of the mouse holder window was marked. This is the area which was irradiated in this study. Inside this area, segmented lines along all visible blood vessels were drawn via ImageJ and are depicted in Fig. 5a. In Fig. 5b, the original image is shown. The segments of the lines marking the blood vessels were categorized by eye into three groups according to their thickness and on each photo, the diameters of three or more representative blood vessels for each group were measured at different locations. Thick blood vessels had a diameter of approx. 0.09 mm, middle thick blood vessels of approx. 0.05 mm and thin blood vessels of approx. 0.04 mm. The length of each segment marking a blood vessel was measured and the static volume ${V}_{static}$ for all n blood vessels ($n= {n}_{thick}+{n}_{middle}+{n}_{thin}$) was calculated by

4 $${V}_{static}=\sum_{i=1}^{{n}_{thick}}\pi \cdot \frac{{d}_{thick}^{2}}{4}\cdot {l}_{i}+ \sum_{j=1}^{{n}_{middle}}\pi \cdot \frac{{d}_{middle}^{2}}{4}\cdot {l}_{j}+\sum_{k=1}^{{n}_{thin}}\pi \cdot \frac{{d}_{thin}^{2}}{4}\cdot {l}_{k}$$

assuming a cylindrical form of the blood vessels, where ${d}_{thick}$, ${d}_{middle}$ and ${d}_{thin}$ are the mean diameters for the three categories and ${l}_{i,k,j}$ is the length of each vessel.

The blood flow volume describes the volume of blood which is exchanged during the time of irradiation. This exchange happens only at blood vessels entering the irradiation field from outside. To estimate the amount of blood volume, which is exchanged in a certain time, we calculated how much blood is pumped inside/outside the irradiation field. This was done by estimating the cross-sectional area of the entry blood vessels. These are the blood vessels which deliver the blood from the inner ear towards the irradiation field (see Fig. 5a) or vice versa. For this estimation, only thick and middle-thick blood vessels were taken into account. We assumed that the entry blood vessels are mainly veins as these are bigger than arteries. The diameters of these veins were measured thrice at the entry to the irradiation field. Veins in Balb/c mice have a blood flow speed of v ~ 2 mm/s^34^. So the blood flow volume ${V}_{flow}$ can be calculated by

5 $${V}_{flow}= \sum_{i=1}^{m}\pi \cdot \frac{{d}_{i}^{2}}{4}\cdot v\cdot t$$

where $m$ is the number of entry veins, $d$ their diameter, $v$ the blood flow speed, and $t$ the time required by an irradiation mode for a dose of 33 Gy. The irradiation with Flash930 took ${t}_{Flash930}$ = 0.035 s, with Flash9 ${t}_{Flash9}$ = 3.55 s and with Conv ${t}_{Conv}$ = 9.17 min.

The complete amount of irradiated blood ${V}_{irr}$ was then calculated by

6 $${V}_{irr}={V}_{static}+{V}_{flow}.$$

The irradiated volume is (0.20 ± 0.07) µl in case of Flash930, (0.37 ± 0.11) µl in case of Flash9 and (27 ± 7) µl in case of Conv irradiation modes. This means a fraction of (0.010 ± 0.004) % (Flash930), (0.019 ± 0.006) % (Flash9) and (1.4 ± 0.4) % (Conv) of the total blood volume of about 2 ml. Balb/c mice have 10.35 ml blood per 100 g of body weight^35^. The mice in this study had a mean body weight of (19.2 ± 1.1) g. In Fig. 5c, the static volume and the three irradiated volumes for a 33 Gy irradiation for the different dose rates are shown. As the static volume is independent of time it is the same for all irradiation modes. In contrast, the blood flow volume and thus the irradiated volumes are time-dependent and were estimated for each mode individually. It can be seen that the static volume and the Flash930 irradiated volume result in the same amount of irradiated blood. The Flash9 irradiated volume was about 1.9 times higher than for Flash930 and the static blood volume, but this difference was not significant (p-value = 0.13, Anova, one-way, Origin 2021b). Whereas, the Conv irradiated volume is significantly different (approx. 100 times higher, p-value = 0.0004, Anova, one-way, Origin 2021b). This shows, that for Flash9 and Flash930 the irradiated volume consists mainly of the static volume, while for Conv the blood flow volume mainly determines the amount of the irradiated volume. Therefore, it can be concluded that with Flash930 the irradiated blood volume received the full dose of 33 Gy, with Flash9 17 Gy and with Conv the much larger irradiated blood volume received a dose of only 0.24 Gy.

## Discussion

In this study, we investigated the effects of proton-FLASH on a mouse ear model in BALB/c mice. For the irradiation of different dose rates ranging from conventional dose rates to ultra-high dose rates up to 930 Gy/s, a new setup was installed at the 14 MV tandem accelerator in the Maier-Leibnitz-Laboratorium in Garching near Munich and is presented in this study. The dosimetry was performed with an ionization chamber specially designed for measuring a broad range of dose rates. On this set-up, the ears were successfully irradiated with 20 MeV protons homogeneously distributed in a field of 6.5 mm × 6.5 mm with either a dose rate of 0.06 Gy/s (Conv), 9.3 Gy/s (Flash9), or 930 Gy/s (Flash930). Either a mean dose of 23 Gy or 33 Gy was applied to each group of mice. As a direct control, one group of mice was sham irradiated.

The mouse ear model is a well-tested model and previous experiments showed its potential as a model for investigating side effects of radiotherapy treatment in skin tissue^22–26^. The advantages of this model are that mouse ears are thin enough to shoot through with lower energy protons (here 20.5 MeV) as well as that the blood vessels can be visualized on a trans-illuminated photograph and, consequently, the irradiated blood volume can be easily estimated. The skin is irradiated in almost every treatment scenario. Irradiation damages on the skin lead to stiffness, discomfort, and pain^4,5^. Therefore, it is an important target tissue to test the healthy-tissue-sparing effect of FLASH. The monitoring in this study lasted for 180 days, enabling long-term reactions to be tested.

The results of ear thickness and inflammation showed a sparing effect for Flash930 and Flash9. This sparing effect occurs only for the dose of 33 Gy and not for that of 23 Gy. For 23 Gy, the ear swelling and the inflammation score yield the same results for all dose rate groups, with a slightly increased ear swelling and inflammation score compared to the sham group. This dose induced a very low inflammation reaction in this assay and therefore this assay with a dose of 23 Gy is not sensitive enough for testing dose rate effects. For 33 Gy, the ear swelling was reduced by (40 ± 13) % for Flash930 and by (57 ± 12) % for Flash9 compared to Conv at the maximum reaction. The inflammation score, consisting of scoring erythema and desquamation, was reduced by (50 ± 17) % for Flash930 and by (68 ± 17) % for Flash9 compared to Conv at maximum reaction. After 46 days, both ear swelling and inflammation score decreased to basal level. The differences between Flash930 and Flash9 for ear swelling and inflammation were not significant. It must also be mentioned here that only three mice were irradiated with 33 Gy at the Flash9 dose rate. The two-step dosimetry used for this study showed that the irradiated doses differ within each group with a standard deviation of 2.5 Gy to 5 Gy in the Flash9 and Flash930 groups. These differences in the received doses might be the reason why the observed sparing was not statistically significant. However, these results show a trend towards a protective effect of FLASH for such a low dose rate as 9.3 Gy/s, which gives a sparing effect with greater or at least equal magnitude than the ultra-high dose rate of 930 Gy/s. A protective effect of FLASH in the skin was also observed before^11,13,14^ and is in good agreement with our results. Vozenin et al.^13^ showed that Conv irradiation with 34 Gy led to permanent destruction of hair follicles and severe late skin fibronecrosis, while the FLASH irradiated skin revealed comparable results to the unirradiated skin. Soto et al.^11^ found that FLASH irradiation results in both a lower incidence and a lower severity of skin ulceration after a single fraction of hemithoracic irradiation of mice at high doses (30 Gy and 40 Gy). Cunningham et al.^14^ investigated the protective effect of FLASH with proton pencil beam scanning on a mouse leg contracture assay with 35 Gy. They found that the leg contracture was diminished by approx. 50% for FLASH irradiation with both 58 Gy/s and 115 Gy/s compared to conventional irradiation. These studies showed similar to our findings that the protective effect of FLASH is dose-dependent and occurs at doses as high as 30 Gy^11,13,14^. Furthermore, the protective effect of FLASH seems to plateau in skin tissue at dose rates between 9.3 Gy/s and 930 Gy/s, as in^14^.

The inflammation markers TGF-β1, TNF-α, IL1α, and IL1β were analyzed for 33 Gy irradiated mice. No differences were seen between the sham mice and the irradiated mice and also between the three dose rate groups. Cunningham et al.^14^ measured 12 different cytokines 12 weeks post-irradiation, among them IL1β and TNF-α. In this study, no differences between irradiated and sham-irradiated animals and between the dose rate groups were observed for IL1β and TNF-α, which matches our data. Here, other cytokines might be better choices for measuring inflammation reactions after irradiation in the blood like Cxcl-1, G-CSF, and GM-CSF^14^. In the same study, also TGF-β1 was measured by ELISA one and four days after irradiation. Here, the Conv irradiated mice had a significantly increased level of TGF-β1 compared to sham mice and FLASH irradiated mice. However in this study, a much larger proportion of the mouse body was irradiated than in the study presented here. Therefore, also a higher blood volume was irradiated which might lead to a measurable increase of TGF-β1. By contrast in our study, only a blood volume of 0.098% to 1.4% of the total blood volume of a mouse was irradiated. Thus, these results suggest that sufficient blood volume must be irradiated to detect a difference in the amount of TGF-β1 in the blood.

The mouse ear model enables the possibility to estimate the irradiated blood volume. The veins can be easily made visible by trans-illumination. The blood flow, along with oxygen saturation and hemoglobin concentration can be measured in the mice ears via single-shot photoacoustic microscopy and are well characterized^34^. In this study, the estimation yields a 100-times higher irradiated blood volume for Conv irradiated mice than for Flash9 or Flash930 irradiated mice. The irradiated blood volume of Flash9 and Flash930 mice was similar to the static blood volume, with Flash9 slightly but not significantly increased by a factor of 1.9. In contrast, the irradiated blood volume of Conv mice was mainly composed of the blood flow volume. Therefore, the irradiation times for Flash930 and Flash9 are in a range where not much more than the volume of blood present in the mouse ear is irradiated. The time is too short for an exchange of blood. By contrast for Conv dose rates, the blood is exchanged several times during the irradiation. The Flash930 and Flash9 irradiated blood was present in the irradiated field during the whole irradiation or at least half of the irradiation and received, therefore, lethal doses of 17 Gy to 33 Gy. At these high doses in a very limited volume of blood, especially lymphocytes are damaged lethally and are removed by apoptosis and necrosis^36^. For Conv, the blood is exchanged several times during the irradiation and, therefore, it receives an average dose of 0.24 Gy. This dose is sublethal and leads to damages like an increased formation of micronuclei in lymphocytes^37^ in previous studies. More damaged blood cells in a higher blood volume (by 100-fold) are likely to be an additional stressor to the immune system. These factors contribute, therefore, in an extended amount to the inflammation reaction and can explain the difference to the Flash9 or Flash930 irradiated mice. This demonstrates that the irradiated blood volume possibly plays an important role in stimulating a protective effect of FLASH *in-vivo* and should be taken into account in future studies. As the blood is also the key part in the oxygen supply in tissue, its role should be further investigated. Here, measurements of the oxygen saturation and the exact blood flow during and after irradiation in *in-vivo* models such as the mouse ear model can be a reasonable method for getting a better insight into the role of blood in the protective effect of FLASH.

This study showed that a protective effect of proton-FLASH could be found in a mouse ear model for a dose rate of 9.3 Gy/s and 930 Gy/s. However, the results found were not significant and further studies are needed to confirm these results. The irradiated blood volume estimation showed that blood might play an important role in the protective effect of FLASH and more investigations of the blood are needed to understand the mechanisms involved in this effect.

### Supplementary Information

Supplementary Information.

## Data Availability

All data generated or analyzed during this study are included in this published article and its Supplementary Information files.

## References

[1] Delaney GP, Barton MB (2015). Evidence-based estimates of the demand for radiotherapy. Clin. Oncol..

[2] Garibaldi C (2017). Recent advances in radiation oncology. Ecancermedicalscience.

[3] Miller KD (2019). Cancer treatment and survivorship statistics, 2019. CA Cancer J. Clin..

[4] DeCesaris CM (2019). Quantification of acute skin toxicities in patients with breast cancer undergoing adjuvant proton versus photon radiation therapy: A single institutional experience. Int. J. Radiat. Oncol. Biol. Phys..

[5] Majeed, H. & Gupta, V. *StatPearls. Adverse Effects Of Radiation Therapy* (2021). 33085406

[6] Favaudon V (2014). Ultrahigh dose-rate FLASH irradiation increases the differential response between normal and tumor tissue in mice. Sci. Transl. Med..

[7] Alaghband Y (2020). Neuroprotection of radiosensitive juvenile mice by ultra-high dose rate FLASH irradiation. Cancers.

[8] Fouillade C (2020). FLASH irradiation spares lung progenitor cells and limits the incidence of radio-induced senescence. Clin. Cancer Res..

[9] Levy K (2020). Abdominal FLASH irradiation reduces radiation-induced gastrointestinal toxicity for the treatment of ovarian cancer in mice. Sci. Rep..

[10] Simmons DA (2019). Reduced cognitive deficits after FLASH irradiation of whole mouse brain are associated with less hippocampal dendritic spine loss and neuroinflammation. Radiother. Oncol..

[11] Soto LA (2020). FLASH irradiation results in reduced severe skin toxicity compared to conventional-dose-rate irradiation. Radiat. Res..

[12] Velalopoulou A (2021). FLASH proton radiotherapy spares normal epithelial and mesenchymal tissues while preserving sarcoma response. Cancer Res..

[13] Vozenin M-C (2019). The advantage of FLASH radiotherapy confirmed in mini-pig and cat-cancer patients. Clin. Cancer Res..

[14] Cunningham S (2021). FLASH proton pencil beam scanning irradiation minimizes radiation-induced leg contracture and skin toxicity in mice. Cancers.

[15] Spitz DR (2019). An integrated physico-chemical approach for explaining the differential impact of FLASH versus conventional dose rate irradiation on cancer and normal tissue responses. Radiother. Oncol..

[16] Abolfath R, Grosshans D, Mohan R (2020). Oxygen depletion in FLASH ultra-high-dose-rate radiotherapy: A molecular dynamics simulation. Med. Phys..

[17] Jansen J (2021). Does FLASH deplete oxygen? Experimental evaluation for photons, protons, and carbon ions. Med. Phys..

[18] Cao X (2021). Quantification of oxygen depletion during FLASH irradiation in vitro and in vivo. Int. J. Radiat. Oncol. Biol. Phys..

[19] Tinganelli W (2022). Ultra-high dose rate (FLASH) carbon ion irradiation: Dosimetry and first cell experiments. Int. J. Radiat. Oncol. Biol. Phys..

[20] Durante M (1999). Measurements of the equivalent whole-body dose during radiation therapy by cytogenetic methods. Phys. Med. Biol..

[21] Rama N (2019). Improved tumor control through T-cell infiltration modulated by ultra-high dose rate proton FLASH using a clinical pencil beam scanning proton system. Int. J. Radiat. Oncol. Biol. Phys..

[22] Sammer M (2021). Normal tissue response of combined temporal and spatial fractionation in proton minibeam radiation therapy. Int. J. Radiat. Oncol. Biol. Phys..

[23] Dombrowsky AC (2019). Acute skin damage and late radiation-induced fibrosis and inflammation in murine ears after high-dose irradiation. Cancers.

[24] Sammer M (2019). Beam size limit for pencil minibeam radiotherapy determined from side effects in an in-vivo mouse ear model. PLoS ONE.

[25] Sammer M (2019). Proton pencil minibeam irradiation of an in-vivo mouse ear model spares healthy tissue dependent on beam size. PLoS ONE.

[26] Girst S (2016). Proton minibeam radiation therapy reduces side effects in an in vivo mouse ear model. Int. J. Radiat. Oncol. Biol. Phys..

[27] Hable V (2009). The live cell irradiation and observation setup at SNAKE. Nucl. Instrum. Methods Phys. Res. Sect. B Beam Interact. Mater. Atoms.

[28] Scherthan H (2022). Planar proton minibeam irradiation elicits spatially confined DNA damage in a human epidermis model. Cancers.

[29] Reinhardt S (2015). Investigation of EBT2 and EBT3 films for proton dosimetry in the 4–20 MeV energy range. Radiat. Environ. Biophys..

[30] Livak KJ, Schmittgen TD (2001). Analysis of relative gene expression data using real-time quantitative PCR and the 2(−Delta Delta C(T)) Method. Methods.

[31] Vallentin T (2015). A microbeam slit system for high beam currents. Nucl. Instrum. Methods Phys. Res. Sect. B Beam Interact. Mater. Atoms.

[32] Moylan R, Aland T, Kairn T (2013). Dosimetric accuracy of Gafchromic EBT2 and EBT3 film for in vivo dosimetry. Austral. Phys. Eng. Sci. Med..

[33] Bergmaier A, Dollinger G, Frey CM (1998). A compact ΔE-Eres detector for elastic recoil detection with high sensitivity. Nucl. Instrum. Methods Phys. Res. Sect. B Beam Interact. Mater. Atoms.

[34] Liu C, Liang Y, Wang L (2020). Single-shot photoacoustic microscopy of hemoglobin concentration, oxygen saturation, and blood flow in sub-microseconds. Photoacoustics.

[35] Vácha J (1975). Blood volume in inbred strain BALB/c, CBA/J and C57BL/10 mice determined by means of 59Fe-labelled red cells and 59Fe bound to transferrin. Physiologia Bohemoslovaca.

[36] Masterson ME, Febo R (1992). Pretransfusion blood irradiation: clinical rationale and dosimetric considerations. Med. Phys..

[37] Fenech MF, Dunaiski V, Osborne Y, Morley AA (1991). The cytokinesis-block micronucleus assay as a biological dosimeter in spleen and peripheral blood lymphocytes of the mouse following acute whole-body irradiation. Mutat. Res..

