# Supplementary material for: Proton-FLASH: effects of ultra-high dose rate irradiation on an in-vivo mouse ear model
Source: Sci Rep. 2024 Jan 16;14:1418. doi: 10.1038/s41598-024-51951-6 (PMC10791610; doi:10.1038/s41598-024-51951-6)
Supplement: Supplementary file 1 — Supplementary Information. [file 41598_2024_51951_MOESM1_ESM.pdf]

Supplementary:

Table S1: Here, for each mouse the dose is listed, with which every mouse was irradiated and the irradiation group. The irradiated doses were measured with radio-gafchromic films. From the irradiated doses of each group the mean and standard deviation were calculated and depicted.

Mouse ID	Group	Dose [Gy]	Mean dose [Gy]	Stdev dose [Gy]
**18**	Conv 23 Gy	23.4	23.4	0.8
**22**	Conv 23 Gy	23.9		
**32**	Conv 23 Gy	23.8		
**36**	Conv 23 Gy	22.1		
**46**	Conv 23 Gy	22.5		
**50**	Conv 23 Gy	24.7		
**60**	Conv 23 Gy	23.6		
**8**	Conv 33 Gy	32.5	32.3	1.2
**26**	Conv 33 Gy	33.1		
**34**	Conv 33 Gy	32.8		
**40**	Conv 33 Gy	31.7		
**48**	Conv 33 Gy	34.4		
**54**	Conv 33 Gy	30.9		
**62**	Conv 33 Gy	30.7		
**31**	Flash9 23 Gy	22.8	23.2	3.5
**38**	Flash9 23 Gy	25.2		
**39**	Flash9 23 Gy	26.0		
**43**	Flash9 23 Gy	21.4		
**44**	Flash9 23 Gy	28.1		
**52**	Flash9 23 Gy	18.3		
**53**	Flash9 23 Gy	23.1		
**56**	Flash9 23 Gy	26.5		
**57**	Flash9 23 Gy	17.1		
**1**	Flash9 33 Gy	36.2	32.9	2.5
**10**	Flash9 33 Gy	32.5		
**58**	Flash9 33 Gy	30.1		
**5**	Flash930 23 Gy	28.6	24.5	4.6
**7**	Flash930 23 Gy	19.0		
**13**	Flash930 23 Gy	24.7		
**17**	Flash930 23 Gy	16.2		
**35**	Flash930 23 Gy	28.3		
**37**	Flash930 23 Gy	28.8		
**55**	Flash930 23 Gy	25.9		
**9**	Flash930 33 Gy	35.6	32.7	2.7
**19**	Flash930 33 Gy	36.5		
**21**	Flash930 33 Gy	29.0		

**23**	Flash930 33 Gy	32.3		
**41**	Flash930 33 Gy	35.9		
**47**	Flash930 33 Gy	31.0		
**49**	Flash930 33 Gy	29.1		
**61**	Flash930 33 Gy	30.7		
**63**	Flash930 33 Gy	34.0		
**15**	sham	0.0	0.0	0.0
**24**	sham	0.0		
**25**	sham	0.0		
**28**	sham	0.0		
**29**	sham	0.0		
**45**	sham	0.0		
**59**	sham	0.0		

Table S2: This table includes all measurements of the inflammation score as well as erythema and desquamation score for each mouse. Additional, the measurements for ear swelling for each mouse and finally the raw measurements of the ear thickness are listed. The ear thickness was measured trice for each time point. The data are listed for every dose rate and sham group.

**Inflammation score**	**Conventional dose rate 23 Gy**										
**time after irradiation [d]**	Mouse ID: 18	22	32	36	46	50	60			mean	SEM
**0**	0	0	0	0	0	0	0			0.00	0.00
**2**	0	0	0	0	0	0	0			0.00	0.00
**4**	0	0	0	0	0	0	0			0.00	0.00
**6**	0	0	0	0	0	0	0			0.00	0.00
**8**	0	0	0	0	0	0	0			0.00	0.00
**10**	0	0	0	0	0	0	0			0.00	0.00
**12**	0	0	0	0	0	0	0			0.00	0.00
**14**	0.5	0	0	0	0	0	0			0.07	0.07
**16**	0	0	0	0	0	0	0.5			0.07	0.07
**18**	0	0	0	0	0	0	0.5			0.07	0.07
**20**	0	0	0	0	0	0	0			0.00	0.00
**22**	0.5	0	1	0.5	0	0	0			0.29	0.14
**24**	1	1	0	0	0	1	1			0.57	0.19
**26**	1	1	0	1	0	0	0			0.43	0.19
**28**	0	1	0	1	0	0	0			0.29	0.17
**30**	0	0	1	1	1	0	1			0.57	0.19
**32**	0	1	1	0	0	0	0			0.29	0.17
**34**	0	1	1	0	0	1	0			0.43	0.19
**36**	1	0	1	0	0	0	0			0.29	0.17
**38**	0	1	1	0	0	0	0			0.29	0.17
**42**	0	0	0	1	0	0	0			0.14	0.13
**46**	0	0	0	0	0	0	0			0.00	0.00
**50**	0	1	0	0	0	0	0			0.14	0.13

**54**	0	0	0	0	0	0	0			0.00	0.00
**57**	0	0	0	0	0	0	0			0.00	0.00
**61**	0	0	0	0	0	0	0			0.00	0.00
**68**	0	0	0	0	0	0	0			0.00	0.00
**75**	0	0	0	0	0	0	0			0.00	0.00
**82**	0	0	0	0	0	0	0			0.00	0.00
**89**	0	0	0	0	0	0	0			0.00	0.00
**96**	0	0	0	0	0	0	0			0.00	0.00
**103**	0	0	0	0	0	0	0			0.00	0.00
**110**	0	0	0	0	0	0	0			0.00	0.00
**117**	0	0	0	0	0	0	0			0.00	0.00
**124**	0	0	0	0	0	0	0			0.00	0.00
**131**	0	0	0	0	0	0	0			0.00	0.00
**138**	0	0	0	0	0	0	0			0.00	0.00
**145**	0	0	0	0	0	0	0			0.00	0.00
**151**	0	0	0	0	0	0	0			0.00	0.00
**159**	0	0	0	0	0	0	0			0.00	0.00
**168**	0	0	0	0	0	0	0			0.00	0.00
**175**	0	0	0	0	0	0	0			0.00	0.00
**Inflammation score**	**Flash9 dose rate 23 Gy**										
**time after irradiation [d]**	Mouse ID: 31	38	39	43	44	52	53	56	57	mean	SEM
**0**	0	0	0	0	0	0	0	0	0	0.00	0.00
**2**	0	0	0	0	0	0	0	0	0	0.00	0.00
**4**	0	0	0	0	0	0	0	0	0	0.00	0.00
**6**	0	0	0	0	0	0	0	0	0	0.00	0.00
**8**	0	0	0	0	0	0	0	0	0	0.00	0.00
**10**	0	0	0	0	0	0	0	0	0	0.00	0.00
**12**	0	0	0	0	0	0	0	0	0	0.00	0.00
**14**	0	0	0	0	0	0	0	0	0	0.00	0.00
**16**	0.5	0	0	0	0	0	0	0	0	0.06	0.05
**18**	0.5	0.5	0	0	0.5	0	0	0	0	0.17	0.08
**20**	0.5	0	0	0	0	0	0	0	0	0.06	0.05
**22**	1.5	0	1	1	0	0	0	0	0	0.39	0.19
**24**	0	0	0	0	0	1	1	1	1	0.44	0.17
**26**	1.5	1	0	0	0	0	1	0	0	0.39	0.19
**28**	0	0	0	0	1	0	0	0	0	0.11	0.10
**30**	1.5	0	0	0	1	0	0	0	0	0.28	0.18
**32**	1	0	0	0	1	0	0	0	0	0.22	0.14
**34**	0	0	0	0	1	0	0	0	0	0.11	0.10
**36**	0.5	0	0	0	0	0	0	0	0	0.06	0.05
**38**	0	0	0	1	1	0	0	0	1	0.33	0.16
**42**	0	0	0	0	1	0	0	0	0	0.11	0.10
**46**	0	0	0	0	0	0	1	0	0	0.11	0.10

**50**	0	0	0	0	0	0	0	0	0	0.00	0.00
**54**	0	0	0	0	0	0	0	1	0	0.11	0.10
**57**	0	0	0	0	0	0	0	0	0	0.00	0.00
**61**	0	0	0	0	0	0	0	0	0	0.00	0.00
**68**	0	0	0	0	0	0	0	0	0	0.00	0.00
**75**	0	0	0	0	0	0	0	0	0	0.00	0.00
**82**	0	0	0	0	0	0	0	0	0	0.00	0.00
**89**	0	0	0	0	0	0	0	0	0	0.00	0.00
**96**	0	0	0	0	0	0	0	0	0	0.00	0.00
**103**	0	0	0	0	0	0	0	0	0	0.00	0.00
**110**	0	0	0	0	0	0	0	0	0	0.00	0.00
**117**	0	0	0	0	0	0	0	0	0	0.00	0.00
**124**	0	0	0	0	0	0	0	0	0	0.00	0.00
**131**	0	0	0	0	0	0	0	0	0	0.00	0.00
**138**	0	0	0	0	0	0	0	0	0	0.00	0.00
**145**	0	0	0	0	0	0	0	0	0	0.00	0.00
**151**	0	0	0	0	0	0	0	0	0	0.00	0.00
**159**	0	0	0	0	0	0	0	0	0	0.00	0.00
**168**	0	0	0	0	0	0	0	0	0	0.00	0.00
**175**	0	0	0	0	0	0	0	0	0	0.00	0.00
**Inflammation score**	**Flash930 dose rate 23 Gy**										
**time after irradiation [d]**	Mouse ID: 5	7	13	17	35	37	55			mean	SEM
**0**	0	0	0	0	0	0	0			0.00	0.00
**2**	0.5	0	0	0	0	0	0			0.07	0.07
**4**	0	0	0	0	0	0	0			0.00	0.00
**6**	0	0	0	0	0	0	0			0.00	0.00
**8**	0	0	0	0	0	0	0			0.00	0.00
**10**	0	0	0	0	0	0	0			0.00	0.00
**12**	0	0	0	0	0	0	0			0.00	0.00
**14**	0	0	0	0	0	0	0			0.00	0.00
**16**	0	0	0	0	0.5	0	0			0.07	0.07
**18**	0.5	0	0	0	0	0	0			0.07	0.07
**20**	0	0	1	0	1.5	0	0			0.36	0.22
**22**	0	0	1	0	1.5	0	0			0.36	0.22
**24**	0	0	1	1	0	0	1			0.43	0.19
**26**	0	1	0	0	0	0	0			0.14	0.13
**28**	0	0	1	1	0	1	0			0.43	0.19
**30**	0	0	1	0	0	1	0			0.29	0.17
**32**	0	0	1	0	1	0	0			0.29	0.17
**34**	0	0	1	0	0	0	0			0.14	0.13
**36**	0	0	1	0	0	0	0			0.14	0.13
**38**	0	0	0	0	0	1.5	0			0.21	0.20
**42**	0	0	0	0	1	0	0			0.14	0.13

**46**	0	0	0	0	0	0	0			0.00	0.00
**50**	0	0	0	0	0	0	0			0.00	0.00
**54**	0	0	0	0	0	0	0			0.00	0.00
**57**	0	0	0	0	0	0	0			0.00	0.00
**61**	0	0	0	0	0	0	0			0.00	0.00
**68**	0	0	0	0	0	0	0			0.00	0.00
**75**	0	0	0	0	0	0	0			0.00	0.00
**82**	0	0	0	0	0	0	0			0.00	0.00
**89**	0	0	0	0	0	0	0			0.00	0.00
**96**	0	0	0	0	0	0	0			0.00	0.00
**103**	0	0	0	0	0	0	0			0.00	0.00
**110**	0	0	0	0	0	0	0			0.00	0.00
**117**	0	0	0	0	0	0	0			0.00	0.00
**124**	0	0	0	0	0	0	0			0.00	0.00
**131**	0	0	0	0	0	0	0			0.00	0.00
**138**	0	0	0	0	0	0	0			0.00	0.00
**145**	0	0	0	0	0	0	0			0.00	0.00
**151**	0	0	0	0	0	0	0			0.00	0.00
**159**	0	0	0	0	0	0	0			0.00	0.00
**168**	0	0	0	0	0	0	0			0.00	0.00
**175**	0	0	0	0	0	0	0			0.00	0.00
**Inflammation score**	**Conventional dose rate 33 Gy**										
**time after irradiation [d]**	Mouse ID: 8	26	34	40	48	54	62			mean	SEM
**0**	0	0	0	0	0	0	0			0.00	0.00
**2**	0	0	0	0	0	0.5	0			0.07	0.07
**4**	0.5	0	0	0	0	0	0			0.07	0.07
**6**	0	0	0	0	0	0	0			0.00	0.00
**8**	0	0	0	0	0	0	0			0.00	0.00
**10**	0	0	0	0	0	0	0			0.00	0.00
**12**	0.5	0	0	0	0	0	0			0.07	0.07
**14**	0.5	0	0	0	0	0	0			0.07	0.07
**16**	1.5	0	2.5	1	0	0	1.5			0.9	0.3
**18**	1.5	0.5	2.5	1.5	0	0.5	0.5			1.0	0.3
**20**	2.5	0.5	5	2.5	2.5	0	1.5			2.1	0.6
**22**	5	1.5	3.5	2.5	1.5	0	2.5			2.4	0.6
**24**	5	1.5	1.5	2.5	1.5	0	1.5			1.9	0.5
**26**	3.5	0	1.5	2.5	1.5	0	0			1.3	0.5
**28**	2.5	1	1	1.5	1	0	1			1.14	0.26
**30**	1.5	1	1	0	1	1	1			0.93	0.16
**32**	1.5	1	1	0	1	0	1			0.79	0.20
**34**	1	0	0	1	0	0	1			0.43	0.19
**36**	1	0	0	0	0	0	1			0.29	0.17
**38**	1	0	0	0	0	1	0			0.29	0.17

**42**	1	0	1	0	0	0	0			0.29	0.17
**46**	1	0	1	0	0	0	0			0.29	0.17
**50**	0	0	0	0	0	0	0			0.00	0.00
**54**	1	0	0	0	0	0	0			0.14	0.13
**57**	0	0	0	0	0	0	0			0.00	0.00
**61**	1	0	0	0	0	0	0			0.14	0.13
**68**	0	0	0	0	0	0	0			0.00	0.00
**75**	0	0	0	0	0	0	0			0.00	0.00
**82**	0	0	0	0	0	0	0			0.00	0.00
**89**	0	0	0	0	0	0	0			0.00	0.00
**96**	0	0	0	0	0	0	0			0.00	0.00
**103**	0	0	0	0	0	0	0			0.00	0.00
**110**	0	0	0	0	0	0	0			0.00	0.00
**117**	0	0	0	0	0	0	0			0.00	0.00
**124**	0	0	0	0	0	0	0			0.00	0.00
**131**	0	0	0	0	0	0	0			0.00	0.00
**138**	0	0	0	0	0	0	0			0.00	0.00
**145**	0	0	0	0	0	0	0			0.00	0.00
**151**	0	0	0	0	0	0	0			0.00	0.00
**159**	0	0	0	0	0	0	0			0.00	0.00
**168**	0	0	0.5	0	0	0	0			0.07	0.07
**175**	0.5	0	0	0	0	0	0			0.07	0.07
**Inflammation score**	**Flash9 dose rate 33 Gy**										
**time after irradiation [d]**	Mouse ID: 1	10	58							mean	SEM
**0**	0	0	0							0.00	0.00
**2**	0	0	0							0.00	0.00
**4**	0	0	0							0.00	0.00
**6**	0	0	0							0.00	0.00
**8**	0	0	0							0.00	0.00
**10**	0	0	0							0.00	0.00
**12**	0	0	0							0.00	0.00
**14**	0	0	0							0.00	0.00
**16**	0	0	0							0.00	0.00
**18**	2.5	0	0							0.8	0.7
**20**	1.5	0	0							0.5	0.4
**22**	1.5	0	1							0.8	0.4
**24**	1	0	1							0.67	0.27
**26**	0	0	0							0.00	0.00
**28**	0	0	1							0.33	0.27
**30**	0	0	0							0.00	0.00
**32**	0	0	0							0.00	0.00
**34**	0	0	1							0.33	0.27
**36**	0	0	0							0.00	0.00

**38**	0	0	0							0.00	0.00
**42**	0	0	0							0.00	0.00
**46**	0	0	0							0.00	0.00
**50**	1	0	0							0.33	0.27
**54**	1	0	0							0.33	0.27
**57**	0	0	0							0.00	0.00
**61**	0	0	0							0.00	0.00
**68**	0	0	0							0.00	0.00
**75**	0	0	0							0.00	0.00
**82**	0	0	0							0.00	0.00
**89**	0	0	0							0.00	0.00
**96**	0	0	0							0.00	0.00
**103**	0	0	0							0.00	0.00
**110**	0	0	0							0.00	0.00
**117**	0	0	0							0.00	0.00
**124**	0	0	0							0.00	0.00
**131**	0	0	0							0.00	0.00
**138**	0	0	0							0.00	0.00
**145**	0	0	0							0.00	0.00
**151**	0	0	0							0.00	0.00
**159**	0	0	0							0.00	0.00
**168**	0	0	0							0.00	0.00
**175**	0	0	0							0.00	0.00
**Inflammation score**	**Flash930 dose rate 33 Gy**										
**time after irradiation [d]**	Mouse ID: 9	19	21	23	41	47	49	61	63	mean	SEM
**0**	0	0	0	0	0	0	0	0	0	0.00	0.00
**2**	0.5	0	0.5	0	0	0	0	0	0	0.11	0.07
**4**	0.5	0	0	0	0	0	0	0	0	0.06	0.05
**6**	0	0	0	0	0	0	0	0	0	0.00	0.00
**8**	0	0	0	0	0	0	0	0	0	0.00	0.00
**10**	0	0	0	0	0	0	0	0	0	0.00	0.00
**12**	0	0	0	0	0	0	0	0	0	0.00	0.00
**14**	0	0	0	0	0	0	0	0	0	0.00	0.00
**16**	0.5	0	0	0	0	0.5	0	0.5	0.5	0.22	0.08
**18**	0.5	0.5	0.5	0.5	0	0.5	0.5	0	0.5	0.39	0.07
**20**	1.5	1.5	0.5	0.5	0	0	1.5	1.5	1.5	0.94	0.21
**22**	1	2.5	0	1.5	1.5	0	0	1.5	2.5	1.2	0.3
**24**	0	2.5	0	1.5	3.5	0	1	1.5	1	1.2	0.4
**26**	0	2.5	1	1	1	0	0	0.5	1	0.78	0.25
**28**	0	1.5	0	0	0	0	0	0	0	0.17	0.16
**30**	1	1	1	0	0	0	0	0	1	0.44	0.17
**32**	0	1	1	0	0	0	0	0	0	0.22	0.14
**34**	0	0	0	0	0	0	0	0	1	0.11	0.10

**36**	0	0	0	0	0	0	0	0	0	0.00	0.00
**38**	0	0	1	0	0	0	0	1	0	0.22	0.14
**42**	0	0	0	0	0	0	0	0	0	0.00	0.00
**46**	0	0	0	0	1	0	0	0	0	0.11	0.10
**50**	0	0	0	0	0	0	0	0	0	0.00	0.00
**54**	0	0	0	0	1	0	0	0	0	0.11	0.10
**57**	0	0	0	0	0	0	0	0	0	0.00	0.00
**61**	0	1	0	0	0	0	0	0	0	0.11	0.10
**68**	1	0	0	0	0	0	0	0	0	0.11	0.10
**75**	0	0	0	0	0	0	0	0	0	0.00	0.00
**82**	0	0	0	0	0	0	0	0	0	0.00	0.00
**89**	0	0	0	0	0	0	0	0	0	0.00	0.00
**96**	0	0	0	0	0	0	0	0	0	0.00	0.00
**103**	0	0	0	0	0	0	0	0	0	0.00	0.00
**110**	0	0	0	0	0	0	0	0	0	0.00	0.00
**117**	0	0	0	0	0	0	0	0	0	0.00	0.00
**124**	0	0	0	0	0	0	0	0	0	0.00	0.00
**131**	0	0	0	0	0	0	0	0	0	0.00	0.00
**138**	0	0	0	0	0	0	0	0	0	0.00	0.00
**145**	0	0	0	0	0	0	0	0	0	0.00	0.00
**151**	0	0	0	0	0	0	0	0	0	0.00	0.00
**159**	0	0	0	0	0	0	0	0	0	0.00	0.00
**168**	0	0	0	0	0	0	0	0	0	0.00	0.00
**175**	0	0	0	0	0	0	0	0	0	0.00	0.00
**Inflammation score**	**Sham irradiated 0 Gy**										
**time after irradiation [d]**	Mouse ID: 15	24	25	28	29	45	59			mean	SEM
**0**	0	0	0	0	0	0	0			0.00	0.00
**2**	0	0.5	0	0	0	0	0			0.07	0.07
**4**	0	0	0.5	0	0	0	0			0.07	0.07
**6**	0	0	0	0	0	0	0			0.00	0.00
**8**	0	0	0	0	0	0	0			0.00	0.00
**10**	0	0	0	0	0	0	0			0.00	0.00
**12**	0	0	0	0	0	0	0			0.00	0.00
**14**	0	0	0	0	0	0	0			0.00	0.00
**16**	0	0	0	0	0	0	0			0.00	0.00
**18**	0	0	0	0	0	0	0			0.00	0.00
**20**	0	0	0	0	0	0	0			0.00	0.00
**22**	0	0	0	0	0	0	0			0.00	0.00
**24**	0	0	0	0	0	0	0			0.00	0.00
**26**	0	0	0	0	0	0	0			0.00	0.00
**28**	0	0	0	0	0	0	0			0.00	0.00
**30**	0	0	0	0	0	1	0			0.14	0.13
**32**	0	0	0	0	0	0	0			0.00	0.00

**34**	0	0	0	0	0	0	0			0.00	0.00
**36**	0	0	0	0	0	0	0			0.00	0.00
**38**	0	0	0	0.5	0	0	0			0.07	0.07
**42**	0	0	0	0	0	0	0			0.00	0.00
**46**	0	0	0	0	0	0	0			0.00	0.00
**50**	0	0	0	0	0	0	0			0.00	0.00
**54**	0	0	0	0	0.5	0	0			0.07	0.07
**57**	0	0	0	0	2.5	0	0			0.36	0.33
**61**	0	0	0	0	0.5	0	0			0.07	0.07
**68**	0	0	0	0	0	0	0			0.00	0.00
**75**	0	0	0	0	0	0	0			0.00	0.00
**82**	0	0	0	0	0	0	0			0.00	0.00
**89**	0	0	0	0	0	0	0			0.00	0.00
**96**	0	0	0	0	0	0	0			0.00	0.00
**103**	0	0	0	0	0	0	0			0.00	0.00
**110**	0	0	0	0	0	0	0			0.00	0.00
**117**	0	0	0	0	0	0	0			0.00	0.00
**124**	0	0	0	0	0	0	0			0.00	0.00
**131**	0	0	0	0.5	0	0	0			0.07	0.07
**138**	0	0	0	0	0	0	0			0.00	0.00
**145**	0	0	0	0	0	0	0			0.00	0.00
**151**	0	0	0	0	0	0	0			0.00	0.00
**159**	0	0	0	0	0	0	0			0.00	0.00
**168**	0	0	0	0	0	0	0			0.00	0.00
**175**	0	0	0	0	0	0	0			0.00	0.00
**Erythema score**	**Conventional dose rate 23 Gy**										
**time after irradiation [d]**	Mouse ID: 18	22	32	36	46	50	60			mean	SEM
**0**	0	0	0	0	0	0	0			0.00	0.00
**2**	0	0	0	0	0	0	0			0.00	0.00
**4**	0	0	0	0	0	0	0			0.00	0.00
**6**	0	0	0	0	0	0	0			0.00	0.00
**8**	0	0	0	0	0	0	0			0.00	0.00
**10**	0	0	0	0	0	0	0			0.00	0.00
**12**	0	0	0	0	0	0	0			0.00	0.00
**14**	0.5	0	0	0	0	0	0			0.07	0.07
**16**	0	0	0	0	0	0	0.5			0.07	0.07
**18**	0	0	0	0	0	0	0.5			0.07	0.07
**20**	0	0	0	0	0	0	0			0.00	0.00
**22**	0.5	0	0	0.5	0	0	0			0.14	0.09
**24**	0	0	0	0	0	0	0			0.00	0.00
**26**	0	0	0	0	0	0	0			0.00	0.00
**28**	0	0	0	0	0	0	0			0.00	0.00
**30**	0	0	0	0	0	0	0			0.00	0.00

**32**	0	0	0	0	0	0	0			0.00	0.00
**34**	0	0	0	0	0	0	0			0.00	0.00
**36**	0	0	0	0	0	0	0			0.00	0.00
**38**	0	0	0	0	0	0	0			0.00	0.00
**42**	0	0	0	0	0	0	0			0.00	0.00
**46**	0	0	0	0	0	0	0			0.00	0.00
**50**	0	0	0	0	0	0	0			0.00	0.00
**54**	0	0	0	0	0	0	0			0.00	0.00
**57**	0	0	0	0	0	0	0			0.00	0.00
**61**	0	0	0	0	0	0	0			0.00	0.00
**68**	0	0	0	0	0	0	0			0.00	0.00
**75**	0	0	0	0	0	0	0			0.00	0.00
**82**	0	0	0	0	0	0	0			0.00	0.00
**89**	0	0	0	0	0	0	0			0.00	0.00
**96**	0	0	0	0	0	0	0			0.00	0.00
**103**	0	0	0	0	0	0	0			0.00	0.00
**110**	0	0	0	0	0	0	0			0.00	0.00
**117**	0	0	0	0	0	0	0			0.00	0.00
**124**	0	0	0	0	0	0	0			0.00	0.00
**131**	0	0	0	0	0	0	0			0.00	0.00
**138**	0	0	0	0	0	0	0			0.00	0.00
**145**	0	0	0	0	0	0	0			0.00	0.00
**151**	0	0	0	0	0	0	0			0.00	0.00
**159**	0	0	0	0	0	0	0			0.00	0.00
**168**	0	0	0	0	0	0	0			0.00	0.00
**175**	0	0	0	0	0	0	0			0.00	0.00
**Erythema score**	**Flash9 dose rate 23 Gy**										
**time after irradiation [d]**	Mouse ID: 31	38	39	43	44	52	53	56	57	mean	SEM
**0**	0	0	0	0	0	0	0	0	0	0.00	0.00
**2**	0	0	0	0	0	0	0	0	0	0.00	0.00
**4**	0	0	0	0	0	0	0	0	0	0.00	0.00
**6**	0	0	0	0	0	0	0	0	0	0.00	0.00
**8**	0	0	0	0	0	0	0	0	0	0.00	0.00
**10**	0	0	0	0	0	0	0	0	0	0.00	0.00
**12**	0	0	0	0	0	0	0	0	0	0.00	0.00
**14**	0	0	0	0	0	0	0	0	0	0.00	0.00
**16**	0.5	0	0	0	0	0	0	0	0	0.06	0.05
**18**	0.5	0.5	0	0	0.5	0	0	0	0	0.17	0.08
**20**	0.5	0	0	0	0	0	0	0	0	0.06	0.05
**22**	1.5	0	0	0	0	0	0	0	0	0.17	0.16
**24**	0	0	0	0	0	0	0	0	0	0.00	0.00
**26**	0.5	0	0	0	0	0	0	0	0	0.06	0.05
**28**	0	0	0	0	0	0	0	0	0	0.00	0.00

**30**	0.5	0	0	0	0	0	0	0	0	0.06	0.05
**32**	0	0	0	0	0	0	0	0	0	0.00	0.00
**34**	0	0	0	0	0	0	0	0	0	0.00	0.00
**36**	0.5	0	0	0	0	0	0	0	0	0.06	0.05
**38**	0	0	0	0	0	0	0	0	0	0.00	0.00
**42**	0	0	0	0	0	0	0	0	0	0.00	0.00
**46**	0	0	0	0	0	0	0	0	0	0.00	0.00
**50**	0	0	0	0	0	0	0	0	0	0.00	0.00
**54**	0	0	0	0	0	0	0	0	0	0.00	0.00
**57**	0	0	0	0	0	0	0	0	0	0.00	0.00
**61**	0	0	0	0	0	0	0	0	0	0.00	0.00
**68**	0	0	0	0	0	0	0	0	0	0.00	0.00
**75**	0	0	0	0	0	0	0	0	0	0.00	0.00
**82**	0	0	0	0	0	0	0	0	0	0.00	0.00
**89**	0	0	0	0	0	0	0	0	0	0.00	0.00
**96**	0	0	0	0	0	0	0	0	0	0.00	0.00
**103**	0	0	0	0	0	0	0	0	0	0.00	0.00
**110**	0	0	0	0	0	0	0	0	0	0.00	0.00
**117**	0	0	0	0	0	0	0	0	0	0.00	0.00
**124**	0	0	0	0	0	0	0	0	0	0.00	0.00
**131**	0	0	0	0	0	0	0	0	0	0.00	0.00
**138**	0	0	0	0	0	0	0	0	0	0.00	0.00
**145**	0	0	0	0	0	0	0	0	0	0.00	0.00
**151**	0	0	0	0	0	0	0	0	0	0.00	0.00
**159**	0	0	0	0	0	0	0	0	0	0.00	0.00
**168**	0	0	0	0	0	0	0	0	0	0.00	0.00
**175**	0	0	0	0	0	0	0	0	0	0.00	0.00
**Erythema score**	**Flash930 dose rate 23 Gy**										
**time after irradiation [d]**	Mouse ID: 5	7	13	17	35	37	55			mean	SEM
**0**	0	0	0	0	0	0	0			0.00	0.00
**2**	0.5	0	0	0	0	0	0			0.07	0.07
**4**	0	0	0.5	0	0	0	0			0.07	0.07
**6**	0	0	0	0	0	0	0			0.00	0.00
**8**	0	0	0	0	0	0	0			0.00	0.00
**10**	0	0	0	0	0	0	0			0.00	0.00
**12**	0	0	0	0	0	0	0			0.00	0.00
**14**	0	0	0	0	0	0	0			0.00	0.00
**16**	0	0	0	0	0.5	0	0			0.07	0.07
**18**	0.5	0	0	0	0	0	0			0.07	0.07
**20**	0	0	0	0	0.5	0	0			0.07	0.07
**22**	0	0	0.5	0	0.5	0	0			0.14	0.09
**24**	0	0	0	0	0	0	0			0.00	0.00
**26**	0	0	0	0	0	0	0			0.00	0.00

**28**	0	0	0	0	0	0	0			0.00	0.00
**30**	0	0	0	0	0	0	0			0.00	0.00
**32**	0	0	0	0	0	0	0			0.00	0.00
**34**	0	0	0	0	0	0	0			0.00	0.00
**36**	0	0	0	0	0	0	0			0.00	0.00
**38**	0	0	0	0	0	0.5	0			0.07	0.07
**42**	0	0	0	0	0	0	0			0.00	0.00
**46**	0	0	0	0	0	0	0			0.00	0.00
**50**	0	0	0	0	0	0	0			0.00	0.00
**54**	0	0	0	0	0	0	0			0.00	0.00
**57**	0	0	0	0	0	0	0			0.00	0.00
**61**	0	0	0	0	0	0	0			0.00	0.00
**68**	0	0	0	0	0	0	0			0.00	0.00
**75**	0	0	0	0	0	0	0			0.00	0.00
**82**	0	0	0	0	0	0	0			0.00	0.00
**89**	0	0	0	0	0	0	0			0.00	0.00
**96**	0	0	0	0	0	0	0			0.00	0.00
**103**	0	0	0	0	0	0	0			0.00	0.00
**110**	0	0	0	0	0	0	0			0.00	0.00
**117**	0	0	0	0	0	0	0			0.00	0.00
**124**	0	0	0	0	0	0	0			0.00	0.00
**131**	0	0	0	0	0	0	0			0.00	0.00
**138**	0	0	0	0	0	0	0			0.00	0.00
**145**	0	0	0	0	0	0	0			0.00	0.00
**151**	0	0	0	0	0	0	0			0.00	0.00
**159**	0	0	0	0	0	0	0			0.00	0.00
**168**	0	0	0	0	0	0	0			0.00	0.00
**175**	0	0	0	0	0	0	0			0.00	0.00
**Erythema score**	**Conventional dose rate 33 Gy**										
**time after irradiation [d]**	Mouse ID: 8	26	34	40	48	54	62			mean	SEM
**0**	0	0	0	0	0	0	0			0.00	0.00
**2**	0	0	0	0	0	0.5	0			0.07	0.07
**4**	0.5	0	0	0	0	0	0			0.07	0.07
**6**	0	0	0	0	0	0	0			0.00	0.00
**8**	0	0	0	0	0	0	0			0.00	0.00
**10**	0	0	0	0	0	0	0			0.00	0.00
**12**	0.5	0	0	0	0	0	0			0.07	0.07
**14**	0.5	0	0	0	0	0	0			0.07	0.07
**16**	1.5	0	1.5	0	0	0	1.5			0.64	0.28
**18**	1.5	0.5	1.5	0.5	0	0.5	0.5			0.71	0.20
**20**	1.5	0.5	3	1.5	1.5	0	0.5			1.2	0.3
**22**	3	0.5	1.5	1.5	0.5	0	1.5			1.2	0.3
**24**	3	0.5	0.5	1.5	0.5	0	0.5			0.9	0.4

**26**	1.5	0	0.5	1.5	0.5	0	0			0.57	0.24
**28**	1.5	0	0	0.5	0	0	0			0.29	0.20
**30**	0.5	0	0	0	0	0	0			0.07	0.07
**32**	0.5	0	0	0	0	0	0			0.07	0.07
**34**	0	0	0	0	0	0	0			0.00	0.00
**36**	0	0	0	0	0	0	0			0.00	0.00
**38**	0	0	0	0	0	0	0			0.00	0.00
**42**	0	0	0	0	0	0	0			0.00	0.00
**46**	0	0	0	0	0	0	0			0.00	0.00
**50**	0	0	0	0	0	0	0			0.00	0.00
**54**	0	0	0	0	0	0	0			0.00	0.00
**57**	0	0	0	0	0	0	0			0.00	0.00
**61**	0	0	0	0	0	0	0			0.00	0.00
**68**	0	0	0	0	0	0	0			0.00	0.00
**75**	0	0	0	0	0	0	0			0.00	0.00
**82**	0	0	0	0	0	0	0			0.00	0.00
**89**	0	0	0	0	0	0	0			0.00	0.00
**96**	0	0	0	0	0	0	0			0.00	0.00
**103**	0	0	0	0	0	0	0			0.00	0.00
**110**	0	0	0	0	0	0	0			0.00	0.00
**117**	0	0	0	0	0	0	0			0.00	0.00
**124**	0	0	0	0	0	0	0			0.00	0.00
**131**	0	0	0	0	0	0	0			0.00	0.00
**138**	0	0	0	0	0	0	0			0.00	0.00
**145**	0	0	0	0	0	0	0			0.00	0.00
**151**	0	0	0	0	0	0	0			0.00	0.00
**159**	0	0	0	0	0	0	0			0.00	0.00
**168**	0	0	0.5	0	0	0	0			0.07	0.07
**175**	0.5	0	0	0	0	0	0			0.07	0.07

**Erythema score**	**Flash9 dose rate 33 Gy**										
**time after irradiation [d]**	Mouse ID: 1	10	58							mean	SEM
**0**	0	0	0							0.00	0.00
**2**	0	0	0							0.00	0.00
**4**	0	0	0							0.00	0.00
**6**	0	0	0							0.00	0.00
**8**	0	0	0							0.00	0.00
**10**	0	0	0							0.00	0.00
**12**	0	0	0							0.00	0.00
**14**	0	0	0							0.00	0.00
**16**	0	0	0							0.00	0.00
**18**	1.5	0	0							0.5	0.4
**20**	0.5	0	0							0.17	0.14
**22**	0.5	0	0							0.17	0.14

**24**	0	0	0							0.00	0.00
**26**	0	0	0							0.00	0.00
**28**	0	0	0							0.00	0.00
**30**	0	0	0							0.00	0.00
**32**	0	0	0							0.00	0.00
**34**	0	0	0							0.00	0.00
**36**	0	0	0							0.00	0.00
**38**	0	0	0							0.00	0.00
**42**	0	0	0							0.00	0.00
**46**	0	0	0							0.00	0.00
**50**	0	0	0							0.00	0.00
**54**	0	0	0							0.00	0.00
**57**	0	0	0							0.00	0.00
**61**	0	0	0							0.00	0.00
**68**	0	0	0							0.00	0.00
**75**	0	0	0							0.00	0.00
**82**	0	0	0							0.00	0.00
**89**	0	0	0							0.00	0.00
**96**	0	0	0							0.00	0.00
**103**	0	0	0							0.00	0.00
**110**	0	0	0							0.00	0.00
**117**	0	0	0							0.00	0.00
**124**	0	0	0							0.00	0.00
**131**	0	0	0							0.00	0.00
**138**	0	0	0							0.00	0.00
**145**	0	0	0							0.00	0.00
**151**	0	0	0							0.00	0.00
**159**	0	0	0							0.00	0.00
**168**	0	0	0							0.00	0.00
**175**	0	0	0							0.00	0.00
**Erythema score**	**Flash930 dose rate 33 Gy**										
**time after irradiation [d]**	Mouse ID: 9	19	21	23	41	47	49	61	63	mean	SEM
**0**	0	0	0	0	0	0	0	0	0	0.00	0.00
**2**	0.5	0	0.5	0	0	0	0	0	0	0.11	0.07
**4**	0.5	0	0	0	0	0	0	0	0	0.06	0.05
**6**	0	0	0	0	0	0	0	0	0	0.00	0.00
**8**	0	0	0	0	0	0	0	0	0	0.00	0.00
**10**	0	0	0	0	0	0	0	0	0	0.00	0.00
**12**	0	0	0	0	0	0	0	0	0	0.00	0.00
**14**	0	0	0	0	0	0	0	0	0	0.00	0.00
**16**	0.5	0	0	0	0	0.5	0	0.5	0.5	0.22	0.08
**18**	0.5	0.5	0.5	0.5	0	0.5	0.5	0	0.5	0.39	0.07
**20**	0.5	0.5	0.5	0.5	0	0	0.5	0.5	0.5	0.39	0.07

**22**	0	1.5	0	0.5	0.5	0	0	0.5	1.5	0.50	0.19
**24**	0	1.5	0	0.5	1.5	0	0	0.5	0	0.44	0.20
**26**	0	1.5	0	0	0	0	0	0.5	0	0.22	0.16
**28**	0	0.5	0	0	0	0	0	0	0	0.06	0.05
**30**	0	0	0	0	0	0	0	0	0	0.00	0.00
**32**	0	0	0	0	0	0	0	0	0	0.00	0.00
**34**	0	0	0	0	0	0	0	0	0	0.00	0.00
**36**	0	0	0	0	0	0	0	0	0	0.00	0.00
**38**	0	0	0	0	0	0	0	0	0	0.00	0.00
**42**	0	0	0	0	0	0	0	0	0	0.00	0.00
**46**	0	0	0	0	0	0	0	0	0	0.00	0.00
**50**	0	0	0	0	0	0	0	0	0	0.00	0.00
**54**	0	0	0	0	0	0	0	0	0	0.00	0.00
**57**	0	0	0	0	0	0	0	0	0	0.00	0.00
**61**	0	0	0	0	0	0	0	0	0	0.00	0.00
**68**	0	0	0	0	0	0	0	0	0	0.00	0.00
**75**	0	0	0	0	0	0	0	0	0	0.00	0.00
**82**	0	0	0	0	0	0	0	0	0	0.00	0.00
**89**	0	0	0	0	0	0	0	0	0	0.00	0.00
**96**	0	0	0	0	0	0	0	0	0	0.00	0.00
**103**	0	0	0	0	0	0	0	0	0	0.00	0.00
**110**	0	0	0	0	0	0	0	0	0	0.00	0.00
**117**	0	0	0	0	0	0	0	0	0	0.00	0.00
**124**	0	0	0	0	0	0	0	0	0	0.00	0.00
**131**	0	0	0	0	0	0	0	0	0	0.00	0.00
**138**	0	0	0	0	0	0	0	0	0	0.00	0.00
**145**	0	0	0	0	0	0	0	0	0	0.00	0.00
**151**	0	0	0	0	0	0	0	0	0	0.00	0.00
**159**	0	0	0	0	0	0	0	0	0	0.00	0.00
**168**	0	0	0	0	0	0	0	0	0	0.00	0.00
**175**	0	0	0	0	0	0	0	0	0	0.00	0.00
**Erythema score**	**Sham irradiated 0 Gy**										
**time after irradiation [d]**	Mouse ID: 15	24	25	28	29	45	59			mean	SEM
**0**	0	0	0	0	0	0	0			0.00	0.00
**2**	0	0.5	0	0	0	0	0			0.07	0.07
**4**	0	0	0.5	0	0	0	0			0.07	0.07
**6**	0	0	0	0	0	0	0			0.00	0.00
**8**	0	0	0	0	0	0	0			0.00	0.00
**10**	0	0	0	0	0	0	0			0.00	0.00
**12**	0	0	0	0	0	0	0			0.00	0.00
**14**	0	0	0	0	0	0	0			0.00	0.00
**16**	0	0	0	0	0	0	0			0.00	0.00
**18**	0	0	0	0	0	0	0			0.00	0.00

**20**	0	0	0	0	0	0	0			0.00	0.00
**22**	0	0	0	0	0	0	0			0.00	0.00
**24**	0	0	0	0	0	0	0			0.00	0.00
**26**	0	0	0	0	0	0	0			0.00	0.00
**28**	0	0	0	0	0	0	0			0.00	0.00
**30**	0	0	0	0	0	0	0			0.00	0.00
**32**	0	0	0	0	0	0	0			0.00	0.00
**34**	0	0	0	0	0	0	0			0.00	0.00
**36**	0	0	0	0	0	0	0			0.00	0.00
**38**	0	0	0	0.5	0	0	0			0.07	0.07
**42**	0	0	0	0	0	0	0			0.00	0.00
**46**	0	0	0	0	0	0	0			0.00	0.00
**50**	0	0	0	0	0	0	0			0.00	0.00
**54**	0	0	0	0	0.5	0	0			0.07	0.07
**57**	0	0	0	0	0.5	0	0			0.07	0.07
**61**	0	0	0	0	0.5	0	0			0.07	0.07
**68**	0	0	0	0	0	0	0			0.00	0.00
**75**	0	0	0	0	0	0	0			0.00	0.00
**82**	0	0	0	0	0	0	0			0.00	0.00
**89**	0	0	0	0	0	0	0			0.00	0.00
**96**	0	0	0	0	0	0	0			0.00	0.00
**103**	0	0	0	0	0	0	0			0.00	0.00
**110**	0	0	0	0	0	0	0			0.00	0.00
**117**	0	0	0	0	0	0	0			0.00	0.00
**124**	0	0	0	0	0	0	0			0.00	0.00
**131**	0	0	0	0.5	0	0	0			0.07	0.07
**138**	0	0	0	0	0	0	0			0.00	0.00
**145**	0	0	0	0	0	0	0			0.00	0.00
**151**	0	0	0	0	0	0	0			0.00	0.00
**159**	0	0	0	0	0	0	0			0.00	0.00
**168**	0	0	0	0	0	0	0			0.00	0.00
**175**	0	0	0	0	0	0	0			0.00	0.00
**Desquamation score**	**Conventional dose rate 23 Gy**										
**time after irradiation [d]**	Mouse ID: 18	22	32	36	46	50	60			mean	SEM
**0**	0	0	0	0	0	0	0			0.00	0.00
**2**	0	0	0	0	0	0	0			0.00	0.00
**4**	0	0	0	0	0	0	0			0.00	0.00
**6**	0	0	0	0	0	0	0			0.00	0.00
**8**	0	0	0	0	0	0	0			0.00	0.00
**10**	0	0	0	0	0	0	0			0.00	0.00
**12**	0	0	0	0	0	0	0			0.00	0.00
**14**	0	0	0	0	0	0	0			0.00	0.00
**16**	0	0	0	0	0	0	0			0.00	0.00

**18**	0	0	0	0	0	0	0			0.00	0.00
**20**	0	0	0	0	0	0	0			0.00	0.00
**22**	0	0	1	0	0	0	0			0.14	0.13
**24**	1	1	0	0	0	1	1			0.57	0.19
**26**	1	1	0	1	0	0	0			0.43	0.19
**28**	0	1	0	1	0	0	0			0.29	0.17
**30**	0	0	1	1	1	0	1			0.57	0.19
**32**	0	1	1	0	0	0	0			0.29	0.17
**34**	0	1	1	0	0	1	0			0.43	0.19
**36**	1	0	1	0	0	0	0			0.29	0.17
**38**	0	1	1	0	0	0	0			0.29	0.17
**42**	0	0	0	1	0	0	0			0.14	0.13
**46**	0	0	0	0	0	0	0			0.00	0.00
**50**	0	1	0	0	0	0	0			0.14	0.13
**54**	0	0	0	0	0	0	0			0.00	0.00
**57**	0	0	0	0	0	0	0			0.00	0.00
**61**	0	0	0	0	0	0	0			0.00	0.00
**68**	0	0	0	0	0	0	0			0.00	0.00
**75**	0	0	0	0	0	0	0			0.00	0.00
**82**	0	0	0	0	0	0	0			0.00	0.00
**89**	0	0	0	0	0	0	0			0.00	0.00
**96**	0	0	0	0	0	0	0			0.00	0.00
**103**	0	0	0	0	0	0	0			0.00	0.00
**110**	0	0	0	0	0	0	0			0.00	0.00
**117**	0	0	0	0	0	0	0			0.00	0.00
**124**	0	0	0	0	0	0	0			0.00	0.00
**131**	0	0	0	0	0	0	0			0.00	0.00
**138**	0	0	0	0	0	0	0			0.00	0.00
**145**	0	0	0	0	0	0	0			0.00	0.00
**151**	0	0	0	0	0	0	0			0.00	0.00
**159**	0	0	0	0	0	0	0			0.00	0.00
**168**	0	0	0	0	0	0	0			0.00	0.00
**175**	0	0	0	0	0	0	0			0.00	0.00
**Desquamation score**	**Flash9 dose rate 23 Gy**										
**time after irradiation [d]**	Mouse ID: 31	38	39	43	44	52	53	56	57	mean	SEM
**0**	0	0	0	0	0	0	0	0	0	0.00	0.00
**2**	0	0	0	0	0	0	0	0	0	0.00	0.00
**4**	0	0	0	0	0	0	0	0	0	0.00	0.00
**6**	0	0	0	0	0	0	0	0	0	0.00	0.00
**8**	0	0	0	0	0	0	0	0	0	0.00	0.00
**10**	0	0	0	0	0	0	0	0	0	0.00	0.00
**12**	0	0	0	0	0	0	0	0	0	0.00	0.00
**14**	0	0	0	0	0	0	0	0	0	0.00	0.00

**16**	0	0	0	0	0	0	0	0	0	0.00	0.00
**18**	0	0	0	0	0	0	0	0	0	0.00	0.00
**20**	0	0	0	0	0	0	0	0	0	0.00	0.00
**22**	0	0	1	1	0	0	0	0	0	0.22	0.14
**24**	0	0	0	0	0	1	1	1	1	0.44	0.17
**26**	1	1	0	0	0	0	1	0	0	0.33	0.16
**28**	0	0	0	0	1	0	0	0	0	0.11	0.10
**30**	1	0	0	0	1	0	0	0	0	0.22	0.14
**32**	1	0	0	0	1	0	0	0	0	0.22	0.14
**34**	0	0	0	0	1	0	0	0	0	0.11	0.10
**36**	0	0	0	0	0	0	0	0	0	0.00	0.00
**38**	0	0	0	1	1	0	0	0	1	0.33	0.16
**42**	0	0	0	0	1	0	0	0	0	0.11	0.10
**46**	0	0	0	0	0	0	1	0	0	0.11	0.10
**50**	0	0	0	0	0	0	0	0	0	0.00	0.00
**54**	0	0	0	0	0	0	0	1	0	0.11	0.10
**57**	0	0	0	0	0	0	0	0	0	0.00	0.00
**61**	0	0	0	0	0	0	0	0	0	0.00	0.00
**68**	0	0	0	0	0	0	0	0	0	0.00	0.00
**75**	0	0	0	0	0	0	0	0	0	0.00	0.00
**82**	0	0	0	0	0	0	0	0	0	0.00	0.00
**89**	0	0	0	0	0	0	0	0	0	0.00	0.00
**96**	0	0	0	0	0	0	0	0	0	0.00	0.00
**103**	0	0	0	0	0	0	0	0	0	0.00	0.00
**110**	0	0	0	0	0	0	0	0	0	0.00	0.00
**117**	0	0	0	0	0	0	0	0	0	0.00	0.00
**124**	0	0	0	0	0	0	0	0	0	0.00	0.00
**131**	0	0	0	0	0	0	0	0	0	0.00	0.00
**138**	0	0	0	0	0	0	0	0	0	0.00	0.00
**145**	0	0	0	0	0	0	0	0	0	0.00	0.00
**151**	0	0	0	0	0	0	0	0	0	0.00	0.00
**159**	0	0	0	0	0	0	0	0	0	0.00	0.00
**168**	0	0	0	0	0	0	0	0	0	0.00	0.00
**175**	0	0	0	0	0	0	0	0	0	0.00	0.00
**Desquamation score**	**Flash930 dose rate 23 Gy**										
**time after irradiation [d]**	Mouse ID: 5	7	13	17	35	37	55			mean	SEM
**0**	0	0	0	0	0	0	0			0.00	0.00
**2**	0	0	0	0	0	0	0			0.00	0.00
**4**	0	0	0	0	0	0	0			0.00	0.00
**6**	0	0	0	0	0	0	0			0.00	0.00
**8**	0	0	0	0	0	0	0			0.00	0.00
**10**	0	0	0	0	0	0	0			0.00	0.00
**12**	0	0	0	0	0	0	0			0.00	0.00

**14**	0	0	0	0	0	0	0			0.00	0.00
**16**	0	0	0	0	0	0	0			0.00	0.00
**18**	0	0	0	0	0	0	0			0.00	0.00
**20**	0	0	0	0	1	0	0			0.14	0.13
**22**	0	0	1	0	1	0	0			0.29	0.17
**24**	0	0	0	1	0	0	1			0.29	0.17
**26**	0	1	0	0	0	0	0			0.14	0.13
**28**	0	0	0	1	0	1	0			0.29	0.17
**30**	0	0	0	0	0	1	0			0.14	0.13
**32**	0	0	0	0	1	0	0			0.14	0.13
**34**	0	0	0	0	0	0	0			0.00	0.00
**36**	0	0	0	0	0	0	0			0.00	0.00
**38**	0	0	0	0	0	1	0			0.14	0.13
**42**	0	0	0	0	1	0	0			0.14	0.13
**46**	0	0	0	0	0	0	0			0.00	0.00
**50**	0	0	0	0	0	0	0			0.00	0.00
**54**	0	0	0	0	0	0	0			0.00	0.00
**57**	0	0	0	0	0	0	0			0.00	0.00
**61**	0	0	0	0	0	0	0			0.00	0.00
**68**	0	0	0	0	0	0	0			0.00	0.00
**75**	0	0	0	0	0	0	0			0.00	0.00
**82**	0	0	0	0	0	0	0			0.00	0.00
**89**	0	0	0	0	0	0	0			0.00	0.00
**96**	0	0	0	0	0	0	0			0.00	0.00
**103**	0	0	0	0	0	0	0			0.00	0.00
**110**	0	0	0	0	0	0	0			0.00	0.00
**117**	0	0	0	0	0	0	0			0.00	0.00
**124**	0	0	0	0	0	0	0			0.00	0.00
**131**	0	0	0	0	0	0	0			0.00	0.00
**138**	0	0	0	0	0	0	0			0.00	0.00
**145**	0	0	0	0	0	0	0			0.00	0.00
**151**	0	0	0	0	0	0	0			0.00	0.00
**159**	0	0	0	0	0	0	0			0.00	0.00
**168**	0	0	0	0	0	0	0			0.00	0.00
**175**	0	0	0	0	0	0	0			0.00	0.00
**Desquamation score**	**Conventional dose rate 33 Gy**										
**time after irradiation [d]**	Mouse ID: 8	26	34	40	48	54	62			mean	SEM
**0**	0	0	0	0	0	0	0			0.00	0.00
**2**	0	0	0	0	0	0	0			0.00	0.00
**4**	0	0	0	0	0	0	0			0.00	0.00
**6**	0	0	0	0	0	0	0			0.00	0.00
**8**	0	0	0	0	0	0	0			0.00	0.00
**10**	0	0	0	0	0	0	0			0.00	0.00

**12**	0	0	0	0	0	0	0			0.00	0.00
**14**	0	0	0	0	0	0	0			0.00	0.00
**16**	0	0	1	1	0	0	0			0.29	0.17
**18**	0	0	1	1	0	0	0			0.29	0.17
**20**	1	0	2	1	1	0	1			0.86	0.24
**22**	2	1	2	1	1	0	1			1.14	0.24
**24**	2	1	1	1	1	0	1			1.00	0.20
**26**	2	0	1	1	1	0	0			0.71	0.26
**28**	1	1	1	1	1	0	1			0.86	0.13
**30**	1	1	1	0	1	1	1			0.86	0.13
**32**	1	1	1	0	1	0	1			0.71	0.17
**34**	1	0	0	1	0	0	1			0.43	0.19
**36**	1	0	0	0	0	0	1			0.29	0.17
**38**	1	0	0	0	0	1	0			0.29	0.17
**42**	1	0	1	0	0	0	0			0.29	0.17
**46**	1	0	1	0	0	0	0			0.29	0.17
**50**	0	0	0	0	0	0	0			0.00	0.00
**54**	1	0	0	0	0	0	0			0.14	0.13
**57**	0	0	0	0	0	0	0			0.00	0.00
**61**	1	0	0	0	0	0	0			0.14	0.13
**68**	0	0	0	0	0	0	0			0.00	0.00
**75**	0	0	0	0	0	0	0			0.00	0.00
**82**	0	0	0	0	0	0	0			0.00	0.00
**89**	0	0	0	0	0	0	0			0.00	0.00
**96**	0	0	0	0	0	0	0			0.00	0.00
**103**	0	0	0	0	0	0	0			0.00	0.00
**110**	0	0	0	0	0	0	0			0.00	0.00
**117**	0	0	0	0	0	0	0			0.00	0.00
**124**	0	0	0	0	0	0	0			0.00	0.00
**131**	0	0	0	0	0	0	0			0.00	0.00
**138**	0	0	0	0	0	0	0			0.00	0.00
**145**	0	0	0	0	0	0	0			0.00	0.00
**151**	0	0	0	0	0	0	0			0.00	0.00
**159**	0	0	0	0	0	0	0			0.00	0.00
**168**	0	0	0	0	0	0	0			0.00	0.00
**175**	0	0	0	0	0	0	0			0.00	0.00
**Desquamation score**	**Flash9 dose rate 33 Gy**										
**time after irradiation [d]**	Mouse ID: 1	10	58							mean	SEM
**0**	0	0	0							0.00	0.00
**2**	0	0	0							0.00	0.00
**4**	0	0	0							0.00	0.00
**6**	0	0	0							0.00	0.00
**8**	0	0	0							0.00	0.00

**10**	0	0	0							0.00	0.00
**12**	0	0	0							0.00	0.00
**14**	0	0	0							0.00	0.00
**16**	0	0	0							0.00	0.00
**18**	1	0	0							0.33	0.27
**20**	1	0	0							0.33	0.27
**22**	1	0	1							0.67	0.27
**24**	1	0	1							0.67	0.27
**26**	0	0	0							0.00	0.00
**28**	0	0	1							0.33	0.27
**30**	0	0	0							0.00	0.00
**32**	0	0	0							0.00	0.00
**34**	0	0	1							0.33	0.27
**36**	0	0	0							0.00	0.00
**38**	0	0	0							0.00	0.00
**42**	0	0	0							0.00	0.00
**46**	0	0	0							0.00	0.00
**50**	1	0	0							0.33	0.27
**54**	1	0	0							0.33	0.27
**57**	0	0	0							0.00	0.00
**61**	0	0	0							0.00	0.00
**68**	0	0	0							0.00	0.00
**75**	0	0	0							0.00	0.00
**82**	0	0	0							0.00	0.00
**89**	0	0	0							0.00	0.00
**96**	0	0	0							0.00	0.00
**103**	0	0	0							0.00	0.00
**110**	0	0	0							0.00	0.00
**117**	0	0	0							0.00	0.00
**124**	0	0	0							0.00	0.00
**131**	0	0	0							0.00	0.00
**138**	0	0	0							0.00	0.00
**145**	0	0	0							0.00	0.00
**151**	0	0	0							0.00	0.00
**159**	0	0	0							0.00	0.00
**168**	0	0	0							0.00	0.00
**175**	0	0	0							0.00	0.00
**Desquamation score**	**Flash930 dose rate 33 Gy**										
**time after irradiation [d]**	Mouse ID: 9	19	21	23	41	47	49	61	63	mean	SEM
**0**	0	0	0	0	0	0	0	0	0	0.00	0.00
**2**	0	0	0	0	0	0	0	0	0	0.00	0.00
**4**	0	0	0	0	0	0	0	0	0	0.00	0.00
**6**	0	0	0	0	0	0	0	0	0	0.00	0.00

**8**	0	0	0	0	0	0	0	0	0	0.00	0.00
**10**	0	0	0	0	0	0	0	0	0	0.00	0.00
**12**	0	0	0	0	0	0	0	0	0	0.00	0.00
**14**	0	0	0	0	0	0	0	0	0	0.00	0.00
**16**	0	0	0	0	0	0	0	0	0	0.00	0.00
**18**	0	0	0	0	0	0	0	0	0	0.00	0.00
**20**	1	1	0	0	0	0	1	1	1	0.56	0.17
**22**	1	1	0	1	1	0	0	1	1	0.67	0.16
**24**	0	1	0	1	2	0	1	1	1	0.78	0.21
**26**	0	1	1	1	1	0	0	0	1	0.56	0.17
**28**	0	1	0	0	0	0	0	0	0	0.11	0.10
**30**	1	1	1	0	0	0	0	0	1	0.44	0.17
**32**	0	1	1	0	0	0	0	0	0	0.22	0.14
**34**	0	0	0	0	0	0	0	0	1	0.11	0.10
**36**	0	0	0	0	0	0	0	0	0	0.00	0.00
**38**	0	0	1	0	0	0	0	1	0	0.22	0.14
**42**	0	0	0	0	0	0	0	0	0	0.00	0.00
**46**	0	0	0	0	1	0	0	0	0	0.11	0.10
**50**	0	0	0	0	0	0	0	0	0	0.00	0.00
**54**	0	0	0	0	1	0	0	0	0	0.11	0.10
**57**	0	0	0	0	0	0	0	0	0	0.00	0.00
**61**	0	1	0	0	0	0	0	0	0	0.11	0.10
**68**	1	0	0	0	0	0	0	0	0	0.11	0.10
**75**	0	0	0	0	0	0	0	0	0	0.00	0.00
**82**	0	0	0	0	0	0	0	0	0	0.00	0.00
**89**	0	0	0	0	0	0	0	0	0	0.00	0.00
**96**	0	0	0	0	0	0	0	0	0	0.00	0.00
**103**	0	0	0	0	0	0	0	0	0	0.00	0.00
**110**	0	0	0	0	0	0	0	0	0	0.00	0.00
**117**	0	0	0	0	0	0	0	0	0	0.00	0.00
**124**	0	0	0	0	0	0	0	0	0	0.00	0.00
**131**	0	0	0	0	0	0	0	0	0	0.00	0.00
**138**	0	0	0	0	0	0	0	0	0	0.00	0.00
**145**	0	0	0	0	0	0	0	0	0	0.00	0.00
**151**	0	0	0	0	0	0	0	0	0	0.00	0.00
**159**	0	0	0	0	0	0	0	0	0	0.00	0.00
**168**	0	0	0	0	0	0	0	0	0	0.00	0.00
**175**	0	0	0	0	0	0	0	0	0	0.00	0.00
**Desquamation score**	**Sham irradiated 0 Gy**										
**time after irradiation [d]**	Mouse ID: 15	24	25	28	29	45	59			mean	SEM
**0**	0	0	0	0	0	0	0			0.00	0.00
**2**	0	0	0	0	0	0	0			0.00	0.00
**4**	0	0	0	0	0	0	0			0.00	0.00

**6**	0	0	0	0	0	0	0			0.00	0.00
**8**	0	0	0	0	0	0	0			0.00	0.00
**10**	0	0	0	0	0	0	0			0.00	0.00
**12**	0	0	0	0	0	0	0			0.00	0.00
**14**	0	0	0	0	0	0	0			0.00	0.00
**16**	0	0	0	0	0	0	0			0.00	0.00
**18**	0	0	0	0	0	0	0			0.00	0.00
**20**	0	0	0	0	0	0	0			0.00	0.00
**22**	0	0	0	0	0	0	0			0.00	0.00
**24**	0	0	0	0	0	0	0			0.00	0.00
**26**	0	0	0	0	0	0	0			0.00	0.00
**28**	0	0	0	0	0	0	0			0.00	0.00
**30**	0	0	0	0	0	1	0			0.14	0.13
**32**	0	0	0	0	0	0	0			0.00	0.00
**34**	0	0	0	0	0	0	0			0.00	0.00
**36**	0	0	0	0	0	0	0			0.00	0.00
**38**	0	0	0	0	0	0	0			0.00	0.00
**42**	0	0	0	0	0	0	0			0.00	0.00
**46**	0	0	0	0	0	0	0			0.00	0.00
**50**	0	0	0	0	0	0	0			0.00	0.00
**54**	0	0	0	0	0	0	0			0.00	0.00
**57**	0	0	0	0	2	0	0			0.29	0.26
**61**	0	0	0	0	0	0	0			0.00	0.00
**68**	0	0	0	0	0	0	0			0.00	0.00
**75**	0	0	0	0	0	0	0			0.00	0.00
**82**	0	0	0	0	0	0	0			0.00	0.00
**89**	0	0	0	0	0	0	0			0.00	0.00
**96**	0	0	0	0	0	0	0			0.00	0.00
**103**	0	0	0	0	0	0	0			0.00	0.00
**110**	0	0	0	0	0	0	0			0.00	0.00
**117**	0	0	0	0	0	0	0			0.00	0.00
**124**	0	0	0	0	0	0	0			0.00	0.00
**131**	0	0	0	0	0	0	0			0.00	0.00
**138**	0	0	0	0	0	0	0			0.00	0.00
**145**	0	0	0	0	0	0	0			0.00	0.00
**151**	0	0	0	0	0	0	0			0.00	0.00
**159**	0	0	0	0	0	0	0			0.00	0.00
**168**	0	0	0	0	0	0	0			0.00	0.00
**175**	0	0	0	0	0	0	0			0.00	0.00
**Ear swelling**	**Conventional dose rate 23 Gy**										
**time after irradiation [d]**	Mouse ID: 18	22	32	36	46	50	60			mean	SEM
**0**	20	-1	9	-21	-13	-3	17			1	5
**2**	7	0	10	-3	-7	-10	7			0	3
**4**	-13	-13	-5	-8	-15	-43	-3			-15	5

**6**	2	-11	14	2	5	2	7			3	3
**8**	-12	-20	3	8	-9	-20	-14			-9	4
**10**	18	-2	16	13	26	1	11			12	3
**12**	33	3	6	-4	-7	-27	-4			0	6
**14**	27	-16	1	11	7	-16	-4			1	5
**16**	11	4	19	27	16	-3	14			13	3
**18**	39	25	15	22	24	7	14			21	4
**20**	36	111	36	23	43	9	13			39	12
**22**	60	100	43	71	38	20	11			49	11
**24**	26	66	30	68	30	25	30			39	7
**26**	19	52	59	56	19	32	1			34	8
**28**	11	36	44	58	13	18	11			27	7
**30**	13	28	36	58	16	11	9			24	6
**32**	-4	11	23	34	11	6	11			13	4
**34**	-7	3	-5	20	-7	-7	-7			-2	4
**36**	8	26	10	28	11	25	15			18	3
**38**	8	15	20	13	1	-7	-2			7	3
**42**	0	4	-1	20	-3	-8	2			2	3
**46**	2.1	2.1	3.8	17.1	0.5	2.1	10.5			5.5	2.1
**50**	-18	8	8	5	7	3	-3			1	3
**54**	-14.5	5.5	-1.2	0.5	0.5	-2.9	-2.9			-2.1	2.2
**57**	-5	3	-7	8	-14	-9	-4			-4	3
**61**	6.4	6.4	4.8	1.4	-6.9	-3.6	-0.2			1.2	1.8
**68**	-1.7	0.0	-3.3	-1.7	-10.0	-6.7	-3.3			-3.8	1.2
**75**	-17.1	-0.5	-10.5	1.2	-12.1	-13.8	-5.5			-8.3	2.4
**82**	-9.8	-9.8	-8.1	-1.4	-14.8	-16.4	-9.8			-10.0	1.7
**89**	-2.1	-12.1	-13.8	-17.1	-7.1	-5.5	-12.1			-10.0	1.8
**96**	-9.5	-14.5	-6.2	-12.9	0.5	-7.9	-12.9			-9.0	1.8
**103**	11	2	-8	-6	-11	-16	-6			-5	3
**110**	-6	-11	-12	-2	-14	9	-12			-7	3
**117**	-3.8	-8.8	-5.5	-0.5	-3.8	-17.1	-7.1			-6.7	1.9
**124**	-8.3	-3.3	5.0	0.0	-6.7	0.0	-10.0			-3.3	1.9
**131**	-0.7	-4.0	-10.7	-7.4	-4.0	1.0	-4.0			-4.3	1.4
**138**	0	-7	-7	6	13	-7	20			2	4
**145**	5	-7	-17	-9	8	15	-7			-2	4
**151**	-2.6	-1.0	-2.6	-2.6	-11.0	2.4	-17.6			-5.0	2.4
**159**	13	-5	-15	-8	-7	8	-5			-3	3
**168**	19	-1	-10	0	-10	2	-15			-2	4
**175**	-10	-33	-10	-3	-5	19	-23			-9	6

**Ear swelling**	**Flash9 dose rate 23 Gy**										
**time after irradiation [d]**	Mouse ID: 31	38	39	43	44	52	53	56	57	mean	SEM
**0**	-21	-15	-15	10	4	-3	9	-8	5	-4	4
**2**	-8	25	10	3	12	8	-13	5	22	7	4
**4**	7	7	-15	-20	-3	-7	-7	18	-8	-3	4
**6**	-2.9	8.8	2.1	7.1	7.1	5.5	-1.2	2.1	10.5	4.4	1.4
**8**	-8.6	-3.6	-15.2	-6.9	-15.2	-5.2	-1.9	-15.2	6.4	-7.3	2.3
**10**	19	14	-4	6	-6	16	6	9	14	8	3
**12**	12.6	-0.7	-4.0	2.6	-5.7	-4.0	7.6	4.3	-0.7	1.3	1.9
**14**	26	7	4	4	6	-8	-6	-6	9	4	3
**16**	19	-3	7	4	-4	-4	11	-1	24	6	3
**18**	34	15	29	9	32	-1	19	14	4	17	4
**20**	14	3	23	4	26	-16	51	11	1	13	6
**22**	48	1	38	18	33	16	8	33	13	23	5
**24**	135	5	38	6	46	28	48	60	-7	40	13
**26**	46	34	24	7	19	-4	19	69	9	25	7
**28**	78	14	3	4	28	14	13	41	1	22	8
**30**	58	-2	16	3	16	-12	31	4	13	14	6
**32**	36	-7	-7	-4	6	-19	11	6	1	2	5
**34**	25	-12	-2	-15	8	-7	21	1	6	3	4
**36**	48	0	1	8	-2	1	8	-4	18	9	5
**38**	8	-5	-7	-19	-4	-14	3	-9	3	-5	3
**42**	-1	-3	-20	-8	0	4	19	4	27	2	4
**46**	5	2	-16	-10	-11	-11	27	-11	7	-2	4
**50**	-3	-18	-17	2	-8	-12	12	-13	7	-6	3
**54**	-10	-10	-20	0	-1	-13	24	-3	7	-3	4
**57**	-7	-7	-19	-12	-19	-12	13	-14	-4	-9	3
**61**	3.1	-10.2	-11.9	-6.9	-1.9	-5.2	-1.9	-11.9	-1.9	-5.4	1.6
**68**	-1.7	3.3	-8.3	1.7	-1.7	-11.7	8.3	-11.7	8.3	-1.5	2.4
**75**	-10	-7	-15	-12	-22	5	-2	-5	8	-7	3
**82**	0	-15	-16	7	-13	-11	12	-11	4	-5	3
**89**	-15	-20	-24	-10	-5	-14	-5	-15	-5	-13	2
**96**	-3	-3	-15	-6	-11	-18	-3	-20	-1	-9	2
**103**	17	-11	-18	-6	-19	-9	1	-8	4	-5	4
**110**	-5.7	-10.7	-10.7	-9.0	-20.7	-2.4	2.6	-12.4	-4.0	-8.1	2.1
**117**	3	6	-4	6	-9	5	18	5	25	6	3
**124**	-8	5	-25	-8	-5	-3	-5	-17	23	-5	4
**131**	-26	-4	-19	-9	-7	-22	6	-17	-1	-11	3
**138**	1	21	-17	0	-5	-2	13	-14	20	2	4
**145**	1	6	-12	-7	-4	3	28	0	10	3	4
**151**	11	-8	-3	6	-14	-6	-9	4	4	-2	3

**159**	-2	-8	-13	-10	-15	-8	27	-10	10	-3	4
**168**	0.5	-2.9	-14.5	-9.5	-11.2	-14.5	-2.9	2.1	-1.2	-6.0	2.0
**175**	-6.4	6.9	-8.1	0.2	-3.1	-8.1	11.9	-8.1	6.9	-0.9	2.4
**Ear swelling**	**Flash930 dose rate 23 Gy**										
**time after irradiation [d]**	Mouse ID: 5	7	13	17	35	37	55			mean	SEM
**0**	0	7	29	24	4	-1	0			9	4
**2**	27	17	-2	-35	-2	12	7			3	7
**4**	7	-7	-8	8	2	8	-37			-4	6
**6**	25	-11	-11	15	-16	-1	-1			0	5
**8**	35	-4	16	-34	-27	21	-15			-1	9
**10**	11	-9	-4	-19	-7	14	1			-2	4
**12**	28	11	21	-37	-12	9	-14			1	8
**14**	4	9	24	-34	7	7	-14			0	7
**16**	31	27	16	-41	-19	-3	-18			-1	9
**18**	35	14	39	-10	-13	9	-5			10	7
**20**	33	31	71	-11	9	13	-2			20	10
**22**	33	16	115	-32	61	23	-4			30	17
**24**	51	23	105	-19	10	3	31			29	14
**26**	64	21	81	-18	39	2	2			27	12
**28**	31	6	64	-37	14	8	-12			10	11
**30**	41	8	56	-32	14	3	-17			10	11
**32**	16	-4	48	-31	-17	-4	-14			-1	9
**34**	15	-10	41	-35	3	-25	-9			-3	9
**36**	11	-7	36	-40	-4	-5	-2			-1	8
**38**	3	-9	31	-39	-19	-4	-12			-7	7
**42**	7	0	32	-40	2	2	-10			-1	7
**46**	2	-13	24	-38	-21	-1	-16			-9	7
**50**	-7	-12	17	-30	-17	-3	-10			-9	5
**54**	-11	-25	5	-48	-3	-6	-10			-14	6
**57**	-10	-15	0	-35	-5	-9	-27			-15	4
**61**	-4	-19	8	-35	1	-7	-30			-12	6
**68**	-5	-7	-3	-42	-2	-13	-2			-10	5
**75**	-2	-25	10	-44	3	-9	-24			-13	7
**82**	0	-18	19	-51	-8	-18	-20			-14	8
**89**	-5	-27	6	-32	-12	-30	-14			-16	5
**96**	-13	-10	-5	-38	-16	-18	-11			-16	4
**103**	-26	-18	-13	-53	-19	-14	-16			-23	5
**110**	-7	-14	-9	-41	-14	-16	-17			-17	4
**117**	-15	-4	5	-30	3	3	3			-5	5
**124**	-2	-13	-3	-23	0	-10	-37			-13	5
**131**	-12	-26	-16	-31	-19	-7	-1			-16	4
**138**	-7	-10	-4	-12	-12	-15	1			-9	2
**145**	-9	-2	-4	-45	-9	36	-9			-6	8

**151**	-4	-14	4	-51	-14	-9	-28			-17	6
**159**	-13	-18	-10	-33	-12	-8	-12			-15	3
**168**	-3	-8	-10	-43	0	-1	-26			-13	6
**175**	2	-15	-13	-40	-11	4	-6			-11	5
**Ear swelling**	**Conventional dose rate 33 Gy**										
**time after irradiation [d]**	Mouse ID: 8	26	34	40	48	54	62			mean	SEM
**0**	2	4	-6	-6	-15	-25	-26			-10	4
**2**	20	-3	-7	-23	-5	5	20			1	5
**4**	8	13	-13	12	-32	-32	32			-2	9
**6**	-8	25	-5	-11	25	5	10			6	5
**8**	11	-5	-10	-15	-10	-12	-4			-6	3
**10**	14	26	6	-2	41	6	13			15	5
**12**	41	6	-14	-9	-9	-4	6			2	7
**14**	62	12	2	-11	-6	6	17			12	9
**16**	64	14	6	22	21	7	69			29	9
**18**	100	20	42	60	37	17	135			59	15
**20**	208	36	138	114	76	11	136			103	23
**22**	226	83	308	188	80	20	145			150	35
**24**	331	85	81	245	150	20	176			155	37
**26**	417	79	62	184	149	9	124			146	46
**28**	298	71	41	91	106	-2	83			98	33
**30**	223	51	21	81	76	3	58			73	25
**32**	163	49	3	41	48	-4	46			49	19
**34**	121	35	6	41	30	-9	35			37	14
**36**	93	48	21	23	31	5	43			38	10
**38**	86	20	21	28	8	-4	38			28	10
**42**	75	15	9	12	24	-10	49			25	10
**46**	64	17	-11	7	7	-5	25			15	9
**50**	52	7	-2	3	3	2	25			13	7
**54**	60	-5	5	-6	5	0	19			11	8
**57**	56	5	-2	0	-4	-15	23			9	8
**61**	73	13	11	5	0	-5	20			17	9
**68**	67	-2	2	42	0	-13	25			17	10
**75**	31	-12	-5	0	-7	-2	8			2	5
**82**	37	9	-15	-11	-11	-15	14			1	7
**89**	21	5	-7	-7	-7	-14	1			-1	4
**96**	20	12	-3	-8	-21	-6	-3			-1	5
**103**	16	11	4	-19	-18	-8	-1			-2	5
**110**	-1	18	-4	-16	-7	-1	24			2	5
**117**	30	10	20	-7	-2	-15	31			9	6
**124**	5	2	20	-13	-3	-25	2			-2	5
**131**	-2	-1	-9	-19	-19	-7	8			-7	3
**138**	10	11	23	-14	-30	-10	31			3	8
**145**	13	-7	38	-14	-7	6	13			6	6

**151**	19	9	16	-34	-18	-21	24			-1	8
**159**	-7	-5	15	-20	-27	-5	10			-5	5
**168**	-5	4	92	-23	-13	-16	-1			5	14
**175**	19	-8	39	-15	-5	2	7			5	6
**Ear swelling**	**Flash9 dose rate 33 Gy**										
**time after irradiation [d]**	Mouse ID: 1	10	58							mean	SEM
**0**	5	0	-25							-6	8
**2**	-2	45	-10							11	14
**4**	-2	-7	-25							-11	6
**6**	10	-16	14							3	8
**8**	11	3	-12							1	6
**10**	-7	-9	6							-3	4
**12**	-2	13	-7							1	5
**14**	-8	2	1							-2	3
**16**	1	22	12							12	5
**18**	22	29	10							20	4
**20**	53	53	23							43	8
**22**	106	60	31							66	18
**24**	45	55	46							48	3
**26**	14	32	17							21	5
**28**	8	31	14							18	6
**30**	16	33	6							18	6
**32**	9	8	-11							2	5
**34**	8	16	-5							6	5
**36**	8.1	8.1	13.1							9.8	1.4
**38**	5	23	0							9	6
**42**	4	35	5							15	8
**46**	7	15	2							8	3
**50**	-3.3	1.7	-3.3							-1.7	1.4
**54**	-11	9	-3							-2	5
**57**	5	-14	-10							-6	5
**61**	18	11	1							10	4
**68**	0.0	-6.7	1.7							-1.7	2.1
**75**	-8.8	-7.1	-2.1							-6.0	1.6
**82**	0	-16	-10							-9	4
**89**	6.2	-2.1	2.9							2.3	2.0
**96**	0.5	-2.9	-9.5							-4.0	2.4
**103**	-11.0	-7.6	-12.6							-10.4	1.2
**110**	-10.7	-9.0	-4.0							-7.9	1.6
**117**	-15	8	-5							-4	6
**124**	-11.7	-1.7	-6.7							-6.7	2.4
**131**	-22	-1	-21							-15	6
**138**	20	10	-4							8	6

**145**	1.2	7.9	-0.5							2.9	2.1
**151**	-11	21	-13							-1	9
**159**	-7	-27	-8							-14	5
**168**	-9.5	-14.5	-12.9							-12.3	1.2
**175**	-3	-15	-3							-7	3
**Ear swelling**	**Flash930 dose rate 33 Gy**										
**time after irradiation [d]**	Mouse ID: 9	19	21	23	41	47	49	61	63	mean	SEM
**0**	-6	14	5	14	22	19	-1	-6	-6	6	4
**2**	65	55	18	-12	30	13	-3	3	0	19	8
**4**	5	13	22	0	0	-23	-20	-3	-5	-1	4
**6**	-6	-15	15	25	44	-1	4	-13	19	8	6
**8**	8	3	0	6	6	-27	-10	18	-14	-1	4
**10**	23	14	31	8	9	18	-4	-1	-14	9	4
**12**	39	-4	41	16	8	-14	-16	-12	6	7	7
**14**	34	11	44	16	12	1	2	-14	-1	12	6
**16**	34	11	47	11	21	44	12	11	11	22	5
**18**	60	42	77	85	74	20	10	27	20	46	9
**20**	134	81	81	94	49	34	31	14	61	64	12
**22**	96	201	116	115	136	55	13	18	90	93	19
**24**	100	255	111	131	108	35	16	3	60	91	24
**26**	81	217	82	101	77	24	29	6	61	75	19
**28**	58	159	74	96	53	13	-2	-2	46	55	16
**30**	48	151	48	69	24	13	-6	1	49	44	15
**32**	28	96	23	28	6	-4	-9	-11	38	22	10
**34**	30	68	26	13	21	-2	-14	-22	28	16	8
**36**	28	66	20	25	25	6	1	21	28	25	6
**38**	25	55	13	21	10	-5	-15	-10	20	13	7
**42**	19	42	22	-5	7	7	9	-10	20	12	5
**46**	2	34	20	10	-1	-6	-16	-11	22	6	5
**50**	20	-2	7	15	38	2	-8	-13	30	10	5
**54**	-8	19	-6	7	12	-3	-8	-8	9	2	3
**57**	5	18	3	6	-4	-17	-4	-17	31	3	5
**61**	18	13	11	6	1	-4	3	-12	28	7	4
**68**	13	-2	8	-2	7	-2	5	-20	13	2	3
**75**	5	0	6	-7	-10	-2	-5	-29	5	-4	3
**82**	-10	12	5	0	-10	-15	-1	-16	7	-3	3
**89**	-3.8	-17.1	-2.1	-10.5	-20.5	-15.5	-8.8	-8.8	-0.5	-9.7	2.2
**96**	12	-8	-21	0	-23	-5	-8	-28	-6	-10	4
**103**	-4	-9	-13	-3	21	-16	-16	2	-16	-6	4
**110**	-11	-6	-6	-6	-7	-9	-12	3	16	-4	3
**117**	5	5	0	-15	5	-5	-7	-12	31	0	4
**124**	17	7	-20	-35	-7	-3	2	-22	-8	-8	5

**131**	-7.4	-7.4	-24.0	-24.0	-14.0	-9.0	-17.4	-20.7	-9.0	-14.8	2.2
**138**	26	40	-22	-12	3	10	-14	-4	-4	2	6
**145**	13	31	-10	-7	13	0	-10	3	-5	3	4
**151**	21	52	-4	-14	6	-14	-18	-14	-21	-1	8
**159**	-5	50	-3	-3	-10	5	-10	-18	5	1	6
**168**	0	27	-11	-10	-16	-1	-6	-20	0	-4	4
**175**	9	12	-3	-16	-1	0	-8	-21	29	0	5
**Ear swelling**	**Sham irradiated 0 Gy**										
**time after irradiation [d]**	Mouse ID: 15	24	25	28	29	45	59			mean	SEM
**0**	-13	0	12	-8	9	-15	15			0	4
**2**	7	30	-13	2	-12	7	-20			0	6
**4**	13	-8	7	-7	-2	3	-7			0	3
**6**	-11	-6	-15	14	19	4	-5			0	4
**8**	-4	23	8	1	-15	-15	1			0	5
**10**	-1	-6	-17	19	11	3	-9			0	4
**12**	13	3	-11	6	-4	6	-12			0	3
**14**	7	7	-14	11	-3	-8	-1			0	3
**16**	22	-9	-19	1	2	6	-3			0	5
**18**	10	14	-21	20	-16	4	-11			0	6
**20**	9	4	-17	-2	21	3	-17			0	5
**22**	-2	11	-7	28	-19	-9	-4			0	5
**24**	0	10	-9	8	6	-7	-7			0	3
**26**	-11	-4	-11	36	14	-16	-8			0	6
**28**	-2	1	-17	49	-1	-16	-14			0	8
**30**	1	16	-19	26	-11	-2	-11			0	6
**32**	-2	8	-17	26	-2	-6	-6			0	5
**34**	-2	11	-15	16	-9	-5	5			0	4
**36**	1	8	-22	33	-14	-15	8			0	7
**38**	10	6	-20	26	0	-15	-7			0	6
**42**	9	-6	-13	29	-8	-6	-3			0	5
**46**	0	-6	-1	15	-3	-11	5			0	3
**50**	2	-5	-8	30	-15	-5	2			0	5
**54**	-6	-8	-16	29	0	7	-6			0	5
**57**	-10	-9	-20	30	35	-17	-9			0	8
**61**	-7	0	-17	36	3	1	-17			0	6
**68**	2	-7	-8	13	7	-12	5			0	3
**75**	-4	-4	-20	21	21	-7	-7			0	5
**82**	-3	-10	-21	32	2	-13	14			0	6
**89**	0	-15	-14	46	5	-10	-10			0	8
**96**	-1	-8	-10	37	7	-10	-16			0	6
**103**	-11	-8	-28	39	22	-16	1			0	8
**110**	-12	-9	-19	39	21	-4	-16			0	8
**117**	-4	-4	-7	5	20	-17	8			0	4

**124**	-10	2	-7	23	5	-7	-7			0	4
**131**	-16	-17	-11	39	9	1	-6			0	7
**138**	-10	-10	-20	48	10	-5	-10			0	8
**145**	3	0	-20	36	3	-12	-9			0	6
**151**	-14	-8	-21	31	22	-6	-4			0	7
**159**	0	-7	-27	40	3	-13	3			0	7
**168**	4	2	-16	27	-16	-6	5			0	5
**175**	0	0	-15	19	-10	-8	14			0	4
**ear thickness**	**Conventional dose rate 23 Gy**										
**time after irradiation [d]**	Mouse ID: 18	22	32	36	46	50	60			mean	SEM
**0**	270	250	270	245	230	260	290			247	4
**0**	270	225	240	210	240	230	245				
**0**	260	260	255	220	230	240	255				
**2**	250	245	250	215	215	220	240			239	3
**2**	245	225	250	230	235	225	250				
**2**	240	245	245	260	245	240	245				
**4**	225	230	240	235	225	200	245			227	3
**4**	230	225	235	235	225	200	235				
**4**	230	230	235	230	230	195	235				
**6**	245	230	255	250	245	245	250			244	2
**6**	245	230	250	240	240	240	250				
**6**	240	230	260	240	255	245	245				
**8**	240	225	250	245	240	230	240			236	3
**8**	230	225	250	270	240	220	230				
**8**	230	225	245	245	230	225	225				
**10**	255	235	245	260	265	250	245			249	2
**10**	260	235	260	250	265	240	245				
**10**	250	235	255	240	260	225	255				
**12**	295	255	250	245	235	225	240			244	4
**12**	270	240	250	245	235	225	240				
**12**	265	245	250	230	240	200	240				
**14**	280	230	245	250	255	230	245			247	3
**14**	260	230	245	265	255	230	240				
**14**	280	230	250	255	250	230	240				
**16**	260	250	260	275	265	250	265			259	2
**16**	255	255	270	275	260	245	255				
**16**	255	245	265	270	260	235	260				
**18**	280	280	255	270	265	250	255			264	2
**18**	280	260	255	260	265	250	255				
**18**	285	265	265	265	270	250	260				
**20**	290	375	300	280	290	260	255			288	7
**20**	285	355	290	270	295	260	270				
**20**	280	350	265	265	290	255	260				
**22**	310	345	310	305	290	270	270			301	6

**22**	305	355	295	330	285	270	265				
**22**	320	355	280	335	295	275	255				
**24**	280	320	290	330	280	275	280			291	4
**24**	280	325	280	315	285	275	280				
**24**	275	310	275	315	280	280	285				
**26**	270	310	325	310	270	280	250			287	5
**26**	270	305	310	310	270	285	250				
**26**	275	300	300	305	275	290	260				
**28**	260	300	300	310	270	260	260			278	4
**28**	260	280	295	310	260	265	260				
**28**	265	280	290	305	260	280	265				
**30**	260	285	310	305	265	260	260			272	4
**30**	255	270	285	305	265	260	260				
**30**	265	270	255	305	260	255	250				
**32**	250	265	270	300	260	265	265			265	3
**32**	245	260	270	280	270	255	265				
**32**	250	265	285	280	260	255	260				
**34**	240	250	250	270	240	245	245			247	2
**34**	240	255	240	270	240	240	240				
**34**	245	250	240	265	245	240	240				
**36**	255	265	255	275	255	275	260			260	2
**36**	250	265	250	270	250	260	255				
**36**	245	275	250	265	255	265	255				
**38**	260	260	255	265	240	240	240			252	2
**38**	250	260	260	255	250	240	240				
**38**	250	260	280	255	250	235	250				
**42**	250	250	255	270	240	240	245			247	2
**42**	240	250	240	260	240	240	245				
**42**	245	245	235	265	245	230	250				
**46**	250	245	250	265	245	245	255			252	1
**46**	250	250	255	265	245	250	255				
**46**	245	250	245	260	250	250	260				
**50**	230	260	255	250	255	250	255			246	2
**50**	225	250	260	250	250	250	235				
**50**	225	250	245	250	250	245	235				
**54**	230	260	255	255	255	250	245			249	1
**54**	240	255	250	250	250	250	250				
**54**	240	255	245	250	250	245	250				
**57**	255	250	245	260	240	245	245			246	2
**57**	240	255	245	260	235	240	245				
**57**	240	255	240	255	235	240	250				
**61**	260	255	250	250	240	245	250			250	1
**61**	255	255	255	250	245	245	245				
**61**	250	255	255	250	240	245	250				
**68**	245	245	245	240	230	240	240			238	1
**68**	240	240	235	230	230	235	240				

**68**	235	240	235	250	235	230	235				
**75**	240	250	240	250	240	240	245			242	1
**75**	230	250	240	250	240	235	245				
**75**	230	250	240	255	235	235	245				
**82**	245	240	240	250	240	245	240			240	1
**82**	240	240	240	250	235	220	240				
**82**	235	240	245	245	230	235	240				
**89**	265	255	230	240	245	255	235			244	2
**89**	255	230	240	240	255	250	245				
**89**	235	240	250	230	240	240	245				
**96**	250	240	245	240	255	245	240			244	1
**96**	240	240	245	240	255	250	240				
**96**	240	235	250	240	250	240	240				
**103**	255	250	235	240	230	235	240			238	2
**103**	255	245	235	235	235	225	235				
**103**	250	240	235	235	230	220	235				
**110**	230	225	225	225	220	245	220			227	2
**110**	225	225	220	235	220	240	225				
**110**	230	220	220	235	220	245	220				
**117**	245	235	240	250	240	225	235			237	1
**117**	245	235	240	240	245	225	235				
**117**	230	235	235	240	235	230	240				
**124**	240	245	255	260	240	250	240			245	1
**124**	240	245	255	240	240	250	240				
**124**	240	245	250	245	245	245	235				
**131**	250	245	240	250	250	250	245			243	1
**131**	250	245	235	240	240	250	245				
**131**	240	240	235	230	240	245	240				
**138**	235	230	230	240	250	220	245			236	2
**138**	235	225	230	240	245	230	260				
**138**	230	225	220	240	245	230	255				
**145**	235	225	215	220	240	250	220			230	2
**145**	230	225	215	225	240	245	220				
**145**	245	225	215	225	240	245	235				
**151**	230	235	230	230	220	235	215			228	1
**151**	230	230	230	230	225	235	215				
**151**	230	230	230	230	220	235	215				
**159**	250	235	225	225	235	250	235			234	2
**159**	250	230	220	230	230	245	230				
**159**	250	230	220	230	225	240	230				
**168**	265	250	240	250	230	250	235			246	2
**168**	265	245	240	250	240	250	235				
**168**	270	245	235	245	245	250	230				
**175**	240	205	240	240	240	270	220			234	3
**175**	230	215	230	240	240	260	220				
**175**	230	210	230	240	235	255	220				

**ear thickness**	**Flash9 dose rate 23 Gy**										
**time after irradiation [d]**	Mouse ID: 31	38	39	43	44	52	53	56	57	mean	SEM
**0**	230	220	235	250	250	280	260	235	240	243	3
**0**	225	265	240	265	245	225	255	240	235		
**0**	220	210	220	255	255	225	250	240	280		
**2**	230	260	250	230	240	270	235	250	250	245	3
**2**	230	270	235	245	240	245	220	250	250		
**2**	230	260	260	250	270	225	220	230	280		
**4**	270	250	230	220	245	240	235	270	235	239	2
**4**	240	250	225	225	235	235	235	255	235		
**4**	235	245	225	220	235	230	235	255	230		
**6**	230	250	255	255	250	250	255	250	240	246	2
**6**	240	250	240	245	250	245	230	240	270		
**6**	245	250	235	245	245	245	235	240	245		
**8**	235	250	230	240	230	230	240	230	265	238	2
**8**	235	240	230	235	230	235	245	230	245		
**8**	240	235	230	240	230	255	245	230	245		
**10**	245	255	230	240	235	250	255	245	255	246	2
**10**	270	250	225	245	225	245	230	245	250		
**10**	255	250	245	245	235	265	245	250	250		
**12**	255	245	240	250	240	250	250	240	240	245	1
**12**	260	240	240	245	240	240	250	260	240		
**12**	255	245	240	245	235	230	255	245	250		
**14**	260	250	250	250	250	245	240	240	250	250	2
**14**	285	255	250	250	250	240	240	240	255		
**14**	270	255	250	250	255	230	240	240	260		
**16**	265	245	255	250	225	255	255	240	270	252	2
**16**	265	245	255	255	255	230	255	250	270		
**16**	265	240	250	245	245	240	260	245	270		
**18**	275	260	260	255	265	240	260	250	245	260	3
**18**	275	250	285	250	290	240	260	250	245		
**18**	280	265	270	250	270	245	265	270	250		
**20**	260	255	275	255	290	230	305	265	250	262	4
**20**	260	250	275	250	270	235	280	260	250		
**20**	270	250	265	255	265	235	315	255	250		
**22**	280	255	270	270	290	280	255	275	260	275	3
**22**	310	255	295	280	290	270	265	285	265		
**22**	310	250	305	260	275	255	260	295	270		
**24**	380	260	290	260	300	290	300	270	265	292	8
**24**	405	260	275	260	305	285	310	340	235		
**24**	375	250	305	255	290	265	290	325	235		
**26**	310	310	280	260	270	240	275	365	260	277	5
**26**	290	300	280	265	265	260	270	300	260		
**26**	295	250	270	255	280	245	270	300	265		

**28**	340	275	260	260	280	265	265	290	250	272	5
**28**	330	265	250	255	280	270	265	290	250		
**28**	315	255	250	250	275	260	260	295	255		
**30**	320	245	270	250	270	250	300	250	265	261	4
**30**	300	235	255	245	265	230	265	250	260		
**30**	295	255	265	255	255	225	270	255	255		
**32**	295	245	240	240	255	240	260	260	255	255	3
**32**	290	245	245	255	260	230	265	250	255		
**32**	280	245	250	250	260	230	265	265	250		
**34**	275	240	250	240	255	245	280	255	255	251	3
**34**	275	240	250	235	255	240	270	250	260		
**34**	270	230	240	225	260	240	260	245	250		
**36**	300	245	240	255	230	240	245	235	260	251	3
**36**	285	240	245	250	245	240	255	240	260		
**36**	285	240	245	245	245	250	250	240	260		
**38**	250	240	235	225	240	230	240	230	245	241	2
**38**	250	240	240	220	240	230	250	240	250		
**38**	260	240	240	235	245	235	255	240	250		
**42**	245	245	225	235	245	250	265	250	260	247	3
**42**	245	240	225	230	245	245	270	250	280		
**42**	240	240	225	245	245	250	255	245	275		
**46**	245	250	230	230	240	235	280	230	250	244	3
**46**	260	250	230	245	230	235	265	240	250		
**46**	250	245	230	235	235	235	275	235	260		
**50**	240	220	230	250	235	235	255	225	255	239	2
**50**	240	220	230	250	245	235	255	230	255		
**50**	245	240	225	240	230	230	260	240	245		
**54**	245	245	235	250	250	235	275	250	255	249	2
**54**	240	240	230	250	255	240	275	250	260		
**54**	240	240	230	255	245	240	275	245	260		
**57**	240	240	235	235	235	240	270	235	240	241	2
**57**	240	245	230	240	230	240	260	235	255		
**57**	250	245	230	240	230	235	260	240	245		
**61**	255	245	235	245	240	240	250	235	250	243	1
**61**	250	240	235	255	250	245	245	235	245		
**61**	250	230	240	225	250	245	245	240	245		
**68**	240	240	230	245	245	230	255	235	255	240	2
**68**	240	255	235	250	240	230	255	230	255		
**68**	240	240	235	235	235	230	240	225	240		
**75**	240	245	235	240	230	270	250	245	260	244	2
**75**	240	245	235	240	230	240	255	240	260		
**75**	240	240	235	235	225	255	240	250	255		
**82**	255	225	235	250	230	235	265	240	255	245	2
**82**	250	235	240	250	240	240	265	240	255		
**82**	245	245	225	270	240	240	255	235	250		
**89**	240	235	230	250	245	240	250	235	240	241	1

**89**	240	235	230	235	245	245	250	240	255		
**89**	235	230	230	245	255	235	245	240	250		
**96**	250	250	240	250	240	235	255	240	255	244	2
**96**	250	250	240	250	230	235	245	225	250		
**96**	250	250	235	240	255	235	250	235	250		
**103**	275	235	220	240	230	235	245	235	245	237	2
**103**	255	230	225	235	220	235	235	235	245		
**103**	250	230	230	235	220	230	250	235	250		
**110**	225	220	225	220	215	235	245	220	225	226	1
**110**	230	225	220	230	210	235	235	220	225		
**110**	230	225	225	225	215	225	230	225	240		
**117**	240	250	250	260	235	250	260	255	285	250	2
**117**	245	250	240	245	235	250	260	245	260		
**117**	255	250	230	245	235	245	265	245	260		
**124**	235	255	220	240	240	245	240	235	275	244	3
**124**	240	255	220	240	245	245	245	230	275		
**124**	245	250	230	240	245	245	245	230	265		
**131**	230	245	230	240	245	235	255	235	240	236	2
**131**	220	240	230	240	235	220	255	230	250		
**131**	215	245	225	235	240	220	250	225	250		
**138**	235	260	225	230	225	235	250	210	250	236	3
**138**	235	255	215	235	230	230	245	225	250		
**138**	235	250	210	235	230	230	245	225	260		
**145**	230	235	220	230	225	240	260	225	240	235	2
**145**	235	235	220	235	230	235	260	235	240		
**145**	235	245	220	210	230	230	260	235	245		
**151**	245	225	230	235	215	225	220	240	230	231	2
**151**	240	225	230	240	220	225	225	230	240		
**151**	245	225	230	240	220	230	225	240	240		
**159**	235	230	225	230	225	230	265	230	245	233	3
**159**	235	230	225	225	220	230	270	225	245		
**159**	235	225	220	225	220	225	255	225	250		
**168**	250	250	235	235	240	235	245	270	250	242	2
**168**	250	245	235	240	240	235	245	240	245		
**168**	245	240	230	240	230	230	245	240	245		
**175**	235	250	235	245	230	230	250	235	255	242	2
**175**	235	250	235	245	240	235	255	235	250		
**175**	240	250	235	240	250	240	260	235	245		
**ear thickness**	**Flash930 dose rate 23 Gy**										
**time after irradiation [d]**	Mouse ID: 5	7	13	17	35	37	55			mean	SEM
**0**	250	265	290	280	250	250	250			255	4
**0**	245	250	275	290	245	265	235				
**0**	245	245	260	240	255	220	255				
**2**	275	255	240	200	235	240	235			242	4

**2**	265	255	235	195	235	240	235				
**2**	255	255	235	215	240	270	265				
**4**	260	240	230	220	250	255	205			238	4
**4**	250	235	235	280	240	245	200				
**4**	235	230	235	250	240	250	210				
**6**	275	235	235	280	225	240	240			241	4
**6**	265	230	230	275	230	240	235				
**6**	260	225	225	215	220	240	245				
**8**	280	245	245	200	215	270	230			244	6
**8**	275	240	300	195	215	290	225				
**8**	285	240	240	240	225	240	235				
**10**	240	235	240	235	230	265	245			235	3
**10**	245	225	230	225	240	255	240				
**10**	260	225	230	195	220	235	230				
**12**	280	255	260	215	225	250	230			245	5
**12**	265	250	270	205	230	250	235				
**12**	270	260	265	200	240	260	225				
**14**	250	265	275	230	260	270	235			246	4
**14**	255	255	265	215	260	250	230				
**14**	245	245	270	190	240	240	230				
**16**	280	290	265	215	235	250	230			245	6
**16**	275	270	260	200	215	240	230				
**16**	275	260	260	200	230	240	225				
**18**	270	265	285	245	230	260	230			253	4
**18**	280	265	280	230	230	245	250				
**18**	285	240	280	225	230	250	235				
**20**	290	280	325	250	255	255	240			270	6
**20**	280	285	320	230	260	270	250				
**20**	275	275	315	235	260	260	250				
**22**	280	270	380	220	360	275	250			282	10
**22**	280	270	360	220	300	280	255				
**22**	295	265	360	220	280	270	240				
**24**	295	275	350	215	260	255	290			281	8
**24**	305	275	345	250	260	255	285				
**24**	310	275	375	235	265	255	275				
**26**	330	270	340	225	305	255	250			280	8
**26**	310	270	340	220	310	260	260				
**26**	310	280	320	260	260	250	255				
**28**	290	255	320	210	270	255	245			261	7
**28**	280	255	320	225	270	260	235				
**28**	275	260	305	205	255	260	235				
**30**	290	250	315	215	270	255	230			258	6
**30**	270	250	305	215	260	255	230				
**30**	305	265	290	215	255	240	230				
**32**	265	250	295	220	240	250	245			251	5
**32**	270	250	315	220	235	250	235				

**32**	270	245	290	225	230	245	235				
**34**	265	240	300	205	235	225	240			246	5
**34**	265	240	285	210	270	225	240				
**34**	260	235	285	225	250	220	240				
**36**	260	240	280	200	240	235	245			240	5
**36**	245	230	280	200	235	235	240				
**36**	255	235	275	205	240	240	235				
**38**	250	240	280	200	225	245	235			239	5
**38**	245	235	280	200	215	245	235				
**38**	250	235	270	220	240	235	230				
**42**	255	245	280	210	250	250	235			244	4
**42**	250	245	280	205	250	245	235				
**42**	250	245	270	200	240	245	235				
**46**	250	240	270	210	230	245	230			237	4
**46**	250	230	270	210	225	250	230				
**46**	245	230	270	205	220	240	230				
**50**	235	230	260	210	230	240	235			236	3
**50**	235	225	260	220	230	240	235				
**50**	245	245	265	215	225	245	235				
**54**	240	230	255	205	250	255	240			237	4
**54**	230	225	255	205	240	240	235				
**54**	250	225	260	200	255	240	250				
**57**	240	235	250	220	250	240	220			236	3
**57**	235	235	250	215	250	240	225				
**57**	245	235	250	210	235	245	225				
**61**	245	230	255	210	250	240	220			236	3
**61**	245	230	255	220	250	240	220				
**61**	245	230	260	210	250	245	215				
**68**	240	240	235	205	245	225	235			231	3
**68**	240	235	240	205	230	230	245				
**68**	230	230	240	190	245	230	240				
**75**	245	225	260	195	260	240	225			237	4
**75**	250	220	260	220	245	240	225				
**75**	250	230	260	205	255	245	230				
**82**	250	230	270	200	240	240	230			236	4
**82**	250	235	270	200	240	225	230				
**82**	250	230	265	195	245	230	230				
**89**	255	230	265	205	240	230	245			237	3
**89**	240	225	260	235	250	225	245				
**89**	250	225	255	225	235	215	230				
**96**	240	240	250	210	235	250	240			237	3
**96**	240	240	250	205	235	225	245				
**96**	240	250	245	230	240	230	240				
**103**	215	225	235	190	235	230	225			220	3
**103**	220	225	230	190	220	230	225				
**103**	215	225	225	190	215	225	230				

**110**	230	225	225	195	230	220	215			217	2
**110**	225	215	225	185	220	220	215				
**110**	225	220	225	200	210	215	220				
**117**	230	245	250	230	245	245	250			239	3
**117**	230	240	250	205	245	245	245				
**117**	225	235	245	205	250	250	245				
**124**	245	235	245	245	245	240	215			236	3
**124**	245	240	250	195	245	240	215				
**124**	250	230	240	235	255	235	205				
**131**	235	225	235	200	225	245	240			231	3
**131**	235	220	230	230	230	240	265				
**131**	235	220	230	220	230	235	235				
**138**	230	225	225	225	225	220	235			225	1
**138**	225	225	230	220	220	220	235				
**138**	225	220	235	220	220	215	235				
**145**	225	230	230	185	220	265	220			226	5
**145**	225	230	225	185	225	265	225				
**145**	220	230	230	190	225	275	225				
**151**	225	215	240	180	220	225	205			216	4
**151**	230	220	235	180	215	225	205				
**151**	230	220	235	185	220	220	205				
**159**	225	220	230	205	225	230	225			221	2
**159**	225	220	225	205	230	230	225				
**159**	220	215	225	200	220	225	225				
**168**	250	240	240	200	250	245	220			235	3
**168**	245	245	240	210	250	245	220				
**168**	240	235	235	205	245	250	225				
**175**	245	235	230	210	235	250	230			232	3
**175**	240	230	230	200	230	245	240				
**175**	250	220	230	200	230	245	240				
**ear thickness**	**Conventional dose rate 33 Gy**										
**time after irradiation [d]**	Mouse ID: 8	26	34	40	48	54	62			mean	SEM
**0**	245	265	250	245	230	225	210			236	3
**0**	240	240	225	240	235	220	230				
**0**	260	245	245	235	230	220	220				
**2**	265	240	230	215	245	250	265			239	3
**2**	255	225	230	215	230	230	255				
**2**	255	240	235	215	225	250	255				
**4**	255	265	225	265	210	210	285			240	5
**4**	250	255	225	250	210	210	270				
**4**	245	245	235	245	210	210	265				
**6**	235	265	240	235	265	250	225			247	4
**6**	235	265	240	230	265	250	275				
**6**	230	270	230	225	270	240	255				

**8**	265	240	240	215	230	235	240			239	3
**8**	270	240	235	235	255	230	240				
**8**	235	240	230	240	220	235	245				
**10**	240	270	245	240	275	245	240			252	3
**10**	240	265	245	235	275	245	255				
**10**	275	255	240	230	285	240	255				
**12**	285	250	230	235	240	235	255			246	4
**12**	290	255	230	235	235	235	250				
**12**	280	245	230	235	230	250	245				
**14**	305	260	250	240	245	250	270			258	5
**14**	300	260	255	235	245	250	260				
**14**	320	255	240	230	230	255	260				
**16**	320	265	240	275	265	270	350			275	6
**16**	310	260	255	270	265	250	305				
**16**	300	255	260	260	270	240	290				
**18**	350	270	290	330	280	265	370			302	9
**18**	345	260	290	290	280	260	375				
**18**	335	260	275	290	280	255	390				
**20**	490	290	340	340	320	260	370			352	15
**20**	450	285	370	375	305	255	355				
**20**	430	280	450	375	350	265	430				
**22**	490	340	610	450	350	270	380			402	20
**22**	480	335	560	440	320	265	420				
**22**	465	330	510	430	325	280	390				
**24**	610	335	320	520	425	270	430			407	22
**24**	580	345	360	490	400	270	430				
**24**	560	330	320	480	380	275	425				
**26**	700	330	315	420	420	265	370			399	27
**26**	660	330	310	490	400	260	370				
**26**	650	335	320	400	385	260	390				
**28**	550	325	305	350	370	250	345			349	19
**28**	550	325	280	300	360	250	335				
**28**	545	315	290	375	340	245	320				
**30**	480	305	285	330	330	250	310			320	15
**30**	470	300	265	330	320	250	305				
**30**	460	290	255	325	320	250	300				
**32**	400	305	260	300	305	250	300			302	11
**32**	420	300	250	280	300	250	300				
**32**	425	300	255	300	295	245	295				
**34**	375	290	270	300	280	245	285			286	8
**34**	360	280	250	290	280	245	285				
**34**	375	280	245	280	275	230	280				
**36**	330	290	255	280	280	240	290			280	6
**36**	330	290	275	250	270	255	285				
**36**	345	290	260	265	270	245	280				
**38**	335	285	260	280	255	240	285			274	6

**38**	335	260	260	280	255	240	290				
**38**	325	250	280	260	250	245	275				
**42**	325	265	250	265	260	240	290			270	6
**42**	320	260	250	250	275	235	290				
**42**	315	255	260	255	270	230	300				
**46**	310	265	235	260	255	245	270			261	5
**46**	310	265	230	255	255	240	270				
**46**	310	260	240	245	250	240	275				
**50**	300	260	245	245	250	245	270			258	4
**50**	290	245	245	245	250	240	270				
**50**	300	250	240	255	245	255	270				
**54**	310	245	255	250	270	255	270			263	5
**54**	310	245	255	245	260	250	275				
**54**	315	250	260	240	240	250	265				
**57**	305	255	245	255	250	235	275			259	5
**57**	300	260	255	245	250	235	275				
**57**	315	250	245	250	240	235	270				
**61**	330	260	270	260	250	250	270			265	5
**61**	330	260	260	250	250	240	270				
**61**	305	265	250	250	245	240	265				
**68**	310	245	255	290	245	225	275			259	6
**68**	310	245	240	280	240	230	265				
**68**	305	230	235	280	240	230	260				
**75**	285	240	240	255	245	240	260			252	3
**75**	290	240	250	245	245	250	250				
**75**	270	235	245	250	240	255	265				
**82**	290	260	225	240	240	230	265			251	4
**82**	295	260	235	240	240	230	265				
**82**	275	255	245	235	235	245	260				
**89**	285	255	250	245	245	230	260			253	3
**89**	280	265	250	240	245	235	260				
**89**	260	255	240	255	250	255	245				
**96**	280	270	235	245	240	240	255			252	3
**96**	270	265	260	245	225	240	250				
**96**	270	260	255	245	230	260	245				
**103**	250	260	245	230	230	235	245			240	3
**103**	265	250	245	220	225	235	240				
**103**	260	250	250	220	220	235	240				
**110**	240	260	225	215	225	230	255			236	3
**110**	235	250	235	215	230	235	275				
**110**	225	245	230	225	225	235	245				
**117**	270	255	265	235	245	230	280			253	4
**117**	270	255	270	235	240	230	270				
**117**	280	250	255	240	240	225	275				
**124**	250	250	270	230	245	230	250			246	3
**124**	250	250	280	235	245	220	250				

**124**	260	250	255	240	245	220	250				
**131**	260	250	235	230	230	245	255			240	2
**131**	240	245	230	230	230	240	255				
**131**	235	245	250	225	225	235	255				
**138**	250	235	265	215	205	230	265			237	5
**138**	240	240	255	220	200	220	265				
**138**	240	260	250	225	205	220	265				
**145**	250	225	255	220	225	220	245			238	4
**145**	245	225	280	220	225	245	245				
**145**	240	225	275	215	225	250	245				
**151**	260	235	255	200	215	200	265			232	5
**151**	250	240	245	200	215	205	250				
**151**	245	250	245	195	215	230	255				
**159**	230	235	260	225	210	240	250			231	3
**159**	235	230	245	215	210	230	245				
**159**	225	230	250	210	210	225	245				
**168**	245	260	350	220	240	230	250			253	8
**168**	245	245	370	225	230	235	245				
**168**	240	250	300	230	235	230	245				
**175**	260	250	275	225	235	245	250			249	4
**175**	255	230	290	230	240	240	250				
**175**	270	225	280	230	240	250	250				
**ear thickness**	**Flash9 dose rate 33 Gy**										
**time after irradiation [d]**	Mouse ID: 1	10	58							mean	SEM
**0**	265	240	220							240	6
**0**	250	230	220								
**0**	240	270	225								
**2**	235	290	240							249	8
**2**	240	280	230								
**2**	235	280	215								
**4**	270	250	215							231	6
**4**	235	230	220								
**4**	215	225	215								
**6**	255	230	250							244	5
**6**	255	230	245								
**6**	245	215	270								
**8**	260	250	230							246	3
**8**	255	245	240								
**8**	255	250	230								
**10**	235	225	245							234	3
**10**	225	230	250								
**10**	230	230	235								
**12**	245	270	245							245	4
**12**	245	260	240								

**12**	235	240	225								
**14**	235	250	245							244	3
**14**	255	250	245								
**14**	225	245	250								
**16**	255	285	260							258	4
**16**	230	255	260								
**16**	255	265	255								
**18**	260	275	255							263	3
**18**	280	270	255								
**18**	255	270	250								
**20**	320	290	280							292	7
**20**	315	300	265								
**20**	270	315	270								
**22**	325	310	300							318	12
**22**	390	325	275								
**22**	360	300	275								
**24**	305	315	295							301	3
**24**	295	305	310								
**24**	290	300	290								
**26**	270	290	270							274	3
**26**	265	285	270								
**26**	265	280	270								
**28**	260	280	270							268	4
**28**	260	275	265								
**28**	255	290	260								
**30**	260	290	260							266	4
**30**	270	270	255								
**30**	260	280	245								
**32**	265	255	245							254	3
**32**	260	260	240								
**32**	260	265	240								
**34**	260	265	245							255	3
**34**	255	265	245								
**34**	255	265	240								
**36**	260	250	260							252	2
**36**	245	250	250								
**36**	245	250	255								
**38**	255	270	240							254	4
**38**	250	270	245								
**38**	245	265	250								
**42**	250	280	250							259	5
**42**	250	275	250								
**42**	245	285	250								
**46**	250	260	250							254	2
**46**	250	260	245								
**46**	260	265	250								

**50**	250	250	240							243	2
**50**	240	245	240								
**50**	235	245	245								
**54**	240	260	250							249	3
**54**	245	260	250								
**54**	235	260	245								
**57**	265	245	240							244	3
**57**	250	240	240								
**57**	250	225	240								
**61**	270	260	250							259	2
**61**	270	260	250								
**61**	260	260	250								
**68**	240	230	240							240	2
**68**	240	235	240								
**68**	245	240	250								
**75**	250	240	255							244	2
**75**	240	240	245								
**75**	235	250	245								
**82**	250	240	245							241	4
**82**	250	240	250								
**82**	250	220	225								
**89**	260	255	250							256	2
**89**	260	250	255								
**89**	260	250	265								
**96**	255	255	250							249	2
**96**	255	255	245								
**96**	250	240	235								
**103**	235	240	235							232	1
**103**	230	235	230								
**103**	230	230	225								
**110**	225	225	230							226	1
**110**	225	230	230								
**110**	220	220	230								
**117**	230	250	240							239	3
**117**	230	250	240								
**117**	225	255	235								
**124**	235	245	250							242	2
**124**	235	245	240								
**124**	240	250	235								
**131**	230	250	225							233	3
**131**	225	245	225								
**131**	220	245	230								
**138**	265	245	230							242	4
**138**	250	245	230								
**138**	245	240	230								
**145**	235	240	230							235	1

**145**	235	240	230								
**145**	230	240	235								
**151**	225	260	220							232	5
**151**	220	250	220								
**151**	220	250	220								
**159**	230	210	230							223	3
**159**	230	210	230								
**159**	230	210	225								
**168**	240	235	235							236	1
**168**	240	235	240								
**168**	235	230	230								
**175**	245	230	240							236	2
**175**	240	230	240								
**175**	235	225	240								
**ear thickness**	**Flash930 dose rate 33 Gy**										
**time after irradiation [d]**	Mouse ID: 9	19	21	23	41	47	49	61	63	mean	SEM
**0**	260	250	220	270	275	270	255	230	245	252	3
**0**	245	285	285	260	270	270	240	250	235		
**0**	215	245	250	250	260	255	240	240	240		
**2**	335	310	265	230	275	245	225	250	240	257	5
**2**	290	285	255	225	270	245	240	240	245		
**2**	285	285	250	225	260	265	240	235	230		
**4**	250	250	270	245	250	220	225	240	240	240	3
**4**	250	255	260	235	240	220	220	240	240		
**4**	240	260	260	245	235	215	220	235	230		
**6**	240	230	255	285	295	245	240	230	275	249	4
**6**	235	225	260	250	275	235	250	230	255		
**6**	230	225	255	265	285	240	245	225	250		
**8**	255	255	250	235	270	220	230	285	245	244	4
**8**	250	255	245	225	245	215	235	265	220		
**8**	255	235	240	295	240	220	240	240	230		
**10**	255	265	275	240	250	255	240	240	235	247	3
**10**	255	270	260	235	245	255	235	235	225		
**10**	270	220	270	260	245	255	225	235	210		
**12**	290	245	295	255	265	225	230	235	255	251	4
**12**	280	240	280	255	235	230	230	230	250		
**12**	280	235	280	270	255	235	225	230	245		
**14**	300	255	300	265	255	240	250	220	250	258	4
**14**	275	255	275	265	260	255	250	240	250		
**14**	265	260	295	255	260	245	245	235	235		
**16**	280	255	300	255	285	295	260	265	265	268	3
**16**	285	255	290	255	255	290	260	255	260		
**16**	275	260	290	260	260	285	255	250	245		
**18**	300	290	330	395	325	265	260	260	275	289	6

**18**	300	285	320	300	315	255	250	270	260		
**18**	310	280	310	290	310	270	250	280	255		
**20**	365	325	330	340	320	280	260	255	280	314	7
**20**	370	340	330	350	300	280	310	275	300		
**20**	415	325	330	340	275	290	270	260	350		
**22**	350	450	380	350	405	290	270	255	360	345	11
**22**	360	450	365	350	400	320	265	260	345		
**22**	335	460	360	400	360	310	260	295	320		
**24**	360	520	370	370	380	300	260	250	330	343	14
**24**	350	520	360	390	340	290	285	255	305		
**24**	345	480	360	390	360	270	260	260	300		
**26**	330	475	340	370	350	275	280	260	310	328	11
**26**	330	475	335	345	330	275	280	260	315		
**26**	340	460	330	345	310	280	285	255	315		
**28**	315	420	330	330	320	265	250	255	300	306	10
**28**	300	400	325	320	305	265	250	245	305		
**28**	310	410	320	390	285	260	245	245	285		
**30**	290	400	305	305	270	270	245	260	300	291	9
**30**	300	405	290	305	270	260	240	245	300		
**30**	295	390	290	340	275	250	240	240	290		
**32**	275	350	275	280	260	255	240	240	290	274	6
**32**	280	350	270	280	260	245	245	240	295		
**32**	285	345	280	280	255	245	245	245	285		
**34**	285	310	280	265	270	250	235	235	285	265	5
**34**	275	325	270	260	265	245	235	225	270		
**34**	275	315	275	260	275	245	235	220	275		
**36**	260	305	265	270	260	245	245	255	265	266	3
**36**	280	310	260	265	270	250	245	275	275		
**36**	270	310	260	265	270	250	240	260	270		
**38**	270	315	265	260	255	240	220	235	270	258	4
**38**	270	300	255	270	255	240	230	230	265		
**38**	270	285	255	270	255	240	240	240	260		
**42**	260	295	270	235	255	250	260	240	260	257	3
**42**	270	285	265	230	250	250	255	230	270		
**42**	260	280	265	255	250	255	245	235	265		
**46**	245	280	270	260	235	240	230	240	275	252	3
**46**	245	280	265	255	240	240	230	230	265		
**46**	255	280	265	255	260	240	230	235	265		
**50**	265	245	250	260	280	245	235	240	280	255	3
**50**	265	245	250	255	280	245	235	235	280		
**50**	265	240	255	265	290	250	240	220	265		
**54**	240	270	255	265	270	250	245	240	265	253	2
**54**	245	275	240	260	265	250	245	245	250		
**54**	245	265	240	250	255	245	240	245	265		
**57**	255	260	260	260	245	245	250	225	290	253	3
**57**	260	280	250	255	250	235	245	245	270		

**57**	250	265	250	255	245	220	245	230	285		
**61**	265	265	260	255	245	245	250	235	275	256	2
**61**	265	260	260	255	250	245	250	235	290		
**61**	270	260	260	255	255	245	255	240	265		
**68**	250	245	255	245	245	240	245	225	265	244	2
**68**	255	235	250	240	250	240	245	225	255		
**68**	260	240	245	235	250	240	250	215	245		
**75**	255	250	260	245	240	245	245	220	250	246	2
**75**	250	245	255	245	240	245	245	220	260		
**75**	260	255	255	240	240	255	245	225	255		
**82**	245	270	260	250	235	240	250	240	265	247	2
**82**	235	270	255	250	245	230	250	230	260		
**82**	240	245	250	250	240	235	245	230	245		
**89**	250	240	260	250	235	240	245	255	245	244	2
**89**	250	240	250	250	235	240	245	245	260		
**89**	250	230	245	230	230	235	245	235	255		
**96**	280	255	230	255	235	255	245	230	250	243	3
**96**	260	245	230	255	230	250	245	220	245		
**96**	255	235	235	250	225	240	245	225	245		
**103**	240	240	225	245	265	230	220	250	220	237	2
**103**	240	235	235	240	260	225	225	245	230		
**103**	235	225	230	235	265	225	235	240	230		
**110**	225	235	235	235	230	225	220	240	240	230	2
**110**	220	225	225	225	225	225	220	235	255		
**110**	225	225	225	225	225	225	225	235	255		
**117**	250	250	240	225	255	245	240	225	280	244	3
**117**	250	250	240	230	245	235	235	230	275		
**117**	245	245	250	230	245	235	235	240	270		
**124**	265	250	230	210	245	245	250	220	240	241	3
**124**	270	260	230	210	240	250	250	235	245		
**124**	260	255	225	220	240	240	250	225	235		
**131**	255	250	230	245	240	250	230	230	240	233	2
**131**	235	240	220	220	230	235	230	225	240		
**131**	230	230	220	205	230	230	230	225	235		
**138**	255	275	210	225	240	240	225	230	225	236	4
**138**	260	275	210	220	235	245	220	230	230		
**138**	265	270	215	220	235	245	215	230	235		
**145**	245	270	220	220	250	235	225	235	225	235	3
**145**	240	260	220	225	245	230	220	235	230		
**145**	250	260	225	230	240	230	220	235	225		
**151**	245	295	230	215	240	215	220	215	215	232	4
**151**	255	280	225	220	240	220	215	220	210		
**151**	260	280	230	220	235	220	210	220	210		
**159**	245	290	230	235	230	245	230	220	245	238	4
**159**	225	290	235	235	225	245	225	220	240		
**159**	225	280	235	230	225	235	225	215	240		

**168**	245	275	235	250	235	245	250	230	245	244	3
**168**	250	275	235	230	230	245	240	230	250		
**168**	250	275	240	235	230	250	235	225	250		
**175**	250	245	240	230	250	245	245	220	275	243	3
**175**	255	260	240	230	240	245	240	225	275		
**175**	250	260	240	220	235	240	220	220	265		
**ear thickness**	**Sham irradiated 0 Gy**										
**time after irradiation [d]**	Mouse ID: 15	24	25	28	29	45	59			mean	SEM
**0**	240	260	270	240	250	230	270			246	3
**0**	230	240	265	240	265	225	265				
**0**	230	240	240	235	250	240	250				
**2**	250	270	225	260	225	240	220			238	4
**2**	240	270	225	230	230	260	220				
**2**	245	265	225	230	225	235	215				
**4**	250	230	225	240	235	265	245			242	3
**4**	255	240	265	235	235	235	230				
**4**	260	230	255	230	250	235	230				
**6**	235	245	230	235	225	245	235			241	4
**6**	230	235	225	275	285	245	245				
**6**	225	225	225	255	270	245	230				
**8**	260	290	250	250	225	230	240			245	4
**8**	225	290	250	250	230	230	265				
**8**	240	225	260	240	235	230	235				
**10**	235	230	220	255	240	245	235			237	3
**10**	240	235	220	260	265	235	230				
**10**	235	230	220	255	240	240	220				
**12**	260	260	245	245	250	245	225			244	2
**12**	260	235	230	250	230	250	235				
**12**	250	245	225	255	240	255	235				
**14**	260	255	230	255	240	240	245			246	2
**14**	245	255	230	255	255	240	250				
**14**	255	250	235	260	235	235	240				
**16**	270	240	225	255	250	245	245			246	3
**16**	265	240	230	245	255	245	245				
**16**	270	230	225	240	240	265	240				
**18**	255	260	225	260	230	245	235			243	3
**18**	255	255	220	270	225	245	230				
**18**	250	255	220	260	225	250	230				
**20**	265	255	235	255	275	260	235			249	3
**20**	255	255	230	250	280	250	230				
**20**	255	250	230	235	255	245	230				
**22**	250	270	240	275	235	255	250			252	3
**22**	250	260	255	290	235	240	250				
**22**	250	260	240	275	230	235	245				

**24**	250	265	255	255	260	250	250			252	2
**24**	250	260	240	255	250	245	245				
**24**	255	260	235	270	265	240	240				
**26**	245	250	245	290	260	235	250			253	4
**26**	240	250	240	285	260	240	245				
**26**	240	245	240	290	280	235	240				
**28**	245	255	230	305	240	230	240			251	5
**28**	250	250	230	305	240	240	235				
**28**	250	250	240	290	270	235	235				
**30**	250	265	230	280	240	245	240			247	3
**30**	250	265	230	275	240	245	235				
**30**	245	260	225	265	230	245	235				
**32**	250	260	235	280	260	250	245			252	3
**32**	250	260	235	280	245	245	245				
**32**	250	260	235	275	245	245	250				
**34**	250	265	230	255	240	245	255			249	3
**34**	245	255	230	265	245	250	255				
**34**	245	260	240	275	235	235	250				
**36**	240	250	220	270	230	245	255			242	4
**36**	245	250	220	280	230	220	250				
**36**	245	250	220	275	225	215	245				
**38**	255	255	225	270	240	230	240			245	3
**38**	255	255	225	275	245	230	240				
**38**	255	245	225	270	250	230	235				
**42**	255	235	230	280	245	235	245			245	3
**42**	255	240	235	270	235	240	240				
**42**	250	240	230	270	230	240	240				
**46**	245	240	245	255	240	235	250			246	2
**46**	240	240	245	270	240	235	250				
**46**	255	240	245	260	250	235	255				
**50**	255	245	240	280	230	240	255			245	3
**50**	240	245	235	275	230	240	240				
**50**	245	230	235	270	230	240	245				
**54**	240	240	245	285	260	270	245			251	3
**54**	250	245	230	285	250	255	245				
**54**	245	245	230	270	245	250	245				
**57**	240	240	230	275	250	230	240			250	6
**57**	240	240	230	285	265	230	240				
**57**	240	245	230	280	340	240	245				
**61**	240	250	230	285	250	250	240			249	4
**61**	240	250	230	285	260	250	220				
**61**	245	245	235	285	245	250	235				
**68**	230	240	235	255	255	230	245			242	2
**68**	250	230	235	255	245	230	245				
**68**	250	235	230	255	245	230	250				
**75**	245	245	230	270	275	245	245			250	3

**75**	245	245	230	270	270	245	245				
**75**	250	250	230	275	270	240	240				
**82**	240	240	230	285	250	235	265			250	4
**82**	250	250	230	280	250	235	265				
**82**	250	230	225	280	255	240	260				
**89**	255	230	240	295	260	240	240			254	4
**89**	255	235	245	300	260	245	240				
**89**	250	250	235	305	255	245	250				
**96**	255	250	240	290	260	250	235			253	4
**96**	250	245	240	290	260	240	235				
**96**	250	240	250	290	260	240	240				
**103**	230	240	220	285	270	220	245			243	5
**103**	235	235	215	280	265	235	240				
**103**	230	230	210	280	260	225	245				
**110**	225	230	215	280	250	225	215			234	4
**110**	220	225	210	270	260	235	220				
**110**	220	220	220	270	255	230	220				
**117**	240	235	240	255	260	220	255			244	3
**117**	240	245	235	245	260	230	255				
**117**	240	240	235	245	270	230	245				
**124**	240	250	245	275	255	245	240			248	2
**124**	240	250	245	270	255	240	235				
**124**	235	250	235	270	250	240	250				
**131**	235	235	255	255	260	250	245			247	5
**131**	230	230	245	330	255	250	245				
**131**	230	225	210	275	255	245	235				
**138**	225	225	215	290	245	225	220			234	5
**138**	225	225	210	280	245	230	225				
**138**	220	220	215	275	240	230	225				
**145**	235	235	210	260	230	225	225			232	4
**145**	235	230	210	270	235	220	225				
**145**	235	230	215	275	240	215	220				
**151**	210	230	210	265	255	220	220			233	4
**151**	220	220	210	265	255	230	230				
**151**	225	225	215	260	255	230	235				
**159**	245	225	210	280	235	225	235			237	4
**159**	235	235	210	280	235	225	235				
**159**	230	230	210	270	250	220	250				
**168**	250	255	230	280	240	240	260			248	3
**168**	250	245	230	275	230	240	255				
**168**	255	250	235	270	225	245	245				
**175**	235	250	230	260	235	230	260			243	3
**175**	250	235	225	260	235	235	260				
**175**	245	245	230	265	230	240	250				

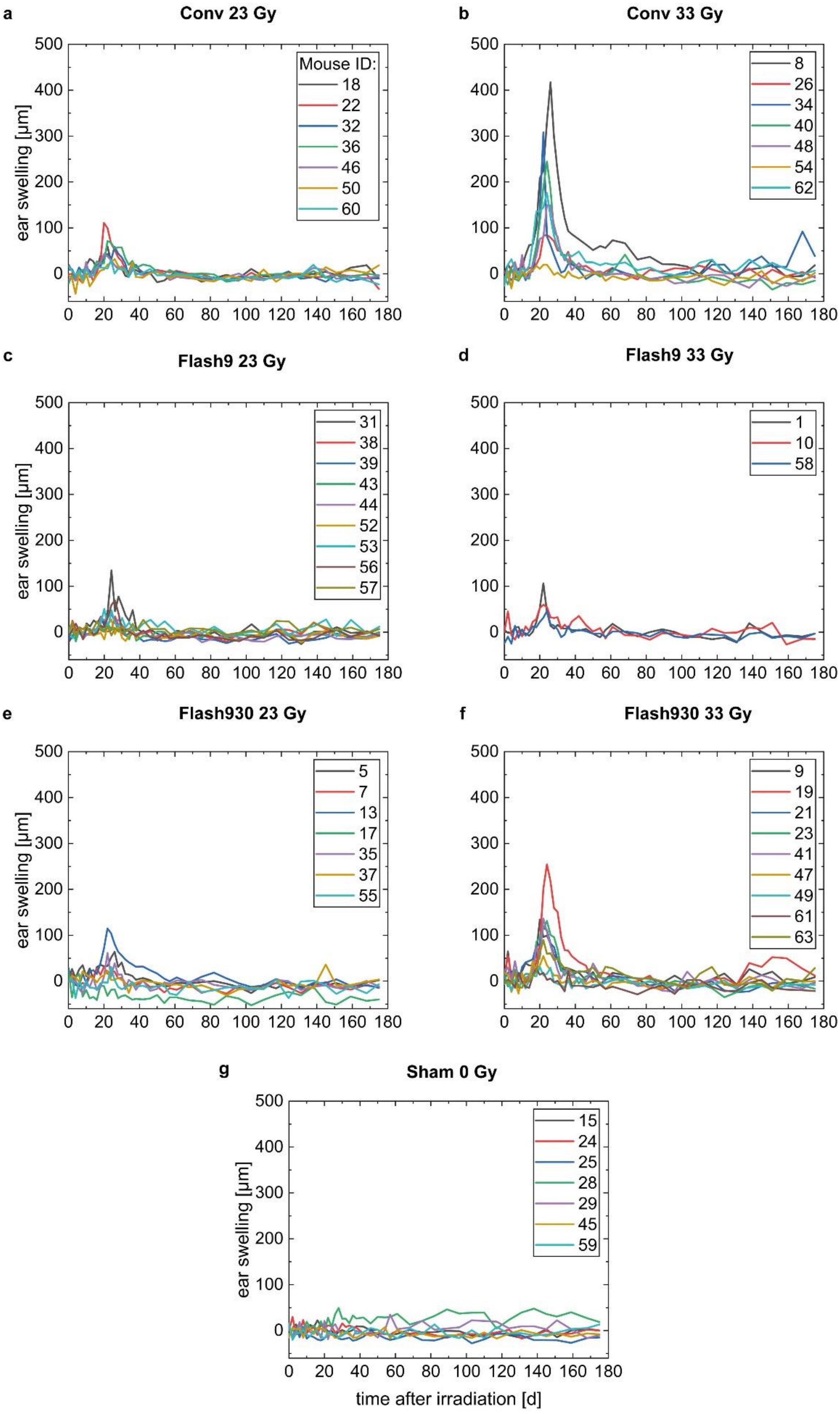

Figure S1: The ear swelling measurements during the monitoring period of 180 days after irradiation are shown for each mouse. The mice were assigned to seven irradiation groups. In these groups, three different dose rates and two different doses were studied. In a and b the mice were irradiated with a dose rate of 0.06 Gy/s, in c and d with 9.3 Gy/s and in e and f with 930 Gy/s. Mice in a, c and e received a dose of 23 Gy and in b, d and f a dose of 33 Gy. In g, the mice were sham irradiated. The ear swelling is defined as the ear thickness minus the ear thickness of the sham mice. The ear thickness was measured trice by a measuring gauge. The raw data can be find in Supplementary Table S2.

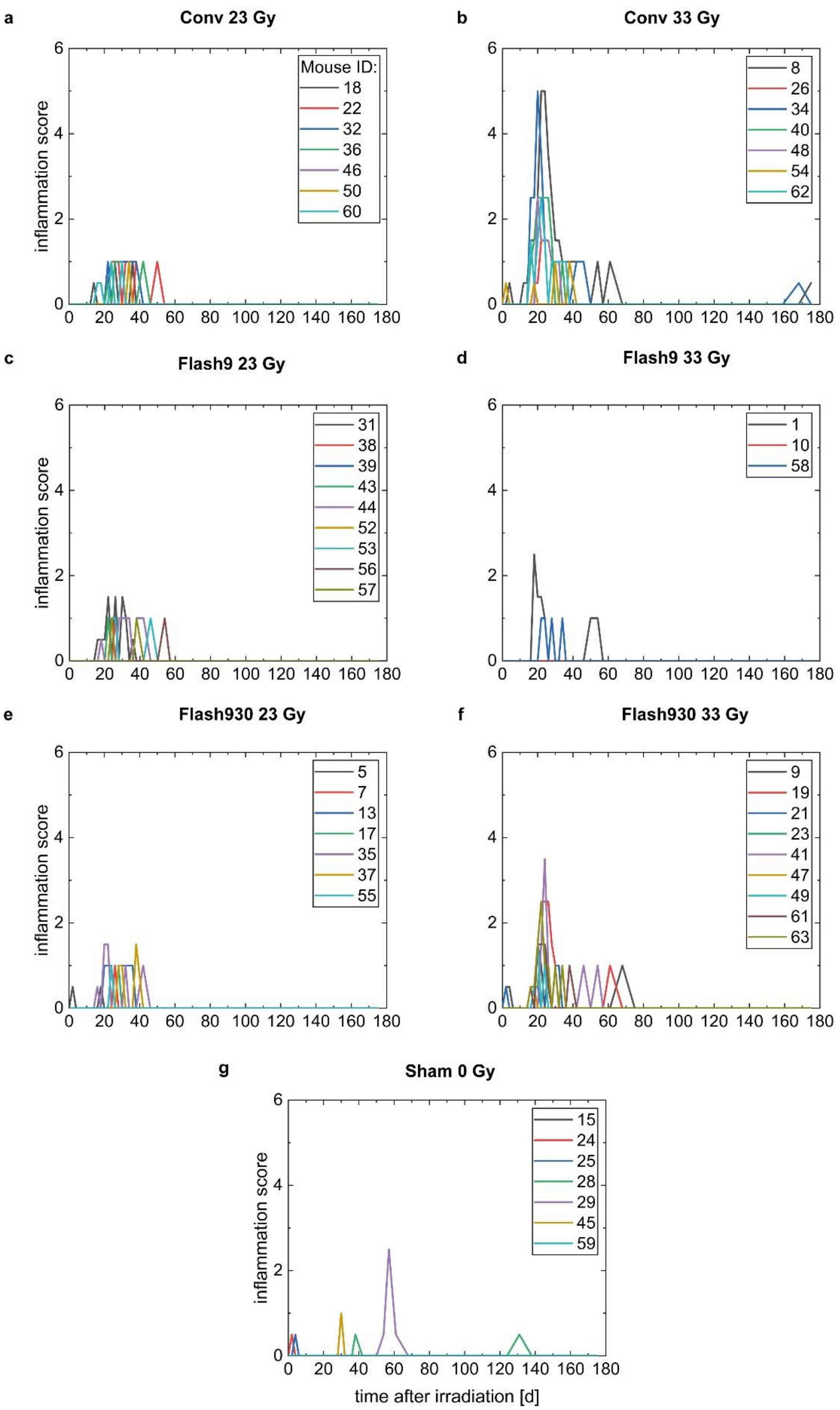

Figure S2: The inflammation scores during the monitoring period of 180 days after irradiation are shown for each mouse. The mice are assigned to seven irradiation groups. In these groups three different dose rates and two different doses were studied. In a and b the mice were irradiated with a dose rate of 0.06 Gy/s, in c and d with 9.3 Gy/s and in e and f with 930 Gy/s. Mice in a, c and e received a dose of 23 Gy and in b, d and f a dose of 33 Gy. In g, the mice were sham irradiated. The inflammation score is the sum of the erythema score and the desquamation score according to Table 1. The raw data can be find in Supplementary Table S2.

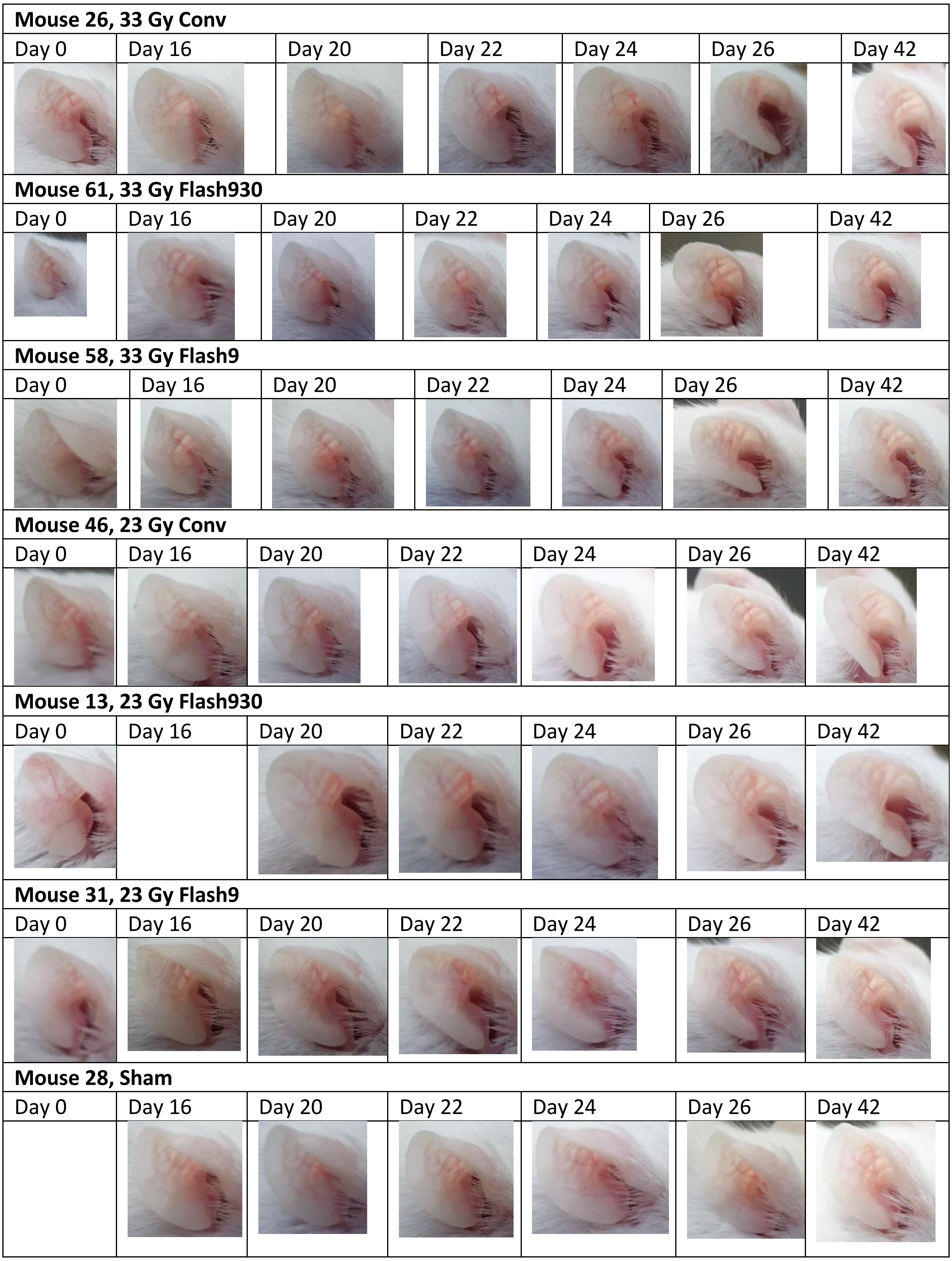

Figure S3: Photographs of the right ears of one mouse in each group for different time points. Because of the stress the photos cause the animals, not all of them could be photographed for animal welfare reasons.

Table S3: The measurements of the qPCR are listed for week 1, week 3, week 4, and the blood sampling at determination day (FIN). CT-tech is the mean of the three replica measured for every blood sample at a certain time point. CT-bio is the mean of every measured cytokine or house-keeping gene in one of the four groups (sham, Flash930 33 Gy, Flash9 33 Gy and Conv 33 Gy) at a certain time point. CT-HK is the mean of cT-bio of the two house-keeping genes for every group at a certain time point. ΔcT is calculated by subtracting the cT-HK of a group at a certain time point from the cT-bio of a cytokine of a group at a certain time point. ΔΔcT is calculated by subtracting the ΔcT of the sham group of a cytokine at a certain time point from the ΔcT of one of the irradiated groups of a cytokine at a certain time point. The fold change (FC) is calculated by Equation 1 and the log2 fold change by Equation 2.

Week 1	Gene	Mouse ID	Tech. Rep.	Ø cT-tech	Ø cT-bio	Ø cT-HK	ΔcT (primer-HK)	ΔΔcT (ir-sham)	Fold change	Log2 fold change	StDv
sham	B2M	24	22.7	23.0	22.6	24.2					
			23.0								
			23.3								
		25	22.2	21.7							
			21.4								
			21.7								
		28	22.1	22.1							
			22.3								
			21.9								
		45	23.4	23.5							
			23.4								
			23.7								
	Gapdh	24	26.3	26.3	25.9						
			26.3								
			26.3								
		25	25.1	25.5							
			25.1								
			26.3								
		28	25.6	25.8							
			25.8								
			25.8								
		45	26.5	26.0							
			25.9								
			25.8								
	TGFbeta	24	26.0	26.0	25.9		1.6				
			25.8								
			26.0								
		25	26.0	25.8							
			25.5								
			25.9								
		28	25.8	25.7							
			25.7								
			25.6								
		45	26.3	26.1							
			25.9								
			26.0								
	TNF	24	33.6	33.4	33.6		9.3				

			34.4					
			32.2					
		25	34.5	33.1				
			Undetermined					
			31.8					
		28	32.3	32.9				
			32.0					
			34.5					
		45	34.5	34.9				
			35.3					
			Undetermined					
	IL1a	24	37.3	35.2	34.8		10.6	
			34.4					
			34.0					
		25	33.8	34.7				
			35.9					
			34.5					
		28	32.6	33.0				
			33.3					
			Undetermined					
		45	Undetermined	36.3				
			37.8					
			34.8					
	IL1b	24	25.8	25.7	25.6		1.4	
			25.7					
			25.6					
		25	25.3	25.5				
			25.9					
			25.3					
		28	26.0	25.7				
			25.7					
			25.5					
		45	25.7	25.5				
			25.5					
			25.3					
Flash930 33 Gy	B2M	9	22.7	22.7	22.0	24.1		
			22.5					
			22.7					
		19	22.7	22.8				
			22.7					
			22.9					
		21	22.6	22.5				
			22.3					
			22.5					
		23	22.8	23.0				
			22.7					

			23.4								
		41	21.0	21.0							
			21.2								
			20.9								
		47	20.9	20.9							
			20.9								
			20.9								
		49	21.6	21.4							
			21.4								
			21.3								
	GAPDH	9	26.9	26.7	26.2						
			26.8								
			26.3								
		19	25.9	26.3							
			26.4								
			26.5								
		21	26.2	26.2							
			26.3								
			26.0								
		23	26.6	26.7							
			26.7								
			26.8								
		41	25.5	25.5							
			25.6								
			25.4								
		47	25.6	25.4							
			25.4								
			25.2								
		49	26.4	26.4							
			26.3								
			26.4								
	TGFbeta	9	26.3	26.3	26.0		1.9	0.3	0.8	-0.3	0.7
			26.3								
			26.3								
		19	26.2	26.4							
			26.6								
			26.5								
		21	26.0	26.4							
			26.7								
			26.4								
		23	26.5	26.6							
			26.4								
			27.0								
		41	25.6	25.2							
			25.0								

			25.0								
		47	24.7	24.9							
			25.0								
			24.9								
		49	26.2	26.2							
			26.2								
			26.2								
	TNF	9	32.5	32.5	32.7		8.6	-0.7	1.7	0.7	1.1
			32.1								
			32.9								
		19	35.0	34.4							
			32.9								
			35.2								
		21	32.5	32.7							
			32.4								
			33.3								
		23	34.2	33.8							
			33.7								
			33.5								
		41	31.7	32.0							
			32.1								
			32.1								
		47	30.9	31.2							
			31.5								
			31.0								
		49	32.6	32.4							
			32.6								
			32.1								
	IL1A	9	33.7	33.6	33.5		9.4	-1.2	2.3	1.2	0.4
			32.7								
			34.5								
		19	33.4	33.6							
			34.2								
			33.1								
		21	32.5	33.9							
			33.6								
			35.7								
		23	33.2	33.1							
			32.5								
			33.7								
		41	31.9	32.9							
			33.3								
			33.5								
		47	32.5	33.3							
			34.3								
			33.0								

		49	34.5	33.9							
			33.8								
			33.2								
	IL1B	9	25.3	24.9	25.4		1.3	-0.1	1.0	0.1	0.6
			24.9								
			24.5								
		19	25.7	26.0							
			26.2								
			25.9								
		21	26.1	26.3							
			26.4								
			26.4								
		23	25.1	25.2							
			25.1								
			25.3								
		41	24.7	24.6							
			24.5								
			24.7								
		47	25.6	25.5							
			25.6								
			25.5								
		49	25.3	25.2							
			25.3								
			25.2								
	B2M	26	22.9	22.8	22.6	24.3					
			22.8								
			22.8								
		34	22.4	22.4							
			22.4								
			22.5								
		40	22.9	22.8							
			22.8								
			22.9								
		48	22.9	22.7							
			22.7								
			22.6								
		54	22.4	22.2							
			22.1								
			21.9								
	GAPDH	26	26.6	26.7	26.1						
			26.5								
			27.1								
		34	26.0	26.1							
			26.2								
			25.9								
**Conv 33 Gy**		40	26.4	26.3							

			26.3								
			26.2								
		48	25.7	25.9							
			26.1								
			25.9								
		54	25.4	25.6							
			25.7								
			25.6								
	TGFbeta	26	26.1	25.9	25.9		1.5	-0.1	1.1	0.1	0.3
			25.8								
			25.9								
		34	25.9	25.8							
			25.8								
			25.7								
		40	26.0	26.0							
			25.9								
			26.1								
		48	26.1	26.3							
			26.4								
			26.3								
		54	25.2	25.3							
			25.4								
			25.4								
	TNF	26	33.7	33.1	32.9		8.6	-0.8	1.7	0.8	0.7
			32.9								
			32.6								
		34	32.2	33.1							
			33.5								
			33.8								
		40	34.0	33.8							
			33.6								
			33.9								
		48	32.9	32.6							
			31.5								
			33.2								
		54	31.5	32.1							
			32.4								
			32.2								
	IL1A	26	33.2	33.7	34.2		9.8	-0.7	1.7	0.7	0.8
			34.4								
			33.5								
		34	35.2	34.2							
			34.0								
			33.3								
		40	36.9	35.0							

			34.4								
			33.6								
		48	32.6	33.0							
			33.2								
			33.3								
		54	35.0	34.9							
			34.8								
			35.0								
	IL1B	26	26.1	26.0	25.6		1.2	-0.1	1.1	0.1	0.6
			26.0								
			26.0								
		34	25.0	25.3							
			25.3								
			25.5								
		40	26.0	26.1							
			26.2								
			26.2								
		48	25.7	25.7							
			25.6								
			25.8								
		54	24.8	24.8							
			24.9								
			24.7								
**Flash9 33 Gy**	B2M	1	23.1	22.9	22.4	24.0					
			22.8								
			22.8								
		58	21.9	21.8							
			21.8								
			21.6								
	GAPDH	1	25.9	26.0	25.7						
			25.9								
			26.2								
		58	25.3	25.4							
			25.5								
			25.4								
	TGFbeta	1	26.6	26.6	26.0		1.9	0.3	0.8	-0.3	1.0
			26.6								
			26.7								
		58	25.2	25.3							
			25.4								
			25.2								
	TNF	1	33.3	32.9	32.8		8.8	-0.5	1.4	0.5	0.1
			32.4								
			33.0								
		58	31.8	32.8							

			34.4								
			32.2								
	IL1A	1	35.6	35.1	34.6		10.5	0.0	1.0	0.0	0.8
			34.9								
			34.8								
		58	32.4	34.0							
			33.9								
			35.7								
	IL1B	1	26.6	26.6	25.6		1.5	0.2	0.9	-0.2	1.5
			27.0								
			26.3								
		58	24.4	24.5							
			24.5								
			24.6								
**Week 3**	**Gene**	**Mouse ID**	**Tech. Rep.**	**Ø cT-tech**	**Ø cT-bio**	**Ø cT-HK**	**ΔcT (primer-HK)**	**ΔΔcT (ir-sham)**	**Fold change**	**Log2 fold change**	**StdV**
**sham**	B2M	15	22.4	22.3	23.0	24.7					
			22.2								
			22.3								
		24	22.1	22.0							
			22.0								
			22.0								
		25	22.5	22.4							
			22.4								
			22.3								
		28	22.2	22.1							
			22.0								
			22.2								
		29	24.4	24.2							
			23.8								
			24.4								
		45	23.8	23.9							
			23.5								
			24.6								
		59	24.1	24.2							
			24.3								
			24.2								
	Gapdh	15	26.3	26.5	26.4						
			26.4								
			26.7								
		24	25.7	25.8							
			25.7								
			26.1								
		25	26.3	26.3							
			26.3								

			26.4					
		28	26.5	26.3				
			26.2					
			26.0					
		29	26.7	26.6				
			26.7					
			26.6					
		45	26.0	26.1				
			26.2					
			26.1					
		59	26.9	26.8				
			26.8					
			26.7					
	TGFbeta	15	26.4	25.9	25.7		1.5	
			25.6					
			25.7					
		24	25.4	25.2				
			25.0					
			25.1					
		25	25.8	26.0				
			25.9					
			26.2					
		28	25.9	25.7				
			25.5					
			25.6					
		29	26.3	26.1				
			26.0					
			26.0					
		45	25.0	25.1				
			25.3					
			25.0					
		59	26.0	26.1				
			26.4					
			26.0					
	TNF	15	31.8	31.6	32.6		8.4	
			31.6					
			31.3					
		24	32.3	31.9				
			31.5					
			31.9					
		25	31.6	31.6				
			31.8					
			31.5					
		28	32.2	31.5				
			31.3					

			31.2					
		29	33.9	35.6				
			39.3					
			33.6					
		45	31.8	31.5				
			31.2					
			31.6					
		59	34.9	34.6				
			Undetermined					
			34.3					
	IL1A	15	33.4	33.1	33.9		9.7	
			32.8					
			33.2					
		24	33.3	33.0				
			32.8					
			32.8					
		25	32.5	33.2				
			33.4					
			33.9					
		28	34.5	34.5				
			33.9					
			35.0					
		29	33.6	33.5				
			33.3					
			Undetermined					
		45	Undetermined	35.6				
			34.3					
			37.0					
		59	34.2	34.3				
			36.2					
			32.7					
	IL1B	15	25.9	26.0	26.0		1.8	
			26.1					
			26.0					
		24	26.3	26.6				
			26.9					
			26.7					
		25	25.7	25.8				
			25.9					
			25.7					
		28	25.8	26.3				
			26.5					
			26.5					
		29	24.9	25.0				
			25.3					
			25.0					

		45	26.0	25.9				
			26.2					
			25.6					
		59	26.5	26.5				
			26.2					
			26.9					
**Flash930 33 Gy**	B2M	9	21.4	21.5	22.7	24.6		
			21.4					
			21.8					
		21	22.8	23.0				
			23.1					
			22.9					
		23	22.4	21.9				
			21.6					
			21.9					
		41	21.6	21.6				
			21.6					
			21.7					
		47	23.5	23.7				
			23.6					
			24.1					
		49	23.0	23.0				
			23.2					
			22.9					
		61	22.2	22.3				
			22.2					
			22.5					
		63	24.0	24.2				
			23.9					
			24.8					
	GAPDH	9	25.9	25.8	26.5			
			25.5					
			25.8					
		21	27.2	27.2				
			27.1					
			27.2					
		23	26.4	26.2				
			26.2					
			25.9					
		41	26.3	25.9				
			25.9					
			25.6					
		47	27.2	27.3				
			27.3					
			27.3					
		49	26.0	26.0				

			26.0								
			25.9								
		61	26.2	26.2							
			26.6								
			25.9								
		63	28.3	27.7							
			27.5								
			27.4								
	TGFbeta	9	25.5	25.7	26.6		26.6	25.2	0.0	-25.2	0.8
			25.5								
			26.1								
		21	27.6	27.2							
			27.2								
			26.8								
		23	26.4	26.4							
			26.3								
			26.4								
		41	25.8	25.9							
			25.8								
			26.1								
		47	27.2	27.4							
			27.4								
			27.6								
		49	26.3	26.3							
			26.3								
			26.3								
		61	26.2	26.1							
			26.0								
			26.2								
		63	28.0	28.1							
			27.9								
			28.3								
	TNF	9	31.9	32.7	33.3		33.3	24.9	0.0	-24.9	1.3
			32.5								
			33.8								
		21	33.0	33.3							
			33.2								
			33.8								
		23	32.1	33.3							
			32.7								
			35.0								
		41	32.5	32.3							
			31.4								
			32.9								
		47	33.7	34.3							

			34.8								
			34.4								
		49	33.0	32.4							
			32.4								
			32.0								
		61	31.4	31.9							
			32.4								
			31.8								
		63	32.9	35.9							
			39.0								
			Undetermined								
	IL1A	9	32.8	32.7	33.9		33.9	24.3	0.0	-24.3	1.0
			33.3								
			32.2								
		21	33.4	33.7							
			32.7								
			34.9								
		23	33.7	33.6							
			34.0								
			33.1								
		41	32.6	33.1							
			32.3								
			34.4								
		47	38.3	36.0							
			35.2								
			34.5								
		49	32.7	33.6							
			33.8								
			34.4								
		61	34.8	34.1							
			34.7								
			32.9								
		63	33.0	34.4							
			34.8								
			35.4								
	IL1B	9	25.9	25.8	26.4		26.4	24.7	0.0	-24.7	1.1
			25.8								
			25.8								
		21	26.9	26.8							
			26.8								
			26.7								
		23	25.3	25.1							
			24.9								
			25.1								
		41	25.7	26.0							
			26.3								

			25.9								
		47	28.0	28.3							
			28.5								
			28.5								
		49	25.5	25.5							
			25.5								
			25.4								
		61	26.6	26.8							
			26.6								
			27.0								
		63	27.3	27.3							
			27.2								
			27.5								
Conv 33 Gy	B2M	8	22.4	22.4	22.4	24.1					
			22.4								
			22.3								
		26	23.2	23.1							
			22.9								
			23.0								
		34	22.8	22.8							
			22.8								
			22.8								
		40	21.4	21.3							
			21.3								
			21.3								
		48	21.9	21.9							
			22.2								
			21.6								
		54	22.8	22.8							
			22.7								
			22.9								
		62	22.5	22.4							
			22.6								
			22.2								
	GAPDH	8	25.9	26.1	25.8						
			26.8								
			25.6								
		26	26.5	26.5							
			26.4								
			26.4								
		34	25.9	26.0							
			25.9								
			26.2								
		40	24.2	24.4							
			24.6								
			24.3								

		48	25.2	25.1							
			25.3								
			25.0								
		54	26.4	26.4							
			26.3								
			26.6								
		62	25.9	26.1							
			25.9								
			26.5								
	TGFbeta	8	26.0	25.8	25.8		25.8	24.3	0.0	-24.3	0.7
			25.7								
			25.8								
		26	26.6	26.5							
			Undetermined								
			26.4								
		34	25.8	25.8							
			25.9								
			25.7								
		40	24.2	24.4							
			24.4								
			24.5								
		48	25.7	25.5							
			25.4								
			25.3								
		54	26.6	26.5							
			26.8								
			26.2								
		62	26.2	26.2							
			26.0								
			26.4								
	TNF	8	32.5	34.1	32.9		32.9	24.5	0.0	-24.5	1.4
			35.2								
			34.5								
		26	34.4	33.2							
			32.5								
			32.7								
		34	33.5	33.1							
			32.8								
			32.8								
		40	30.4	30.5							
			30.4								
			30.7								
		48	31.9	32.1							
			32.0								
			32.4								

		54	38.1	34.8							
			32.8								
			33.4								
		62	32.7	32.6							
			32.1								
			32.8								
	IL1A	8	33.4	33.1	34.1		34.1	24.5	0.0	-24.5	1.1
			33.9								
			32.2								
		26	33.6	34.7							
			34.9								
			35.5								
		34	36.2	36.3							
			34.1								
			38.8								
		40	32.6	33.3							
			33.4								
			34.0								
		48	34.8	34.0							
			33.4								
			33.6								
		54	32.8	34.1							
			34.8								
			34.6								
		62	32.9	33.5							
			33.0								
			34.5								
	IL1B	8	25.5	25.4	26.2		26.2	24.4	0.0	-24.4	1.1
			25.4								
			25.4								
		26	27.2	27.5							
			27.7								
			27.5								
		34	25.9	26.3							
			26.6								
			26.5								
		40	25.4	25.0							
			24.8								
			24.9								
		48	25.3	25.3							
			25.3								
			25.2								
		54	27.5	27.8							
			27.5								
			28.3								
		62	26.2	26.2							

			26.1								
			26.3								
Flash9 33 Gy	B2M	1	19.4	19.3	21.8	23.9					
			19.2								
			19.3								
		10	23.5	23.4							
			23.3								
			23.3								
		58	22.9	22.8							
			22.9								
			22.7								
	GAPDH	1	24.1	23.9	25.9						
			23.9								
			23.8								
		10	26.8	27.1							
			27.2								
			27.4								
		58	26.8	26.7							
			27.2								
			26.2								
	TGFbeta	1	25.2	24.7	26.2		26.2	24.7	0.0	-24.7	1.3
			24.5								
			24.6								
		10	27.4	27.4							
			27.4								
			27.4								
		58	26.4	26.3							
			26.3								
			26.3								
	TNF	1	30.3	30.9	32.9		32.9	24.5	0.0	-24.5	1.7
			30.6								
			31.7								
		10	35.2	34.0							
			32.8								
			Undetermined								
		58	32.9	33.7							
			Undetermined								
			34.6								
	IL1A	1	34.3	32.9	34.0		34.0	24.3	0.0	-24.3	1.7
			31.9								
			32.4								
		10	33.5	33.2							
			33.4								
			32.5								
		58	33.1	35.9							

			37.9								
			36.6								
	IL1B	1	23.9	23.8	26.6		26.6	24.8	0.0	-24.8	2.5
			23.8								
			23.8								
		10	27.5	27.3							
			27.0								
			27.3								
		58	28.9	28.7							
			28.8								
			28.4								
**Week 4**	**Gene**	**Mouse ID**	**Tech. Rep.**	**Ø cT-tech**	**Ø cT-bio**	**Ø cT-HK**	**ΔcT (primer-HK)**	**ΔΔcT (ir-sham)**	**Fold change**	**Log2 fold change**	**StdV**
**sham**	B2M	15	23.0	22.5	21.6	23.5					
			22.1								
			22.4								
		24	21.9	21.9							
			22.0								
			21.8								
		25	21.5	22.0							
			22.2								
			22.2								
		28	20.9	21.0							
			21.1								
			21.0								
		29	21.7	21.8							
			21.8								
			21.7								
		45	21.2	21.2							
			21.5								
			21.0								
		59	21.0	20.9							
			20.7								
			20.9								
	Gapdh	15	25.4	29.6	25.4						
			38.4								
			25.0								
		24	24.8	24.8							
			24.9								
			24.5								
		25	25.5	25.3							
			25.2								
			25.1								
		28	23.7	23.7							
			24.0								

			23.6					
		29	25.1	25.2				
			25.2					
			25.3					
		45	24.4	24.5				
			24.6					
			24.5					
		59	24.4	24.5				
			24.8					
			24.3					
	TGFbeta	15	25.4	25.6	25.3		1.0	
			25.7					
			25.6					
		24	24.7	24.6				
			24.6					
			24.5					
		25	25.6	25.5				
			25.2					
			25.5					
		28	25.1	24.6				
			23.9					
			24.9					
		29	25.8	25.8				
			25.8					
			25.7					
		45	25.5	25.5				
			25.7					
			25.5					
		59	25.2	25.2				
			25.3					
			25.3					
	TNF	15	32.4	32.2	31.9		7.7	
			32.3					
			31.8					
		24	30.2	30.5				
			30.4					
			30.8					
		25	31.7	32.3				
			33.2					
			31.9					
		28	31.3	31.0				
			30.6					
			31.1					
		29	32.6	32.3				
			31.9					

			32.4					
		45	31.6	32.6				
			33.6					
			32.7					
		59	Undetermined	32.6				
			33.4					
			31.8					
	IL1A	15	33.6	33.4	33.8		9.5	
			32.5					
			34.3					
		24	33.4	32.8				
			32.6					
			32.2					
		25	34.4	33.4				
			32.9					
			32.9					
		28	31.9	33.1				
			33.0					
			34.5					
		29	33.3	33.9				
			34.3					
			34.1					
		45	33.5	33.2				
			34.0					
			32.0					
		59	38.8	36.5				
			37.8					
			32.9					
	IL1B	15	25.7	25.6	25.5		1.2	
			25.4					
			25.6					
		24	25.0	24.8				
			24.7					
			24.6					
		25	26.3	26.0				
			25.8					
			25.9					
		28	25.4	25.7				
			25.5					
			26.3					
		29	26.7	26.4				
			26.2					
			26.3					
		45	24.6	24.5				
			24.8					
			24.1					

		59	25.3	25.1				
			24.9					
			25.1					
Flash930 33 Gy	B2M	9	23.3	22.9	21.9	23.6		
			23.1					
			22.4					
		19	22.5	22.6				
			22.7					
			22.4					
		21	21.0	21.0				
			21.2					
			20.9					
		23	21.5	21.4				
			21.4					
			21.4					
		41	21.8	21.7				
			21.6					
			21.7					
		47	21.3	21.3				
			21.4					
			21.2					
		49	22.2	22.2				
			22.2					
			22.2					
		61	20.9	20.9				
			20.7					
			21.0					
		63	22.8	22.9				
			22.7					
			23.2					
	GAPDH	9	25.6	25.6	25.4			
			25.6					
			25.7					
		19	26.1	26.2				
			26.2					
			26.2					
		21	24.9	24.8				
			24.6					
			24.8					
		23	25.2	25.5				
			25.6					
			25.8					
		41	25.1	25.1				
			25.1					
			25.1					
		47	24.7	24.6				

			24.7								
			24.5								
		49	25.4	25.4							
			25.4								
			25.3								
		61	24.2	24.3							
			24.3								
			24.3								
		63	26.7	26.9							
			26.7								
			27.3								
	TGFbeta	9	25.6	25.5	25.3		25.3	24.2	0.0	-24.2	0.6
			25.5								
			25.5								
		19	25.7	25.8							
			25.9								
			25.8								
		21	24.4	24.5							
			24.5								
			24.5								
		23	25.4	25.3							
			25.2								
			25.2								
		41	25.6	25.5							
			25.4								
			25.4								
		47	24.7	24.8							
			24.8								
			24.8								
		49	25.4	25.5							
			25.5								
			25.5								
		61	24.4	24.5							
			24.4								
			24.6								
		63	26.3	26.1							
			26.0								
			26.0								
	TNF	9	30.8	31.8	31.8		31.8	24.1	0.0	-24.1	0.8
			32.8								
			31.9								
		19	32.5	32.9							
			33.6								
			32.6								
		21	31.7	31.6							

			31.5								
			31.6								
		23	31.5	31.9							
			32.0								
			32.3								
		41	32.1	32.2							
			32.9								
			31.5								
		47	30.7	30.6							
			30.7								
			30.5								
		49	30.8	31.9							
			32.4								
			32.4								
		61	30.4	30.7							
			30.4								
			31.4								
		63	32.4	32.7							
			34.3								
			31.5								
	IL1A	9	33.8	33.7	34.4		34.4	24.9	0.0	-24.9	1.4
			32.5								
			34.9								
		19	33.8	35.7							
			33.9								
			39.5								
		21	32.9	33.3							
			33.7								
			33.3								
		23	33.2	33.1							
			33.9								
			32.2								
		41	33.1	32.8							
			32.4								
			33.0								
		47	33.2	33.8							
			33.0								
			35.2								
		49	33.9	34.8							
			32.9								
			37.6								
		61	Undetermined	36.6							
			37.9								
			35.3								
		63	36.9	36.2							
			33.5								

			38.4								
	IL1B	9	26.7	25.9	25.7		25.7	24.5	0.0	-24.5	0.5
			25.7								
			25.4								
		19	26.9	26.8							
			26.9								
			26.6								
		21	24.8	25.0							
			24.9								
			25.3								
		23	25.4	25.6							
			25.6								
			25.7								
		41	25.2	25.3							
			25.2								
			25.3								
		47	25.5	25.5							
			25.5								
			25.4								
		49	25.6	25.6							
			25.5								
			25.6								
		61	25.4	25.4							
			25.4								
			25.5								
		63	25.7	26.0							
			26.2								
			26.2								
**Conv 33 Gy**	B2M	26	23.6	23.2	21.9	23.3					
			23.3								
			22.6								
		34	22.3	22.1							
			22.1								
			21.8								
		40	22.2	22.1							
			22.2								
			21.7								
		48	21.0	21.0							
			21.0								
			21.1								
		54	21.8	21.6							
			21.6								
			21.4								
		62	21.6	21.3							
			21.4								
			21.0								

	GAPDH	26	25.1	25.3	24.8						
			25.4								
			25.4								
		34	24.9	25.3							
			25.4								
			25.6								
		40	24.9	24.8							
			24.7								
			24.8								
		48	23.8	23.9							
			24.0								
			23.9								
		54	24.8	24.8							
			24.7								
			24.8								
		62	24.8	24.7							
			24.8								
			24.5								
	TGFbeta	26	26.0	25.6	25.2		1.2	0.2	0.9	-0.2	0.8
			25.5								
			25.2								
		34	26.8	26.5							
			27.0								
			25.6								
		40	25.0	24.9							
			24.8								
			24.9								
		48	23.8	24.2							
			24.2								
			24.7								
		54	25.2	25.0							
			24.9								
			25.0								
		62	24.9	25.2							
			25.2								
			25.4								
	TNF	26	32.0	31.6	31.5		7.5	-0.2	1.2	0.2	0.4
			31.6								
			31.1								
		34	31.3	31.4							
			31.1								
			31.9								
		40	31.5	31.2							
			31.3								
			30.8								

		48	30.7	30.9							
			31.0								
			30.9								
		54	30.8	31.7							
			32.9								
			31.4								
		62	31.9	32.2							
			32.3								
			32.3								
	IL1A	26	34.7	33.4	33.0		9.0	-0.5	1.4	0.5	0.5
			32.5								
			32.9								
		34	36.4	33.6							
			31.9								
			32.5								
		40	32.2	32.3							
			32.3								
			32.5								
		48	32.3	32.5							
			32.7								
			32.5								
		54	33.6	32.9							
			32.2								
			32.9								
		62	33.0	33.4							
			34.5								
			32.9								
	IL1B	26	26.0	26.0	25.3		1.3	0.1	0.9	-0.1	0.7
			26.1								
			25.9								
		34	25.6	26.0							
			26.0								
			26.4								
		40	25.7	25.8							
			25.8								
			25.8								
		48	24.6	24.6							
			24.6								
			24.6								
		54	24.7	24.6							
			24.5								
			24.5								
		62	25.1	24.9							
			25.0								
			24.5								

Flash9 33 Gy	B2M	1	21.5	21.6	21.9	23.3					
			21.6								
			21.6								
		10	22.9	22.8							
			22.9								
			22.7								
		58	21.4	21.2							
			21.1								
			21.2								
	GAPDH	1	24.8	24.8	24.8						
			24.6								
			24.9								
		10	25.7	25.7							
			25.6								
			25.7								
		58	23.8	23.8							
			23.9								
			23.9								
	TGFbeta	1	24.7	25.1	25.3		25.3	24.2	0.0	-24.2	1.0
			25.6								
			24.9								
		10	26.2	26.3							
			27.0								
			25.7								
		58	24.5	24.4							
			24.5								
			24.3								
	TNF	1	31.3	30.8	30.9		30.9	23.2	0.0	-23.2	0.9
			30.7								
			30.4								
		10	31.6	31.9							
			32.1								
			31.9								
		58	30.2	30.1							
			29.6								
			30.4								
	IL1A	1	34.9	33.7	34.2		34.2	24.7	0.0	-24.7	1.4
			32.3								
			34.0								
		10	36.4	35.7							
			36.4								
			34.5								
		58	33.7	33.1							
			32.8								
			32.7								

	IL1B	1	26.0	26.0	26.4		26.4	25.2	0.0	-25.2	0.3
			26.2								
			26.0								
		10	26.4	26.5							
			26.5								
			26.6								
		58	26.6	26.6							
			26.6								
			26.6								
**Week FIN**	**Gene**	**Mouse ID**	**Tech. Rep.**	**Ø cT-tech**	**Ø cT-bio**	**Ø cT-HK**	**ΔcT (primer-HK)**	**ΔΔcT (ir-sham)**	**Fold change**	**Log2 fold change**	**StdV**
sham	B2M	15	20.5	20.2	21.5	23.5					
			20.1								
			19.9								
		24	23.2	22.1							
			21.5								
			21.7								
		25	21.3	21.2							
			20.8								
			21.4								
		28	20.4	20.3							
			20.3								
			20.2								
		45	22.6	22.9							
			22.9								
			23.2								
		59	22.6	22.4							
			22.3								
			22.2								
	Gapdh	15	24.5	24.6	25.5						
			24.5								
			24.7								
		24	26.6	26.5							
			26.3								
			26.5								
		25	25.6	25.6							
			25.6								
			25.7								
		28	23.6	23.8							
			24.6								
			23.4								
		45	26.7	26.2							
			26.4								
			25.7								
		59	26.5	26.5							

			26.6					
			26.6					
	TGFbeta	15	24.7	24.7	26.1		1.8	
			24.6					
			24.9					
		24	27.2	27.0				
			27.0					
			26.7					
		25	26.2	26.1				
			25.8					
			26.3					
		28	26.0	25.6				
			25.3					
			25.5					
		45	26.4	26.3				
			26.3					
			26.3					
		59	26.6	26.7				
			26.8					
			26.7					
	TNF	15	32.5	31.6	32.2		8.0	
			31.6					
			30.7					
		24	32.3	32.2				
			31.5					
			32.8					
		25	32.7	32.4				
			31.5					
			33.1					
		28	32.9	31.8				
			31.2					
			31.3					
		45	31.8	31.6				
			31.7					
			31.2					
		59	32.9	33.9				
			33.9					
			34.8					
	IL1A	15	31.0	30.7	31.7		7.4	
			30.8					
			30.3					
		24	33.8	32.5				
			32.4					
			31.5					
		25	30.0	30.7				

			31.3					
			30.7					
		28	29.8	30.0				
			29.8					
			30.3					
		45	32.6	32.6				
			32.8					
			32.4					
		59	33.8	33.4				
			32.9					
			33.5					
	IL1B	15	25.4	25.3	26.2		2.0	
			25.3					
			25.2					
		24	28.5	28.9				
			29.5					
			28.8					
		25	25.0	25.1				
			25.0					
			25.4					
		28	25.2	25.5				
			25.5					
			25.7					
		45	25.9	25.7				
			25.7					
			25.7					
		59	26.9	26.7				
			26.7					
			26.3					
**Flash930 33 Gy**	B2M	9	21.9	21.8	22.8	24.1		
			21.8					
			21.8					
		19	21.9	22.0				
			22.4					
			21.6					
		21	22.3	22.2				
			22.2					
			22.2					
		23	22.9	22.7				
			22.7					
			22.4					
		41	22.5	22.4				
			22.5					
			22.4					
		47	23.8	23.8				
			24.0					

			23.7								
		49	23.9	23.9							
			23.6								
			24.3								
		61	23.5	23.4							
			23.3								
			23.4								
	GAPDH	9	25.0	24.9	25.3						
			24.9								
			24.9								
		19	25.6	25.4							
			25.3								
			25.2								
		21	24.8	24.7							
			24.7								
			24.7								
		23	25.7	25.9							
			26.2								
			25.9								
		41	24.6	24.6							
			24.4								
			24.9								
		47	26.2	26.1							
			25.9								
			26.2								
		49	25.5	25.5							
			25.8								
			25.1								
		61	25.8	25.5							
			25.6								
			25.1								
	TGFbeta	9	27.1	26.9	27.5		27.5	25.7	0.0	-25.7	0.8
			26.9								
			26.7								
		19	27.0	26.9							
			27.0								
			26.8								
		21	28.6	28.1							
			27.8								
			27.9								
		23	28.9	28.8							
			28.7								
			28.8								
		41	26.8	27.0							
			26.6								

			27.6								
		47	27.6	27.7							
			28.3								
			27.2								
		49	26.8	26.7							
			26.6								
			26.8								
		61	28.1	28.1							
			28.2								
			28.0								
	TNF	9	35.2	33.2	32.8		32.8	24.8	0.0	-24.8	1.3
			31.9								
			32.6								
		19	32.4	32.6							
			33.2								
			32.2								
		21	32.4	32.3							
			32.1								
			32.4								
		23	32.8	32.6							
			32.8								
			32.4								
		41	31.1	30.8							
			30.4								
			30.8								
		47	34.7	35.4							
			39.3								
			32.3								
		49	33.6	32.9							
			32.7								
			32.6								
		61	31.9	32.6							
			33.0								
			32.9								
	IL1A	9	32.5	33.3	32.8		32.8	25.4	0.0	-25.4	1.1
			34.1								
			33.3								
		19	34.1	33.8							
			33.9								
			33.3								
		21	32.1	32.3							
			31.4								
			33.3								
		23	33.8	34.0							
			33.4								

			35.0								
		41	30.8	30.8							
			30.6								
			30.9								
		47	33.8	33.6							
			33.5								
			33.6								
		49	33.8	33.0							
			32.6								
			32.8								
		61	31.9	31.8							
			31.6								
			32.0								
	IL1B	9	25.3	25.4	26.6		26.6	24.6	0.0	-24.6	1.4
			25.3								
			25.4								
		19	24.9	24.8							
			24.7								
			24.7								
		21	25.5	25.6							
			25.5								
			25.6								
		23	29.0	28.8							
			28.7								
			28.6								
		41	26.6	26.5							
			26.6								
			26.4								
		47	26.8	26.4							
			26.5								
			25.8								
		49	26.3	26.6							
			26.5								
			26.9								
		61	28.5	28.5							
			28.6								
			28.6								
**Conv 33 Gy**	B2M	8	22.3	22.4	22.2	23.6					
			22.5								
			22.2								
		26	23.6	23.6							
			23.9								
			23.3								
		40	22.3	22.1							
			22.0								

			21.9								
		48	22.4	22.3							
			22.3								
			22.3								
		54	21.7	21.8							
			21.5								
			22.3								
		62	20.6	20.7							
			20.6								
			21.0								
	GAPDH	8	25.2	25.2	25.0						
			25.2								
			25.3								
		26	26.3	26.2							
			26.3								
			26.1								
		40	24.4	24.6							
			24.7								
			24.6								
		48	24.4	24.6							
			24.8								
			24.5								
		54	26.3	26.0							
			25.7								
			25.9								
		62	23.5	23.4							
			23.5								
			23.2								
	TGFbeta	8	26.4	26.2	26.2		26.2	24.4	0.0	-24.4	0.4
			26.0								
			26.3								
		26	26.2	26.3							
			26.6								
			26.1								
		40	25.5	25.4							
			25.2								
			25.6								
		48	26.2	26.2							
			26.3								
			26.2								
		54	26.6	26.5							
			26.4								
			26.5								
		62	26.8	26.7							
			26.7								

			26.6									
	TNF	8	32.3	32.8	32.3		32.3	24.3	0.0	-24.3	0.8
			32.9								
			33.3								
		26	32.3	32.6							
			33.2								
			32.4								
		40	31.6	31.6							
			31.6								
			31.7								
		48	31.6	31.5							
			31.3								
			31.6								
		54	33.8	33.4							
			32.6								
			33.7								
		62	31.3	31.6							
			31.8								
			31.8								
	IL1A	8	31.7	32.8	31.9		31.9	24.5	0.0	-24.5	1.7
			34.1								
			32.6								
		26	33.6	34.0							
			35.0								
			33.4								
		40	33.2	32.6							
			32.0								
			32.6								
		48	31.4	31.2							
			31.3								
			30.9								
		54	31.5	31.9							
			31.5								
			32.7								
		62	29.0	28.9							
			28.9								
			28.7								
	IL1B	8	25.5	25.4	25.7		25.7	23.8	0.0	-23.8	0.8
			25.3								
			25.5								
		26	26.3	26.2							
			26.2								
			26.3								
		40	24.4	24.4							
			24.5								

			24.3								
		48	25.7	25.7							
			25.7								
			25.7								
		54	26.3	26.3							
			26.4								
			26.3								
		62	26.4	26.4							
			26.4								
			26.4								
**Flash9 33 Gy**	B2M	1	22.6	22.8	21.9	23.2					
			22.6								
			23.3								
		10	21.4	21.3							
			21.3								
			21.1								
		58	21.7	21.6							
			21.5								
			21.6								
	GAPDH	1	25.7	25.3	24.6						
			25.0								
			25.1								
		10	24.4	24.3							
			24.3								
			24.2								
		58	23.7	24.2							
			24.5								
			24.3								
	TGFbeta	1	26.7	26.8	26.2		26.2	24.3	0.0	-24.3	0.8
			26.5								
			27.3								
		10	25.7	25.3							
			25.4								
			24.8								
		58	26.3	26.4							
			26.4								
			26.4								
	TNF	1	33.6	33.5	32.4		32.4	24.4	0.0	-24.4	1.0
			33.9								
			32.8								
		10	32.5	32.2							
			32.5								
			31.7								
		58	31.9	31.4							
			31.6								

			30.8								
	IL1A	1	32.5	32.3	31.6		31.6	24.2	0.0	-24.2	0.6
			32.2								
			32.2								
		10	31.4	31.3							
			31.1								
			31.4								
		58	30.9	31.3							
			31.7								
			31.2								
	IL1B	1	27.2	27.1	26.1		26.1	24.2	0.0	-24.2	0.8
			26.9								
			27.2								
		10	25.3	25.5							
			25.7								
			25.5								
		58	25.8	25.8							
			25.9								
			25.8								

Table S4: Measurements for the irradiated blood volume estimation. The estimation was performed on trans-illuminated photographs of the mouse ears from three mice directly after irradiation. The letters S and G in the mouse ID indicate that these mice were from a previous study, where the setup and handling of mice were the same (S. Girst et al.: Proton Minibeam Radiation Therapy Reduces Side Effects in an In Vivo Mouse Ear Model, Int J Radiation Oncol Biol Phys, Vol. 95, No. 1, pp. 234e241, 2016, https://doi.org/10.1016/j.ijrobp.2015.10.020).

	Static blood volume [µl]	Blood flow volume [µl]			Total irradiated blood volume [µl]		
		Flash930	Flash9	Conv	Flash930	Flash9	Conv
mouse SG24	0.28	0.0022	0.22	34	0.28	0.50	35
mouse SG16	0.12	0.0012	0.13	20	0.12	0.24	20
mouse SG18	0.18	0.0017	0.18	27	0.19	0.36	28
mean:	0.19	0.0017	0.18	27	0.20	0.37	27
Stdev:	0.07	0.0004	0.04	7	0.07	0.11	7
